# Triterpenic Acids as Non-Competitive α-Glucosidase Inhibitors from *Boswellia elongata* with Structure-Activity Relationship: In Vitro and In Silico Studies

**DOI:** 10.3390/biom10050751

**Published:** 2020-05-12

**Authors:** Najeeb Ur Rehman, Sobia Ahsan Halim, Mohammed Al-Azri, Majid Khan, Ajmal Khan, Kashif Rafiq, Ahmed Al-Rawahi, Rene Csuk, Ahmed Al-Harrasi

**Affiliations:** 1 Natural & Medical Sciences Research Center, University of Nizwa, P.O Box 33, Birkat Al Mauz, Nizwa 616, Oman; najeeb@unizwa.edu.om (N.U.R.); sobia_halim@unizwa.edu.om (S.A.H.); malazri@unizwa.edu.om (M.A.-A.); majid.khan@unizwa.edu.om (M.K.); ajmalkhan@unizwa.edu.om (A.K.); kashifrafiq@unizwa.edu.om (K.R.); ahmed@unizwa.edu.om (A.A.-R.); 2 H. E. J. Research Institute of Chemistry, International Center for Chemical and Biological Sciences, University of Karachi, Karachi 75270, Pakistan; 3 Department of Chemistry, Abdul Wali Khan University Mardan, Mardan 23200, Pakistan; 4 Organic Chemistry, Martin-Luther-University Halle-Wittenberg, Kurt-Mothes-Str. 2, D-06120 Halle (Saale), Germany; rene.csuk@chemie.uni-halle.de

**Keywords:** *Boswellia elongata*, triterpene acids, α-glucosidase inhibitors, NMR spectroscopy, kinetics study, homology modeling, molecular docking

## Abstract

Fourteen triterpene acids, viz., three tirucallane-type (**1**–**3**), eight ursane-type (**4**–**11**), two oleanane-type (**12**, **13**) and one lupane type (**21**), along with boswellic aldehyde (**14**), α-amyrine (**15**), epi-amyrine (**16**), straight chain acid (**17**), sesquiterpene (**19**) and two cembrane-type diterpenes (**18**, **20**) were isolated, first time, from the methanol extract of *Boswellia elongata* resin. Compound (**1**) was isolated for first time as a natural product, while the remaining compounds (**2**‒**21**) were reported for first time from *B. elongata.* The structures of all compounds were confirmed by advanced spectroscopic techniques including mass spectrometry and also by comparison with the reported literature. Eight compounds (**1**–**5, 11, 19** and **20**) were further screened for in vitro α-glucosidase inhibitory activity. Compounds **3**–**5** and **11** showed significant activity against α-glucosidase with IC_50_ values ranging from 9.9–56.8 μM. Compound **4** (IC_50_ = 9.9 ± 0.48 μM) demonstrated higher inhibition followed by **11** (IC_50_ = 14.9 ± 1.31 μM), **5** (IC_50_ = 20.9 ± 0.05 μM) and **3** (IC_50_ = 56.8 ± 1.30 μM), indicating that carboxylic acid play a key role in α-glucosidase inhibition. Kinetics studies on the active compounds **3**–**5** and **11** were carried out to investigate their mechanism (mode of inhibition and dissociation constants *K_i_*). All compounds were found to be non-competitive inhibitors with *K_i_* values in the range of 7.05 ± 0.17–51.15 ± 0.25 µM. Moreover, in silico docking was performed to search the allosteric hotspot for ligand binding which is targeted by our active compounds investigates the binding mode of active compounds and it was identified that compounds preferentially bind in the allosteric binding sites of α-glucosidase. The results obtained from docking study suggested that the carboxylic group is responsible for their biologic activities. Furthermore, the α-glucosidase inhibitory potential of the active compounds is reported here for the first time.

## 1. Introduction

Diabetes mellitus (DM)—mostly characterized by high blood-glucose levels (hyperglycemia), and their complications—increases the morbidity and mortality threats for type-2 diabetes patients [1,2]. Poor control of the post-prandial glucose levels, mostly concerned with type-2 DM, leads to atherosclerosis and cardiovascular disorders [3,4]. It has been reported that about 90% of the world’s diabetic people have Type-2 DM [5]. α-Glucosidase inhibitors (AGIs) have inadequate protection, temporally recover the blood glucose levels, and improve Type-2 DM complications, together with the treatment of obesity [6,7] but accomplished with gastrointestinal side-effects like diarrhea, flatulence and abdominal discomfort [8,9,10].

Natural products are known to have anti-diabetic effects and offered plentiful exciting potentials for the future development and improvement of successful therapies [6]. Interesting previously isolated boswellic acids (BAs), bioactive components of frankincense, from the resins of *Boswellia sacra* and *B. papyrifera* demonstrated promising α-glucosidase activity [11]. Keeping in mind the side effects of the existing synthetic drugs and especially a crucial role of α-glucosidase enzyme in hyperglycemia, there is an urgent need to discover safe and effective enzyme inhibitors as an approach to effectively control the diabetic disorders.

The genus *Boswellia* (Burseraceae) consists of 23 species widespread throughout the world, mainly in Arabia, in eastern coast of Africa and in India [12,13]. Frankincense (olibanum), gum resin obtained from trees of the genus *Boswellia,* is mostly used in traditional remedies for decades [14] against fevers, dysentery, antiseptic and as an antitumor agent [15,16]. BAs (bioactive components of frankincense) are mostly isolated from the resins of *Boswellia* species and considered to have interesting pharmacological, biologic and medicinal applications against chronic colitis, asthma, inflammation, arthritis, stomach ache, ulcerative colitis and hepatitis [17,18,19].

Eight species of the genus are available in Soqotra Island. *Boswellia elongate* Balf. f. (endemic to Soqotra) is mostly found on stony soils with valuable producing frankincense [20]. *B. elongata*, one of the most important Soqotraen medicinal plants, is used traditionally to treat common cold, bronchitis, relieving fever and pain, sweetening the breath, sooth a disturb stomach, rheumatism as well as a remedy for asthma [21]. Different parts of the plant are useful in a variety of diseases like diarrhea, urinary disorder, dysentery, gonorrhea, bronchitis [13]. The essential oil of *B. elongata* was dominated by verticillol, β-caryophyllene and methyl cycloundecanecarboxylate having 52.4%, 39.1% and 7.9%, respectively [12]. Previous biologic investigation on the bark of *B. elongata* reported significant antimicrobial and antiviral activities [21,22]. Previous report on the analgesic and anti-inflammatory activities of methanolic extract further supported the traditional application of this plant in treating various diseases associated with inflammation and pain [13]. However, no report is available on the phytochemical investigations of the title resin.

## 2. Experimental

### 2.1. General

High-resolution electrospray ionisation mass spectrometry (HR-ESI-MS) spectra were recorded on Agilent 6530 LC Q-TOF (country of origin USA/EU, made in Singapore). Infra-red (IR) spectra were recorded on a ATR-Tensor 37 spectrometer, Bruker (Ettlingen, Baden-Württemberg, Germany) with wave numbers (ν) in cm^−1^. Optical rotations were measured on a KRUSS P3000 polarimeter (A. Kruss Optronic, Hamburg, Germany). The ^1^H- and ^13^C NMR spectra were recorded on nuclear magnetic resonance (NMR) spectrometer (Bruker, Zürich, Switzerland) operating at 600 MHz (150 MHz for ^13^C) using the solvent peaks as internal references (CDCl_3_, δ_H_: 7.26; δ_C_: 77.0), (CD_3_OD, δ_H_: 4.87; δ_C_: 48.5). Data were reported in the following order: chemical shift (δ) in ppm; multiplicities are indicated s = singlet, d = doublet, t = triplet, dd = doublet of doublet, m = multiplet; coupling constants (J) are in hertz (Hz). Column chromatography was carried out by using silica gel of the selected particle size of 100–200 mesh. For thin layer chromatography TLC, pre-coated aluminum sheets (silica gel 60F-254, Merck, Darmstadt, Hesse, Germany) were used. Visualization was accomplished with UV-light (254 and 366 nm) or I_2_ stain and also by spraying with the ceric sulfate reagent.

### 2.2. Plant Material and Identification

The gum resin of *B. elongata* was donated by Mr. Mohammed Khalifa (Yemen, 2017) and identified by Dr. Labib Noman from Island of Socotra. The voucher specimen (BEL/04/2017) of the sample was deposited in the herbarium of the Natural & Medical Sciences Research Center, University of Nizwa, Oman.

### 2.3. Extraction and Isolation

The air-dried powder resin of *B. elongata* (100 g) was finely extracted with MeOH (1 L) at room temperature (three times) and evaporated under reduced pressure to yield a yellow semi-solid methanol residue (66.0 g). The crude MeOH extract was successively fractionated into *n*-hexane (12.7 g), ethyl acetate (47.5 g) and aqueous (6.0 g). The *n*-hexane fraction was first subjected to column chromatography using 5%, 10%, 20%, 30% and 50% EtOAc/*n*-hexane to afford twenty-two fractions (BEHF_1–22_). Fractions (BEHF_5–10_, 10%–20% *n*-hexane/EtOAc) were further subjected to silica gel column chromatography (CC) one by one using an isocratic mobile phase to get compounds **14**–**21**.

Similarly, ethyl acetate fraction was subjected to CC using isocratic mobile phase viz., 10%, 20%, 30%, 40% and 50% AcOEt/*n*-hexane to afford sixteen fractions (BEEF_1–16_). After taking TLC, sub fraction (BEEF_4_) was further chromatographed on CC to afford three compounds **1** (5.7 mg), **2** (14.6 mg) and **3** (3.5 mg) using 20% and 30% AcOEt/*n*-hexane system as a mobile phase along with some semi-pure compounds **10** (10.5 mg) and **11** (17 mg), which were later on purified through preparative high performance liquid chromatography (HPLC) using CHCl_3_ solvent. Sub fractions BEEF_6–10_ were combined due to their similar TLC profile and further subjected on CC using *n*-hexane/AcOEt with increasing polarity (2:8, 4:6, 6:4 and 8:2) to afford compounds **4**–**9**.

### 2.4. α-Tirucalla-8, 24-Dien-3α-Acetate (1)

Compound **1:** colorless solid; UV (MeOH)λ*_max_* 218 (3.38); [α]^25^_D_ 17.8° (MeOH, c = 0.17); IR (solid)υ*_max_* 1724 (CH_3_CO), 1624 (C=C), 1446, 1366, 1215, 1020, 920 cm^−1^; ^1^HNMR (CDCl_3,_ 600 MHz): δ 5.08 (1H, t = 7.2, 5.4 Hz, H-24), 4.49 (1H, dd, J = 12.0, 4.8 Hz, H-3), 2.02 (3H, s, H-32), 1.65 (3H, s, H-26), 1.58 (3H, s, H-27), 1.62 (1H, br. s, H-5), 1.46 (1H, m. H-17), 1.39 (1H, m, H-20), 0.95 (3H, s, H-28), 0.93 (3H, d, *J* = 6.4 Hz, H-21), 0.89 (3H, s, H-19), 0.85 (6H, s, H-29/30), 0.72 (3H, s, H-18); ^13^C-NMR (CDCl_3_, 125 MHz): δ 171.0 (C-31), 133.9 (C-9), 133.6 (C-8), 131.2 (C-25), 125.2 (C-24), 80.9 (C-31), 51.0 (C-5), 50.1 (C-17), 49.9 (C-14), 44.0 (C-13), 37.8 (C-4), 37.1 (C-10), 36.3 (C-22), 36.2 (C-20), 34.9 (C-7), 30.8 (C-12), 29.8 (C-15), 28.0 (C-28), 27.9 (C-16), 27.5 (C-2), 25.7 (C-27), 24.9 (C-23), 24.3 (C-1), 24.2 (C-30), 21.4 (C-11), 21.3 (C-32), 20.1 (C-19), 18.7 (C-6), 18.6 (C-29/21), 16.6 (C-26), 15.3 (C-18); HRMS (ESI+): *m/z* 469.3292 [M + H]^+^ (calculated for C_32_H_53_O_2_: 469.3280).

5α-Tirucalla-8,24-dien-3α-ol (**2**): colorless solid; ^1^H-NMR (600 MHz, chloroform-*d*): δ 5.08 (1H, brt, 5.4, 1.2 Hz), 3.41 (1H, br.t, 3.0 Hz), 1.66 (3H, s), 1.56 (3H, s), 0.94 (3H, s), 0.93 (3H, s), 0.89 (3H, d, J = 6.0 Hz), 0.84 (6H, br.s), 0.74 (3H, s); ^13^C-NMR (150 MHz, chloroform-*d*): δ 15.5, 17.6, 18.6, 18.8, 19.9, 21.4, 22.2, 24.4, 24.9, 25.7, 25.8, 27.2, 28.0, 28.0, 29.7, 30.8, 36.3, 36.4, 37.1, 37.6, 44.0, 44.8, 55.0, 55.0, 125.2, 130.8, 133.3, 134.2; HRMS (ESI+): *m/z* 426.9714 [M + H]^+^.

3α-Acetoxytirucall-8,24-dien-21-oic acid (**3**): colorless solid; ^1^H-NMR (600 MHz, chloroform-*d*): δ 5.08 (1H, br.t, 7.2, 6.6 Hz), 4.63 (1H, br.s), 2.02 (3H, s, Ac), 1.65 (3H, s), 1.56 (3H, s), 1.24 (3H, s), 0.91 (3H, s), 0.88 (3H, s), 0.86 (3H, s), 0.84 (3H, s); ^13^C-NMR (150 MHz, chloroform-*d*): δ 15.8, 17.6, 18.5, 19.8, 21.3, 21.4, 21.8, 23.3, 24.4, 25.6, 25.9, 26.9, 27.0, 27.6, 27.0, 27.6, 28.8, 29.3, 29.6, 30.5, 32.4, 36.7, 37.1, 43.8, 45.8, 46.9, 47.4, 49.6, 123.5, 132.2, 132.9, 134.2, 170.8, 181.6; HRMS (ESI+): *m/z* 512.3652 [M + Na]^+^ (C_35_H_56_O_5_Na).

3-*O*-Acetyl-9,11-dehydro-*β*-boswellic acid (**4**): colorless solid; ^1^H-NMR (600 MHz, chloroform-*d*); δ 5.63 (1H, d, *J* = 6.0 Hz), 5.44 (1H, d, *J* = 5.4 Hz), 5.27 (1H, br.s), 2.02 (3H, s, Ac), 1.24 (3H, s), 1.19 (3H, s), 1.09 (3H, s), 0.92 (3H, s), 0.90 (3H, s), 0.84 (3H, s), 0.78 (3H, d, *J* = 5.4 Hz); ^13^C-NMR (150 MHz, chloroform-*d*): 17.3, 17.4, 19.5, 21.2, 21.5, 21.7, 23.2, 23.7, 24.3, 26.1, 28.2, 28.7, 31.1, 31.8, 33.1, 33.6, 39.0, 39.0, 39.4, 40.6, 41.3, 43.3, 46.9, 47.4, 57.3, 72.9, 116.5, 123.0, 141.6, 152.4, 170.3, 182.2; HRMS (ESI+): *m/z* 497.3655 [M + H]^+^ (C_35_H_56_O_5_Na)

9,11-Dehydro-β-boswellic acid (**5**): colorless solid material; ^1^H-NMR (600 MHz, chloroform-*d*); δ 5.63 (1H, d, *J* = 5.4 Hz), 5.43 (1H, d, *J* = 5.4 Hz), 4.06 (1H, br.t), 1.34 (3H, s), 1.16 (3H, s), 1.08 (3H, s), 0.90 (6H, s), 0.83 (3H, br.s), 0.78 (3H, d, *J* = 6.6 Hz); ^13^C-NMR (150 MHz, chloroform-*d*): δ 17.3, 17.5, 19.6, 21.5, 21.7, 23.3, 24.1, 26.1, 26.9, 28.2, 28.7, 31.2, 31.8, 32.5, 33.6, 39.0, 39.1, 39.4, 40.6, 41.3, 43.3, 46.0, 47.6, 57.3, 70.3, 116.4, 122.9, 141.5, 152.7, 183.0; HRMS (ESI+): *m/z* 455.3516 [M + H]^+^.

3-Acetyl-β-boswellic acid (β-ABA, **6**): colorless needles; ^1^H NMR (600 MHz, chloroform-*d*) 5.28 (1H, brt, H-12), 5.12 (1H, br.t, H-3), 2.07 (3H, s, H-3, Ac), 1.24 (3H, s), 1.16 (3H, s), 1.02 (3H, s), 0.90 (3H, s) 0.87 (3H, s), 0.81 (3H, d, 6.6 Hz), 0.78 (3H, d, *J* = 6.6 Hz); ^13^C NMR (150 MHz, chloroform-*d*): δ 13.3, 16.8, 17.4, 19.6, 21.3, 21.3, 23.2, 23.4, 23.6, 23.6, 26.5, 28.1, 28.7, 29.6, 31.2, 33.0, 33.8, 34.5, 37.4, 39.6, 39.7, 40.0, 41.5, 42.2, 46.5, 46.8, 50.5, 59.1, 73.2, 124.5, 139.5, 170.3, 181.2; HRMS (ESI+): *m/z* 497.3918 [M + H]^+^.

3-Acetyl 11-keto-β-boswellic acid (AKBA, **7**): ^1^H-NMR (600 MHz, chloroform-*d*): δ 5.53 (1H, br.s, H-12), 5.27 (1H, t, H-3), 2.38 (1H, s, H-9), 2.06 (3H, s, H-3 Ac), 1.30 (3H, s), 1.21 (3H, s), 1.16 (3H, s), 1.11 (3H, s), 0.92 (3H, s), 0.80 (3H, d, *J* = 7.2), 0.79 (3H, d, *J* = 6.0); ^13^C-NMR (150 MHz, chloroform-*d*): δ 13.2, 14.1, 17.4, 18.3, 18.7, 20.5, 21.0, 21.1, 21.3, 23.5, 23.8, 27.2, 27.5, 28.8, 30.9, 32.8, 33.9, 34.6, 37.3, 39.2, 39.3, 40.9, 43.7, 45.0, 46.4, 50.3, 59.0, 60.3, 60.4, 73.1, 130.4, 164.9, 170.2, 180.8, 199.3; HRMS (ESI+): *m/z* 513.3568 [M + H]+.

*β*-Boswellic acid (*β*-BA, **8**): colorless solid; ^1^H-NMR (600 MHz, chloroform-*d*); δ 5.11 (1H, br.t), 4.08 (1H, br.s), 1.31 (3H, s), 1.12 (3H, s), 1.03 (3H, s), 0.88 (6H, s), 0.81 (3H, d, J = 6.6 Hz), 0.76 (3H, d, J = 6.0 Hz); ^13^C-NMR (150 MHz, chloroform-*d*): δ 13.3, 16.8, 17.4, 19.7, 21.3, 23.2, 23.4, 24.1, 26.2, 26.5, 28.1, 28.7, 31.2, 31.1, 33.8, 33.9, 37.5, 39.6, 39.7, 40.0, 41.5, 42.3, 46.8, 47.3, 49.1, 59.5, 70.7, 124.5, 139.6, 182.6; HRMS (ESI^‒^): *m/z* 455.3996 [M − H]^+^.

11-Keto-*β*-boswellic acid (KBA, **9**)**:** colorless crystals; ^1^H-NMR (600 MHz, chloroform-*d*); δ 5.52 (1H, s), 4.05 (br.t), 1.31 (3H, s), 1.28 (3H, s), 1.15 (3H, s), 1.09 (3H, s), 0.90 (3H, s), 0.79 (3H, d, *J* = 6.0 Hz), 0.77 (3H, d, *J* = 6.6 Hz); ^13^C-NMR (150 MHz, chloroform-*d*): δ 13.2, 17.4, 18.4, 18.8, 20.5, 21.1, 24.3, 26.2, 27.2, 27.5, 28.9, 30.9, 32.9, 33.9, 34.0, 37.5, 39.3, 39.3, 40.9, 43.8, 45.1, 47.2, 48.8, 59.0, 60.4, 70.5, 130.5, 165.0, 182.0, 199.6; HRMS (ESI+): *m/z* 471.3473 [M + H]^+^.

3*α*, 11*α*-dihydroxyurs-12-en-24-oic acid (**10**): white solid; ^1^H-NMR (600 MHz, chloroform-*d*); δ 5.16 (1H, br.t), 4.22 (1H, d, br.t, J = 9.6, 6.0 Hz), 3.98 (1H, br.t), 1.27 (3H, s), 1.24 (3H, s), 1.14 (3H, s), 1.08 (3H, s), 0.96 (6H, s), 0.84 (3H, d, J = 3.0 Hz); ^13^C-NMR (150 MHz, chloroform-*d*): δ 14.8, 18.1, 18.7, 20.9, 21.7, 23.3, 25.3, 27.3, 27.6, 29.1, 29.3, 32.2, 34.8, 35.3, 37.2, 39.8, 40.8, 40.9, 42.6, 43.4, 44.5, 54.4, 59.8, 69.4, 71.6, 131.2, 142.6, 181.4; HRMS (ESI+): *m/z* 455.3463 [M + H]^+^.

11*α*-methoxy-*β*-boswellic acid (**11**): white solid; ^1^H-NMR (600 MHz, chloroform-*d*); δ 5.29 (1H, s), 4.03 (1H, s), 3.87 (1H, dd, *J* = 9.0, 2.4 Hz), 1.38 (3H, s), 1.28 (3H, s), 1.14 (3H, s), 1.07 (3H, s), 0.98 (3H, d, *J* = 6.6 Hz), 0.87 (3H, d, *J* = 6.4 Hz), 0.77 (3H, s); ^13^C-NMR (150 MHz, chloroform-*d*): δ 14.3, 17.4, 18.2, 19.5, 21.3, 22.5, 24.5, 26.5, 26.6, 27.9, 28.7, 31.1, 33.7, 33.8, 34.9, 38.7, 39.3, 39.5, 41.3, 42.3, 42.9, 47.6, 49.0, 50.6, 54.1, 58.7, 77.0, 70.8, 124.4, 143.5, 183.0; HRMS (ESI+): *m/z* 455.3563 [M + H]^+^.

3-Acetyl-*α*-boswellic acid (*α*-ABA, **12**): colorless needles; ^1^H-NMR (600 MHz, chloroform-*d*); δ 5.28 (1H, brt), 5.17 (1H, brt), 2.05 (3H, s, Ac), 1.16 (3H, s), 1.12 (3H, s), 0.97 (3H, s), 0.88 (3H, s), 0.84 (6H, s), 0.77 (3H, s); ^13^C-NMR (150 MHz, chloroform-*d*): δ 13.2, 16.8, 19.7, 21.3, 23.5, 23.6, 23.7, 25.9, 26.1, 27.0, 28.4, 29.7, 31.1, 32.5, 32.8, 33.3, 34.4, 34.7, 37.1, 37.4, 39.8, 41.9, 46.4, 46.8, 46.9, 50.5, 73.7, 121.9, 145.1, 170.3, 178.2; HRMS (ESI^‒^): *m/z* 497.3918 [M − H]^+^.

*α*-Boswellic acid (*α*-BA, **13**): white solid; ^1^H-NMR (600 MHz, chloroform-*d*); δ 5.16 (1H, br.t), 4.05 (1H, br.t), 1.31 (3H, s), 1.27 (3H, s), 1.12 (3H, s), 1.05 (3H, s), 0.94 (3H, s), 0.87 (6H, d, J = 3.0 Hz), 0.76 (3H, s); ^13^C-NMR (150 MHz, chloroform-*d*): δ 13.2, 16.7, 19.8, 23.6, 23.7, 24.1, 26.0, 26.0, 26.2, 27.0, 28.4, 29.7, 31.1, 32.5, 32.8, 33.3, 33.8, 34.7, 37.2, 37.6, 41.9, 46.8, 46.8, 47.2, 47.3, 49.0, 71.0, 121.8, 145.1, 179.5; HRMS (ESI^‒^): *m/z* 455.3748 [M − H]^+^.

*β*-Boswellic aldehyde (**14):** colorless solid; ^1^H-NMR (600 MHz, chloroform-*d*); δ 9.75 (1H, s), 5.14 (1H, br.t), 4.15 (1H, br.t), 1.27 (3H, s), 1.24 (3H, s), 1.10 (3H, s), 1.02 (3H, s), 0.97 (3H, s), 0.88 (6H, d, J = 6.6 Hz), 0.77 (3H, s); ^13^C-NMR (150 MHz, chloroform-*d*): δ 14.2, 17.0, 17.4, 17.8, 19.6, 21.3, 23.2, 23.5, 25.9, 26.5, 28.0, 28.7, 29.6, 31.2, 33.1, 33.2, 33.7, 37.2, 39.5, 39.6, 40.0, 41.5, 42.3, 46.3, 49.2, 52.2, 59.1, 69.3, 124.4, 139.6, 205.1; HRMS (ESI+): *m/z* 441.3726 [M + H]^+^.

*epi*-*α*-Amyrin (**15**)**:** white amorphous powder; ^1^H-NMR (600 MHz, chloroform-*d*): δ 5.11 (1H, t, *J* = 4.8 Hz), 3.38 (1H, Brs), 1.06 (3H, s), 0.98 (3H, s), 0.94 (6H, s), 0.89 (3H, d, 5.4 Hz), 0.84 (3H, s), 0.77 (3H, s), 0.76 (3H, d = J = 5.6 Hz); ^13^C-NMR (150 MHz, chloroform-*d*): δ 15.4, 16.8, 17.4, 18.2, 21.4, 22.3, 23.2, 23.3, 25.2, 26.5, 28.1, 28.2, 28.7, 31.2, 32.8, 33.2, 33.7, 36.9, 37.3, 39.6, 39.6, 40.1, 41.5, 42.1, 76.1, 124.4, 139.5; HRMS (ESI+): *m/z* 426.9669 [M + H]^+^.

*α*-Amyrin (**16**): colorless powder; ^1^H-NMR (600 MHz, chloroform-*d*); δ 5.11 (1H, t, *J* = 3.6 Hz), 3.21–3.18 (dd, *J* = 4.8 Hz), 1.05 (3H, s), 0.98 (3H, s), 0.97 (3H, s), 0.93 (3H, s), 0.88 (3H, s), 0.77 (3H, s), 0.76 (6H, d, 4.8 Hz); ^13^C-NMR (150 MHz, chloroform-*d*): δ 15.6, 15.6, 16.8, 17.4, 18.0, 18.3, 21.4, 23.2, 23.3, 26.6, 27.2, 27.9, 28.1, 28.7, 31.2, 32.9, 33.7, 36.8, 38.7, 39.6, 39.6, 40.0, 41.5, 42.0, 47.7, 55.1, 59.0, 79.0, 124.4, 139.5; HRMS (ESI+): *m/z* 426.9673 [M + H]^+^.

Tricosanoic acid (**17**): colorless solid; ^1^H-NMR (600 MHz, chloroform-*d*); δ 2.33 (2H, t, 7.8 Hz), 1.62 (4H, m), 1.60–1.08 (CH_2_)_1 8_, 0.94 (3H. m); ^13^C-NMR (150 MHz, chloroform-*d*): δ 14.1, 22.6, 24.7, 29.0, 29.23, 29.3, 29.4, 29.5, 31.9, 33.6, 177.8; HRMS (ESI+): *m/z* 355.2451 [M + H]^+^.

Incensole (**18**): colorless oil; ^1^H-NMR (600 MHz, chloroform-*d*); δ 5.08 (1H, t, *J* = 6.0, 5.4 Hz), 5.05 (1H, t, *J* = 7.2, 6.6 Hz), 3.27 (1H, d, *J* = 10.2 Hz), 1.61 (3H, s), 1.47 (3H, s), 1.03 (3H, s), 0.88–0.86 (6H, dd, J = 4.8, 1.8 Hz); ^13^C-NMR (150 MHz, chloroform-*d*): δ 16.12, 17.99, 18.05, 18.14, 20.66, 24.83, 30.62, 30.67, 32.31, 33.66, 34.83, 36.34, 38.60, 75.50, 84.15, 88.53, 121.77, 125.10, 134.17, 134.21; HRMS (ESI+): *m/z* 307.2623 [M + H]^+^.

Viridiflorol (**19**): colorless oil; ^1^H-NMR (600 MHz, chloroform-*d*); δ 1.17 (3H, s), 1.04 (3H, s), 1.02 (3H, s), 0.98 (3H, d, J = 7.2 Hz), 0.64 (1H, m), 0.14 (1H, t, *J* = 9.0 Hz); ^13^C-NMR (150 MHz, chloroform-*d*): δ 16.1, 16.3, 18.4, 18.8, 22.2, 25.7, 28.5, 28.6, 29.0, 32.1, 37.7, 38.4, 39.7, 58.2, 74.6; HRMS (ESI+): *m/z* 205.1950 [M − H_2_O + H]^+^.

Iso-serratol (**20**): colorless oil; ^1^H-NMR (600 MHz, chloroform-*d*): δ 5.07 (1H, t, *J* = 7.2, 6.6 Hz), 4.96 (1H, t, *J* = 6.6 Hz), 4.90 (1H, t, *J* = 6.6 Hz), 1.52 (9H, brt), 1.14 (6H, s); ^13^C-NMR (150 MHz, chloroform-*d*): δ 15.2, 15.5, 15.5, 23.9, 24.6, 27.4, 27.6, 28.2, 28.4, 37.7, 38.8, 39.3, 48.4, 73.9, 124.9, 125.7, 125.9, 132.9, 133.2, 134.0; HRMS (ESI+): *m/z* 489.2496 [M + H]^+^.

Lupenone (**21**): white amorphous powder; ^1^H-NMR (600 MHz, chloroform-*d*): δ 4.66 (1H, brs, H-29a), 4.54 (1H, brs, H-29b), 2.46 (1H, m), 2.37 (1H, m), 1.86 (2H, m), 1.68 (3H, s), 1.07 (6H, s), 1.02 (3H, s), 0.95 (3H, s), 0.93 (3H, s), 0.81 (3H, s); ^13^C-NMR (150 MHz, chloroform-*d*): δ 14.4, 15.9, 16.7, 18.0, 19.6, 19.7, 21.4, 21.4, 25.7, 26.6, 27.6, 29.7, 33.5, 34.4, 35.8, 36.6, 38.4, 39.4, 40.6, 40.7, 42.6, 43.0, 47.4, 48.0, 48.2, 49.8, 54.9, 109.3, 150.8, 217.7; HRMS (ESI+): *m/z* 447.3829 [M + Na]^+^.

### 2.5. In Vitro α-Glucosidase Inhibition

*α*-Glucosidase enzyme (E.C. 3.2.1.20) from *Saccharomyces cerevisiae* were purchased from Sigma-Aldrich (Darmstadt, Hesse, Germany) with product number of G0660-750UN and their inhibition assay was carried out [23] by using 0.1 M phosphate buffer (pH 6.8) solution at 37 °C. After enzyme (0.2 units/mL) incubation in phosphate buffered saline for 15 min with different concentrations of tested compounds, the *p*-nitrophenyl-*α*-D-glucopyranoside (substrate, 0.7 mM) was added and the variation in absorbance at 400 nm was observed for 30 min using a spectrophotometer (xMark™ Microplate Spectrophotometer, BIO-RAD, Hercules, CA, USA). For the kinetics studies, different concentrations (0.1, 0.2, 0.4 and 0.8 mM) of *p*-nitrophenyl-*α*-D-glucopyranoside (substrate) were used. In control the tested compounds were replaced with DMSO-*d*_6_ (7.5% final). Acarbose was used as the standard inhibitor. Three time the experiment was repeated having triplicate of each samples. The % inhibition was calculated by using the following formula:

% Inhibition = 100 − (OD test well/OD control) ×100 (OD = Optical density)

### 2.6. Computational Modeling and Molecular Docking

Molecular Operating Environment [24] was employed for the docking of four active compounds (**3**–**5** and **11**). Previously three-dimensional (3D) coordinates of *Saccharomyces cerevisiae α*-glucosidase enzyme was generated by homology modeling [25,26]. The primary sequence of *S. cerevisiae α*-glucosidase was retrieved from UniProtKB (AC#P53341). Homology modeling was carried out on Swiss Model server (https://swissmodel.expasy.org/) by using *S. cerevisiae* isomaltase (PDB code: 3A47, resolution: 1.59 Å and PDB code: 3AXH, resolution: 1.8 Å) as templates that has >72% identity with the target enzyme. The generated model comprises of 579 residues. The catalytic residues were identified by superimposing *S. cerevisiae* isomaltase structure (PDB code: 3AXH) in complex with isomaltose. The stereochemical properties of model were scrutinized by Procheck (http://services.mbi.ucla.edu/PROCHECK/), ERRAT (http://servicesn.mbi.ucla.edu/ERRAT/) and verify3D (http://servicesn.mbi.ucla.edu/Verify3D/). Procheck results showed that 444 (86.7%), 63 (12.3%), 3 (0.6%) and two (0.4%) residues lied in the most favored, additional allowed, generously allowed and disallowed regions, respectively. ERRAT showed 93.52 quality factor and Verify3D depicted that 95.5% residues showed average 3D-1D score of 0.7. The model is of good quality. The allosteric sites were identified by literature review [27,28,29,30] and MOE Site-Finder. Protonation state of protein was set according to the neutral pH, and partial charges were applied on protein by using AMBER12: EHT force field. Ten water molecules are involved in protein–substrate bridging in the active site; therefore, the coordinates of those water molecules were transferred in the model from template and retained during docking.

Human α-glucosidase (PDB ID: 5NN8) [31] structure was taken from Protein Data Bank; all the heteroatoms and water molecules were removed. Protonation state of protein was demonstrated according to the neutral pH. Protein was treated as described above.

The 3D-structures of the active compounds (**3**–**5** and **11**) were constructed on MOE, partial charges were applied on each structure and the structures were minimized with AMBER12: EHT force field until the gradient was reached to 0.1 kcal/mol/Å. Docking was carried out by Triangle matcher docking algorithm and London dG scoring function. The compounds were docked on the predicted allosteric sites to scrutinize their binding potential on different sites of *α*-glucosidase. On each site, thirty docked possess of compounds were saved for interaction analysis. After docking, protein–ligand interaction fingerprints (PLIF) were used to calculate the 2D-interactions of compounds with the binding sites.

## 3. Results and Discussion

### 3.1. Structural Elucidation of Compound **1**

**Figure 1:**
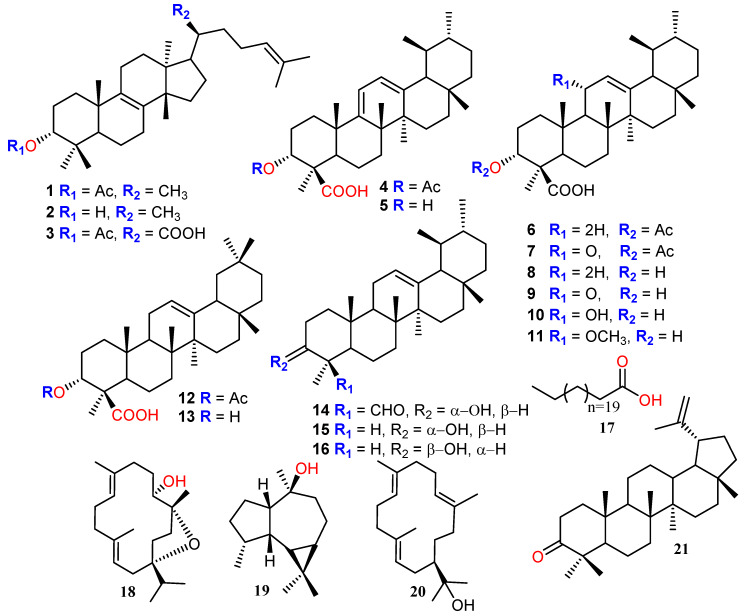
Structures of the compounds **1**–**21** isolated from *B. elongata.*

Compound **1** (Figure 1) was isolated as white amorphous powder having molecular formula of C_32_H_52_O_2_ which was further evidenced by HRMS (ESI^+^) which exhibited molecular ion peaks at *m/z m/z* 469.3292 [M + H]^+^ (calculated for C_32_H_53_O_2_: 469.3280); (7 degree of unsaturation). The Infrared spectrum of **1** showed characteristic absorption bands at 1724 and 1624 attributed to acetate (CH_3_CO) and double bond (C=C). The ^1^H-NMR spectrum of **1** showed seven tertiary methyls (δc 28.0, 25.7, 24.2, 20.1, 18.6, 16.6 and 15.3 each single), one secondary methyl (δ_H_ 0.93, d, *J* = 6.4 Hz; δc 18.6), one acetate methyl (δ_H_ 2.02, s) and a trisubstituted olefinic proton (δ_H_ 5.08, t = 7.2, 5.4 Hz, H-24), which are characteristic of tirucullane-type triterpene acetate [14,32]. The ^1^H NMR spectrum confirmed the presence of acetate group at C-3 and was in α-orientation as evidenced by the doublet of doublet (12.0, 4.8 Hz) of the β-oriented proton which appeared at δ_H_ 4.49, an interpretation and β-orientation further substantiated by heteronuclear multiple bond correlation (HMBC) correlation between H-5 (δ_H_ 1.62) and C-3 (δc 80.9) and nuclear overhauser effect spectroscopy (NEOSY) correlation between H-3 and CH_3_-23 position. On the other hand, the singlet peak at δ 5.08 (H-24) correlated with C-25 (δc 131.2), C-23 (δ 24.9), C-27 (δc 25.7) in the HMBC spectrum confirm the position of olefinic double bond between C-24 and C-25. NOESY correlations of Me-18 with H-20 further prove the configuration of C-20 to be S and thus the affiliation of the triterpene to the tirucallane series [33]. The stereochemistry of the compound is also in complete agreement with the published data [33,34] except acetate group at the C-3 position.

**Figure 2:**
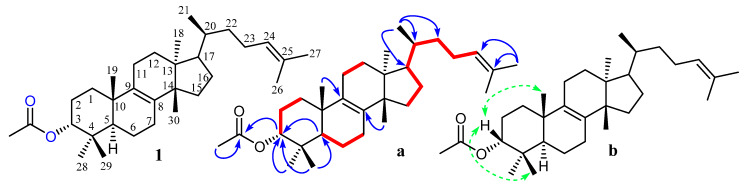
Structure of the compound **1**; (**a**) key heteronuclear multiple bond correlation HMBC (blue arrow) and H▬H COSY (red line) correlations**;** (**b**) key nuclear overhauser effect spectroscopy NOESY correlations of compound **1**.

The ^13^C-NMR spectrum of compound **1** displayed 32 peaks accounted for by nine methyls, five methines, ten methylenes and eight quaternary carbons. The ^13^C-NMR spectrum of **1** also attributed the presence of two olefinic groups at δ 133.9 and 133.6 (C-8 and C-9), 131.2 and 125.2 (C-24 and C-25) and one acetylated carbonyl group at δ 171.0 (C-32). All the positions of the substitutions were deduced using the COSY and HMBC techniques (Figure 2). ^1^H and ^13^C-NMR data were in complete agreement with those published [33,35,36]. Compound **1** was thus assigned the structure of 5α-tirucalla-8,24-dien-3α-acetate [37] obtained this compound by partial synthesis, while its hydroxyl analogs 5α-tirucalla-8,24-dien-3α-ol and 5α-tirucalla-7, 24-dien-3β-ol were previously published [33,38]. But, to the best knowledge, it has not been described as a natural product before.

The structures of the known compounds including 5α-tirucalla-8,24-dien-3α-ol (**2**) [33] 3α-acetoxytirucall-8,24-dien-21-oic acid (**3**) [32,39] 3-*O*-acetyl-9,11-dehydro-*β*-boswellic acid (**4**), 9,11-dehydro-β-boswellic acid (**5**) [32,40,41] 3-acetyl-*β*-boswellic acid (β-ABA, **6**), 3-acetyl 11-keto-β-boswellic acid (AKBA, **7**), *β*-boswellic acid (*β*-BA, **8**), 11-keto-*β*-boswellic acid (KBA, **9**)**,** 3*α*,11*α*-dihydroxyurs-12-en-24-oic acid (**10**) [11,41] 11*α*-methoxy-*β*-boswellic acid (**11**) [42] 3-acetyl-*α*-boswellic acid (*α*-ABA, **12**), *α*-boswellic acid (*α*-BA, **13**), *β*-boswellic aldehyde (**14),** *epi*-*α*-amyrin (**15**)**,** *α*-amyrin (**16**) [43] tricosanoic acid (**17**) [44], incensole (**18**) [32,45], viridiflorol (**19**) [46], iso-serratol (**20**) [47,48] and lupenone (**21**) [49,50] were determined on the basis of spectroscopic techniques and by comparison with the published data (Figure 1).

### 3.2. α-Glucosidase Inhibition and Structural-Activity Relationship (SAR)

All the isolated compounds **1**–**21** were screened for α-glucosidase enzyme inhibition at 1.0 mM concentration (Table 1). In the preliminary screening, four compounds (**3**–**5** and **11**) demonstrated significant in vitro α-glucosidase inhibitory properties with IC_50_ values in the range of 9.9 ± 0.48–56.8 ± 1.30 μM, while compounds **1**, **2**, **19** and **20** displayed % inhibition less than 50, therefore, were not evaluated for IC_50_. The remaining isolated compounds belonging to different classes including diterpenoids, triterpenoids and boswellic acids were already reported by our group with SAR study [11].

Comparing boswellic acids, compound **4** (9.9 ± 0.48 µM) showed potent inhibition followed by **11** (14.9 ± 1.31 µM) and **5** (20.9 ± 0.05 µM). Compound **4** exhibited highest inhibition against α-glucosidase enzyme compared all types of other boswellic acids reported in the literature until now [11,51]**.** Higher inhibition of **4** compared to **5** (both have same basic structure) may be due to the replacement of −OH with acetyl group resulted in the increase of α-glucosidase activity**.** The compound **4** was found to be 94 times more active than the clinically standard inhibitor acarbose (IC_50_ = 942 ± 0.74 μM). The previous investigation showed that α-ABA, β-ABA and AKBA were the most promising glucosidase inhibitors having acetyl group at C-3 position.

Compound **3**, carrying COOH group, exhibited higher activity (IC_50_ = 56.8 ± 1.30 μM) than **1** (inactive) having methyl group at C-20, indicating that the higher activity of compound **3** may be due the replacement of methyl group with carboxylic acid. Similarly, comparing compound **11** with **10**, the higher activity of **11** may be due to the replacement of hydroxyl group with -OCH_3_ at C-11 position, while the remaining skeleton of both compounds is same. From SAR perspective, among all samples tested for the inhibition of α-glucosidase, we conclude that the presence of acetyl group at the C-3α position and carboxylic acid at C-24 position in the ursane type boswellic acids is essential.

### 3.3. Kinetics Studies

**Figure 3:**
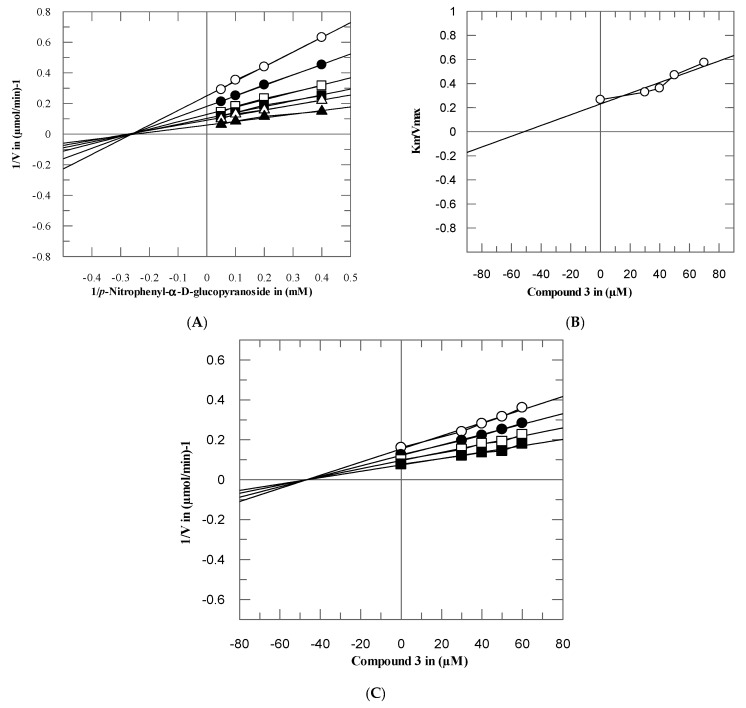
The inhibition of α-glucosidase by compound **3** (**A**) Lineweaver–Burk plot of reciprocal of rate of reaction (velocities) vs. reciprocal of substrate (*p*-nitrophenyl-*α*-D-glucopyranoside) in the absence (▲), and in presence of 30 (∆), 40 (■), 50 (□), 60 (●), and 70 µM (○) of compound **3**. (**B**) Secondary replot of Lineweaver–Burk plot between the slopes of each line on Lineweaver–Burk plot vs. different concentrations of compound **3**. (**C**) Dixon plot of reciprocal of rate of reaction (velocities) vs. different concentrations of compound **3**.

**Figure 4:**
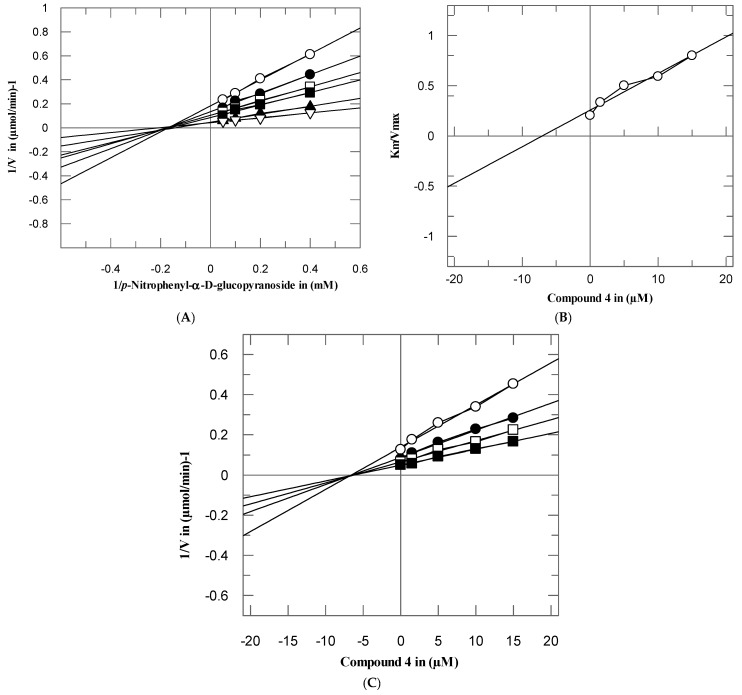
The inhibition of α-glucosidase by compound **4** (**A**) Lineweaver–Burk plot of reciprocal of rate of reaction (velocities) vs. reciprocal of substrate (*p*-nitrophenyl-*α*-D-glucopyranoside) in the absence (∆), and in presence of 2.5 (▲), 5 (■), 10 (□), 15 (●), and 20 µM (○) of compound **4**. (**B**) Secondary replot of Lineweaver–Burk plot between the slopes of each line on Lineweaver–Burk plot vs. different concentrations of compound **4**. (**C**) Dixon plot of reciprocal of rate of reaction (velocities) vs. different concentrations of compound **4**.

**Figure 5:**
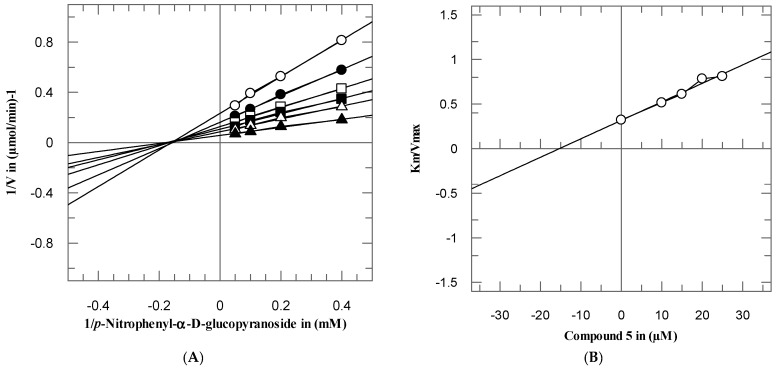

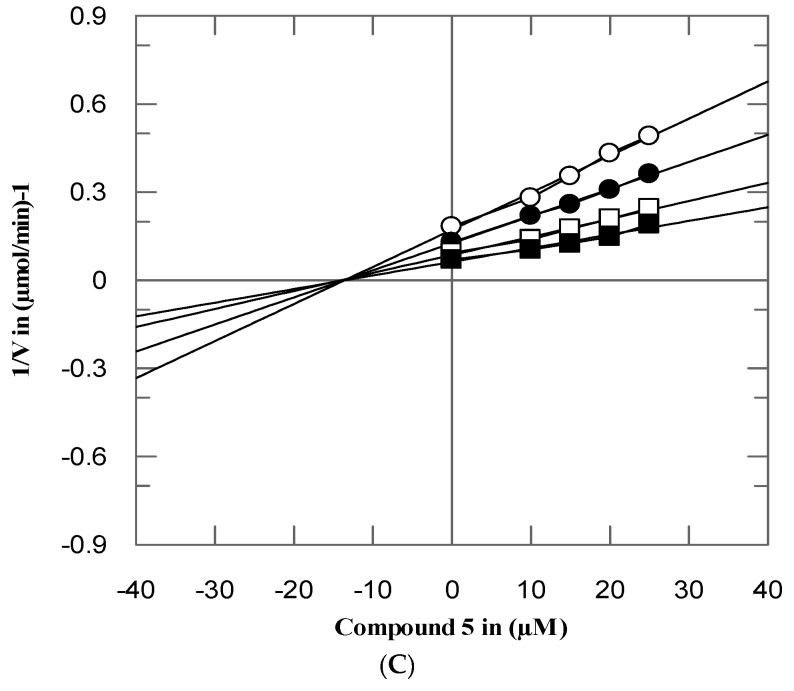
The inhibition of α-glucosidase by compound **5** (**A**) Lineweaver–Burk plot of reciprocal of rate of reaction (velocities) vs. reciprocal of substrate (*p*-nitrophenyl-*α*-D-glucopyranoside) in the absence (▲), and in presence of 10 (∆), 15 (■), 20 (□), 20 (●), and 25 µM (○) of compound **5**. (**B**) Secondary replot of Lineweaver–Burk plot between the slopes of each line on Lineweaver–Burk plot vs. different concentrations of compound **5**. (**C**) Dixon plot of reciprocal of rate of reaction (velocities) vs. different concentrations of compound **5**.

**Figure 6:**
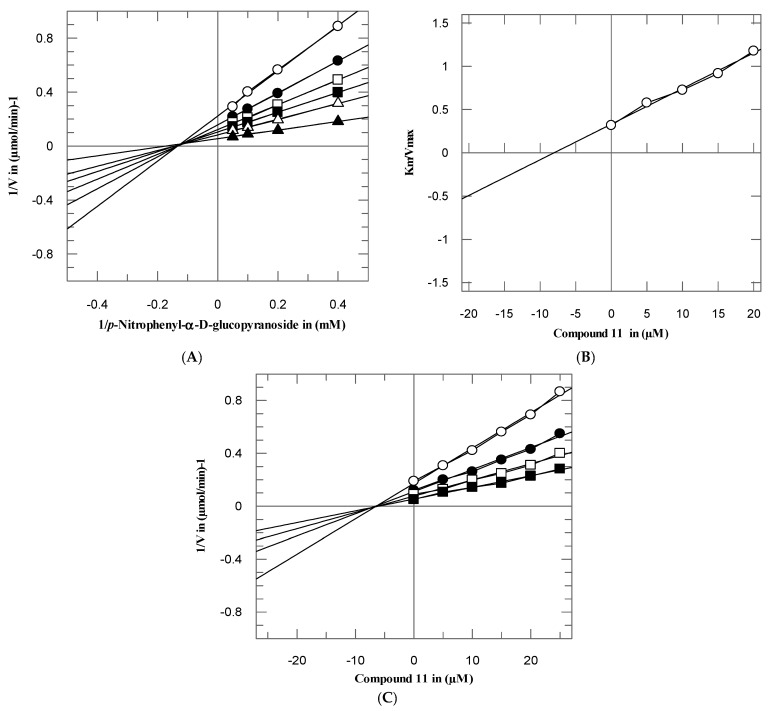
The inhibition of α-glucosidase by compound **11** (**A**) Lineweaver–Burk plot of reciprocal of rate of reaction (velocities) vs. reciprocal of substrate (*p*-nitrophenyl-*α*-D-glucopyranoside) in the absence (▲), and in presence of 5 (∆), 10 (■), 15 (□), 20 (●), and 25 µM (○) of compound **11**. (**B**) Secondary replot of Lineweaver–Burk plot between the slopes of each line on Lineweaver–Burk plot vs. different concentrations of compound **11**. (**C**) Dixon plot of reciprocal of rate of reaction (velocities) vs. different concentrations of compound **11**.

To investigate the mode of interaction and dissociation constant of these potent compounds, the kinetics studies on active compounds **3**–**5** and **11** were performed, with different concentrations of test compounds and substrates. These compounds inhibited the α-glucosidase enzyme in a concentration-dependent manner with *Ki* values were between 7.05 ± 0.75–51.15 ± 0.63 µM. From the kinetics studies, it was deduced that the compounds **3**–**5** and **11** are non-competitive inhibitors with *K_i_* values in range 77.05 ± 0.75–51.15 ± 0.63 µM. The type of inhibition was determined by Lineweaver–Burk plots, the reciprocal of the rate of the reaction was plotted against the reciprocal of substrate concentrations to monitor the effect of inhibitor on both *K_m_* and *V_max_*. It was observed from Lineweaver–Burk plots that all compounds **3**–**5** and **11** clearly showed non-competitive inhibition Figure 3, Figure 4, Figure 5 and Figure 6A. In non-competitive inhibition, the *V_max_* of enzyme decreased, while *K_m_* are not affected. The Lineweaver–Burk plots (section A) of all Figure 3, Figure 4, Figure 5 and Figure 6 showed that in the presence of compounds **3**–**5** and **11** the *V_max_* of α-glucosidase enzyme decreased significantly, while the *K_m_* remain constant, which indicated the mixed-type of inhibition. The secondary replots of Lineweaver–Burk plots were used to determine the *K_i_* values. The *Ki* values were calculated by plotting the slope of each line in the Lineweaver–Burk plots against different concentrations of compounds **3**–**5** and **11** (Figure 3, Figure 4, Figure 5 and Figure 6B). The *K_i_* value was confirmed from Dixon plot by plotting the reciprocal of the rate of reaction against different concentrations of compounds **3**–**5** and **11** (Figure 3, Figure 4, Figure 5 and Figure 6C).

### 3.4. Molecular Docking of α-Glucosidase Inhibitors

**Figure 7:**
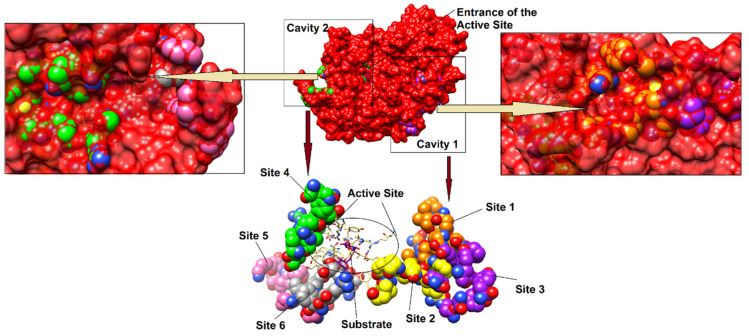
Structural topology of *S. cerevisiae* α-glucosidase is shown. The generated cavities are displayed in boxes. The predicted allosteric sites are shown in sphere model, each site is labeled. The active site residues (shown in yellow sticks) are presented in complex with the substrate molecule (isomaltose, shown in purple sticks).

The isolated compounds (**3**–**5** and **11**) exhibited significant non-competitive inhibition of α-glucosidase in vitro. These triterpenic acids are involved in the allosteric modulation of α-glucosidase. Therefore, we identified several hotspots as allosteric sites (Table 2) of α-glucosidase and performed molecular docking to predict the mode of binding of compounds in the predicted allosteric sites of *S.* *cerevisiae* α-glucosidase. Previously 3D-coordinates of *S. cerevisiae* α-glucosidase were generated by homology modeling to be used in molecular docking studies. The active site of the enzyme comprises of a catalytic triad (Asp214, Glu276 and Asp349) where Asp214 work as nucleophile, Glu276 act as a proton donor for substrate, and the transition state of substrate is stabilized by Asp349. Additionally, several residues (Asp68, Tyr71, Val108, His111, Phe157, Phe158, Phe177, Gln181, Arg212, Thr215, Leu218, Glu276, Ala278, Phe300, Arg312, His348, Asp349, Gln350, Asp408, Arg439 and Arg443) creates the lining of active site and provide strong hydrophilic and hydrophobic interactions to the substrate molecule. These residues also stabilize the inhibitor acarbose. In the active site of α-glucosidase many water molecules (Wat1021, Wat1026, Wat1056, Wat1058, Wat1061, Wat1087, Wat1102, Wat1122, Wat1174 and Wat1228) are involved in enzyme-substrate and enzyme-inhibitor bridging. The rim of the active site gorge is surrounded by the gate keeping residues (Phe231, His239, Asn241, His279, Glu304, Arg312) that regulate the entry and exit of ligand in the active site. The enzyme substrate complex is shown in Figure 7.

In order to determine the non-competitive behavior of compounds **3**–**5** and **11**, different allosteric sites were recognized by literature review that reveals six potential hotspots are present as allosteric sites in *S.* α-glucosidase enzyme (Table 2). [27] revealed that two non-competitive inhibitors (oleanolic acid and ursolic acid) binds at two different sites to induce allosteric regulation. It was shown that oleanolic acid binds at allosteric site (**AS**)-1 which is created by Trp14, Lys12, Ser295, Ala289, His258, Tyr292, Lys262, Val265, Ile271 and Glu270 while ursolic acid binds at **AS-4** (Gln66, Gln67, Met69, Ser179, Arg180, Glu405, Val407, Lys410, Asn411, Trp465) [27] demonstrated that a mixed type inhibitor ((E)-3-butylideneisobenzofuran-1(3H)-one) binds to a site close to the catalytic site and is formed by residues Thr287, Val297, Ser299, His302, Ile334, Trp340, Ala341, Thr342 and Tyr344. This site was considered as **AS-2** in the current docking studies. Moreover, **AS-1, AS-3, AS-5** and **AS-6** were identified by [30] as the binding site for some xanthone derivatives that exhibited non-competitive inhibition of α-glucosidase. **AS-1** to **AS**-**3** is located away from the active site, while **AS-4** to **AS-6** is situated near the active site. The compounds **3**–**5** and **11** were targeted at all the sites (**AS-1** to **AS-6**) individually and the docked conformation of each molecule with the high negative docking score was considered as the most optimal binding orientation and selected for binding mode analysis. The optimal conformations of **3**–**5** and **11** were well accommodated inside **AS-1** and **AS-4** where compound **4** exhibited highest binding potential (>−10), followed by compounds **11** (->9), **5** (−9) and **3** (>−8). The docking scores of compounds at **AS-2,** were in range of −4 to >−6, suggesting that this may not be an appropriate binding site for our compounds (supporting information, Table S1). Similarly, compounds did not possess good binding potential for **AS-3** and exhibited docking scores in range of >−6 to >−7 and the docked conformations of compounds were surface exposed that did not show favorable binding interactions. However, compounds demonstrated good binding potential for **AS-5** (docking scores in range of >−8 to >−7) compared to **AS-2** and **AS-3**, however lower score than **AS-1**. When docked at **AS-6**, all the compounds remained surface exposed therefore displayed least binding potential (>−6 to >−7), thus it was considered as the most unappropriated binding site for these triterpenic acids. The docking results are summarized in Table S1, in supporting information.

**Figure 8:**
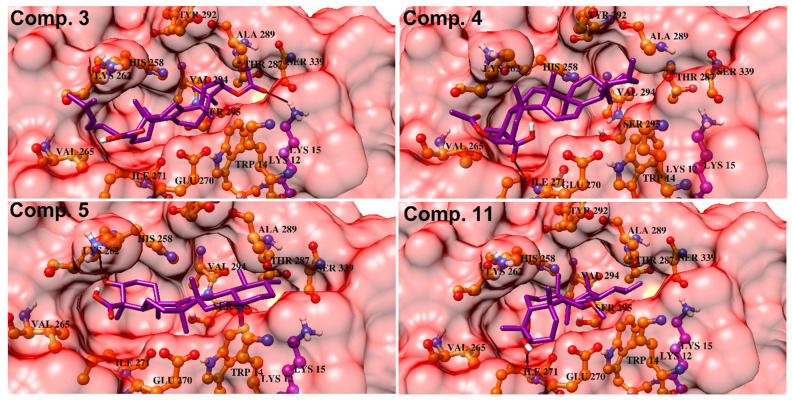
Docked orientations of compounds **3**–**5** and **11** are shown in the Cavity 1. The compounds are shown in purple sticks, hydrogen bonds are presented in black lines.

**AS-1** to **AS-3** are located opposite to the active site, we selected **AS-1** to **AS-3** communally (named as **Cavity 1**) and docked the compounds at this cavity. Based on docking scores and binding interactions, the compounds depicted significantly higher binding potential at this cavity. The most active compound (**4**) showed -10.77 docking score, followed by compounds **11** (−10.69), **5** (−10.21) and **3** (−9.83). Additionally, **Cavity 2** was created by combining **AS-4** to **AS-6** (Gln66, Gln67, Met69, Tyr142, Lys147, Ile149, Pro150, Lys155, Phe157, Leu176, Ser179, Arg180, Asp227, Asp232, Ile236, Leu237, Gln238, Gly243, Ser244, Phe311, Arg312, Glu405, Val407, Lys410, Asn411, Trp465). The compounds exhibited >−10 to >−8 docking score at cavity 2. The docking scores indicate that compounds have higher binding potential for cavity 1 than cavity 2. The binding orientation also showed that compounds are well accommodated at the groove present in the cavity 1. All the predicted sites, and cavities **1** and **2** are shown in Figure 7. The compounds **4**, **5** and **11** are lodged at **AS-1** in the cavity 1 and stabilized by hydrogen bonding with Ile271 and His258. The carboxylic group of **4** and **11** accepts H-bond from the amino nitrogen of Ile271, while the -OH of compound **5** donates H-bond to the carbonyl oxygen of His258. The docking mode of **3** depict that compound is located at **AS-1**, however the acetate group of **3** interact with the side chain of Lys15 of **AS-3**. The docking scores of compounds at cavity 1 and their binding interactions are tabulated in Table 3. The docked conformations of compounds in cavity 1 are presented in Figure 8. The docking score are well correlated with the in vitro experimental findings.

**Figure 9:**
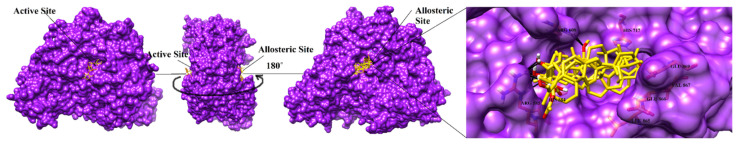
Three-dimensional structure of Human α-glucosidase. The active site and the allosteric binding sites are also demonstrated. The binding mode of acarbose (yellow stick model) is shown in the active site of human α-glucosidase. The binding mode of compounds **3**–**5** and **11** (shown in yellow stick model) in the allosteric site of Human α-glucosidase is highlighted. The interacting residues are shown in orange sticks while hydrogen bonds are displayed in black lines.

Additionally, compounds **3**–**5** and **11** were docked at the allosteric binding site of Human α-glucosidase [31,52]. The compounds showed excellent binding affinities and interactions. The acetic and the enoic acid moieties of compound **3** mediate H-bonding with the side chains of Glu896 and Arg585. Similarly, the carboxylic acid moiety of Compound 4 mediated bidentate interactions with the side chain of Arg585. The -OH moiety of compound 5 interact with the side chain of Arg585. Moreover, His584 provide H-π interaction to the compound. The -OH and the methoxy groups of compound 11 interact with the side chains of Arg585 and Arg608, respectively. The docking interactions suggest that the compound has binding potential with the human α-glucosidase as well. This is also confirmed by the docking score (Table 3). The binding mode of compounds is shown in Figure 9.

Moreover, absorption, distribution, metabolism, excretion and toxicity (ADMET) of compounds were scrutinized by admetSAR (http://lmmd.ecust.edu.cn/admetsar2/). The results indicate that compounds are non-mutagenic and non-carcinogenic. Caco-2 cells are a human colon epithelial cancer cell line used as a model of human intestinal absorption of drugs and other compounds. It was observed that compounds **3, 4** and **11** are Caco-2 negative means impermeable, while compound **4** is Caco-2 positive, suggesting that **4** is permeable to human intestinal cell. Moreover, compounds **2**, **5** and **11** and non-permeable to blood brain barrier, and none of the compound displayed cytochrome inhibitory promiscuity. The gastrointestinal absorption of compounds **3**–**5** is low, while **11** has high GI absorption, thus it is demonstrated that compound **11** would be as excellent drug like molecule because of its tendency to pass through GIT. All the compounds (**3**–**5** and **11**) are not substrate of P-glycoprotein, however, compounds **3** and **4** may serve as inhibitor of P-glycoprotein. The calculated acute oral toxicity of compounds **3**–**5** and **11** are 1.641, 2.381, 2.469 and 3.201 kg/mol, respectively. It indicates that compounds **3** and **4** belong to category III, while compounds **5** and **11** are from category I. The category I comprises of compounds with LD_50_ values ≤ 50 mg/kg, category II possesses compounds with LD_50_ values ≥ 50 mg/kg but ≤ 500 mg/kg, category III includes compounds with LD_50_ values ≥ 500 mg/kg but ≤ 5000 mg/kg and category IV consisted of compounds with LD_50_ values ≥ 5000 mg/kg. The results showed that compounds **3** and **4** are safer that compounds **5** and **11**. The Human oral bioavailability Score of all the compounds is 0.56, indicating moderate bioavailability. The results are tabularized in Table 4.

## 4. Conclusions

One new triterpene **1** together with twenty known compounds (**2**–**21**) were isolated, first time, from the methanolic extract of the oleo-gum resin of *B. elongata*. Eight compounds (**1**–**5**, **11**, **19** and **20**) were further screened for in vitro α-glucosidase inhibitory activity. Compounds **3**–**5** and **11** showed significant activity against α-glucosidase with IC_50_ values ranging from 9.9–56.8 μM. Structure-activity-relationship studies revealed that the carboxylic group plays a crucial role among all. Kinetics studies on the active compounds **3**–**5** and **11** were carried out to investigate their mechanism (mode of inhibition and dissociation constants *K_i_*). All compounds were found to be non-competitive inhibitors with *K_i_* values in the range of 7.05 ± 0.17‒51.15 ± 0.25 µM. Moreover, in silico docking study was performed to see the allosteric hotspot for ligand binding which is targeted by our active compounds investigates the binding mode of active compounds and it was identified that compounds preferentially bind in the allosteric binding sites of α-glucosidase. The results obtained from docking study suggested that carboxylic group is responsible for their biologic activities. To the best knowledge, this is the first report on the phytochemical investigation of *B. elongata*. In addition, the α-glucosidase inhibition potential of all the active compounds is reported here for the first time.

## Figures and Tables

**Table 1:** α-Glucosidase inhibition of active constituents from *B. elongata*.

Code	IC_50_ = µM ± SEM	K*i* = µM ± SEM	Type of Inhibition
**1**	NA	ND	ND
**2**	NA	ND	ND
**3**	56.8 ± 1.30	51.15 ± 0.63	Non-competitive
**4**	9.9 ± 0.48	7.05 ± 0.75	Non-competitive
**5**	20.9 ± 0.05	15.30 ± 0.54	Non-competitive
**11**	14.9 ± 1.31	8.05 ± 0.38	Non-competitive
**19**	NA	ND	ND
**20**	NA	ND	ND
Acarbose	942 ± 0.74	-	-

NA = Not active; SEM = Standard error Mean; ND = Not determined.

**Table 2:** Predicted allosteric sites of *S. cerevisiae α*-glucosidase enzyme.

Predicted Allosteric Sites	Binding Residues	References
1	Lys12, Trp14, His258, Lys262, Val265, Glu270, Ile271, Thr287, Ala289, Tyr292, Val294, Thr295, Ser339	[27,28]
2	Thr287, Val297, Ser299, His302, Ile334, Trp340, Ala341, Thr342, Tyr344	[29]
3	Thr9, Pro11, Lys15, Ile334, Asn335, Ser339, Trp340, Thr380, Tyr508, Tyr510, Tyr529	[28]
4	Gln66, Gln67, Met69, Ser179, Arg180, Glu405, Val407, Lys410, Asn411, Trp465	[27]
5	Tyr142, Ile149, Lys147, Pro150, Asp227, Asp232, Ile236	[28]
6	Lys155, Phe157, Leu176, Leu237, Gln238, Gly243, Ser244, Phe311, **Arg312**	[28]

**Table 3:** Docking scores and binding interactions of compounds **3**–**5** and **11** at Cavities **1** and **2** of *S. Cerevisiae* α-Glucosidase and Human α-Glucosidase.

	*Saccharomyces Cerevisiae* α-Glucosidase				
**Compounds**	**Cavity 1**				
	**Scores**	**Binding Interactions**			
		**Ligand**	**Receptor**	**Interaction**	**Distance (Å)**
**3**	−9.83	O77	NZ-LYS15	HBA	3.21
**4**	−10.77	O75	N-ILE271	HBA	3.00
**5**	−10.21	O70	O-HIS258	HBA	3.26
**11**	−10.69	O78	N-ILE271	HBA	2.85
	**Cavity 2**				
**Compounds**	**Scores**	**Binding Interactions**			
		**Ligand**	**Receptor**	**Interaction**	**Distance (Å)**
**3**	−8.71	O84	NZ-LYS418	HBA	2.72
**4**	−10.73	O79	N-SER179	HBA	3.01
**5**	−9.60	O77	ND2-ASN411	HBA	2.58
**11**	−9.98	O81	N-SER179	HBA	2.97
**Human α-Glucosidase**					
		**Binding Interactions**			
**Compounds**	**Scores**	**Ligand**	**Receptor**	**Interaction**	**Distance (Å)**
**3–17**	−9.02	O77	N-GLU869	HBA	2.78
		O84	NE-ARG585	HBA	3.39
**4–39**	−10.59	O75	NE-ARG585	HBA	2.89
		O75	NH2-ARG585	HBA	3.32
**5–63**	−9.83	O70	NH2-ARG585	HBA	1.93
		C58	5-ring-HIS584	H-π	3.99
**11–92**	−10.07	O71	NH2-ARG585	HBA	2.99
		O81	NH2-ARG608	HBA	3.19

HBA = Hydrogen Bond Acceptor.

**Table 4:** Absorption, distribution, metabolism, excretion and toxicity (ADEMT) properties of Compounds **3**–**5** and **11.**

S#	Properties	Compounds			
		3	4	5	11
1	Ames mutagenesis	-	-	-	-
2	Acute Oral Toxicity	III	III	I	I
3	Blood Brain Barrier	-	+	-	-
4	Caco-2	-	-	+	-
5	Carcinogenicity	-	-	-	-
6	CYP1A2 inhibition	-	+	-	-
7	CYP2C19 inhibition	-	-	-	-
8	CYP2C9 inhibition	-	-	-	-
9	CYP2C9 substrate	-	-	-	-
10	CYP2D6 inhibition	-	-	-	-
11	CYP2D6 substrate	-	-	-	-
12	CYP3A4 inhibition	-	-	-	-
13	CYP3A4 substrate	+	+	+	+
14	CYP inhibitory promiscuity	-	-	-	-
15	Human Intestinal Absorption	low	low	low	high
16	Human oral bioavailability Score	0.56	0.56	0.56	0.56
17	Acute Oral Toxicity	1.641 kg/mol	2.381 kg/mol	2.469 kg/mol	3.201 kg/mol
18	P-glycoprotein inhibitor	+	+	-	-
19	P-glycoprotein substrate	-	-	-	-
20	Water solubility	−4.92347	−4.67124	−3.78695	−3.90081

## Supplementary Materials

The following are available online at https://www.mdpi.com/2218-273X/10/5/751/s1, NMR and MS data for compounds **1**–**21** is included. This material is available free of charge via MDPI website. Table S1: Docking scores and binding interactions of compounds **3**-**5** and **11** on the predicted allosteric sites.
