# Supplementary material for: Triterpenic Acids as Non-Competitive α-Glucosidase Inhibitors from Boswellia elongata with Structure-Activity Relationship: In Vitro and In Silico Studies
Source: Biomolecules. 2020 May 12;10(5):751. doi: 10.3390/biom10050751 (PMC7278020; doi:10.3390/biom10050751)
Supplement: Supplementary file 1 [file biomolecules-10-00751-s001.pdf]

SUPPLEMENTARY MATERIALS

α-Glucosidase inhibitory activity of triterpenic acids from *Boswellia elongata* with structure-activity relationship: In vitro and in silico studies

Najeeb Ur Rehman[a], Mohammed Al-Azri[a], Ajmal Khan[a], Sobia Ahsan Halim[a], Zahra Al-Abri[a], Ahmed Al-Rawahi[a], Rene Csuk[b] and Ahmed Al-Harrasi[a]*

[a]*Natural & Medical Sciences Research Center, University of Nizwa, P.O Box 33, Postal Code 616, Birkat Al Mauz, Nizwa, Sultanate of Oman*

[b]*Martin-Luther-University Halle-Wittenberg, Organic Chemistry, Kurt-Mothes-Str. 2, D-06120 Halle (Saale) Germany*

***Corresponding author (A. Al-Harrasi)**

Natural & Medical Sciences Research Center, University of Nizwa, P.O Box 33, Postal Code 616, Birkat Al Mauz, Nizwa, Sultanate of Oman (phone: +96825446328; e-mail: aharrasi@unizwa.edu.om

Table S1. Docking scores and binding interactions of Compounds 3-5 and 11 on the predicted allosteric sites

Sites	Compounds							
	3		4		5		11	
	Score	Interactions	Score	Interactions	Score	Interactions	Score	Interactions
		Lig. Rec. Interaction Dis.		Lig. Rec. Interaction Dis.		Lig. Rec. Interaction Dis.		Lig. Rec. Interaction Dis.
**AS-1**	-8.54	O77 NZ-LYS262 HBA 2.92	-10.8	O79 NZ-LYS15 HBA 3.23	-9.58	O70 O-ILE271 HBD 2.70	-9.99	O79 OE1-GLU10 HBD 2.67
**AS_2**	-5.18	O84 ND2-ASN335 HBA 2.66	-6.71	O76 O-LEU332 HBD 3.12	-4.68	O78 O-ASN335 HBD 3.07	-6.67	O79 O-LEU332 HBD 3.00
**AS-3**	-6.91	O84 ND2-ASN335 HBA 2.47	-7.05	O75 NE2-GLN HBA 2.58	-7.23	O70 NE2-GLN513 HBA 3.07	-7.88	SE
**AS-4**	-8.5	O84 N-ARG180 HBA 3.03	-10.57	O76 OG-SER179 HBD 3.08<br>O79 N-SER179 HBA 3.43	-9.38	O70 OE1-GLU414 HBD 2.98	-9.27	O78 NE-ARG180 HBA 3.22
**AS-5**	-7.93	O77 NZ-LYS 236 HBA 2.65	-8.30	O75 NZ-LYS418 HBA 2.95	-8.24	O70 OG-SER235 HBD 2.64<br>O77 N-LYS236 HBA 2.31	-8.34	O78 NZ-LYS418 HBA 3.22
**AS-6**	-6.84	SE	-7.32	SE	-7.74	SE	-7.93	SE
**Cav 1**	-9.83	O77 NZ-LYS15 HBA 3.21	-10.77	O75 N-ILE271 HBA 3.00	-10.21	O70 O-HIS258 HBD 3.26	-10.69	O78 N-ILE271 HBA 2.85
**Cav 2**	-8.71	O84 NZ-LYS418 HBA 2.72	-10.73	O79 N-SER179 HBA 3.01	-9.60	O77 ND2-ASN411 HBA 2.58	-9.98	O81 N-SER179 HBA 2.97

Lig = ligand, Rec = receptor, Dis = distance, Cav = cavity, HBA= Hydrogen bond acceptor, HBD = Hydrogen bond donor, SE = Surface Exposed

Representative NMR and HRMS spectra of the isolated compounds

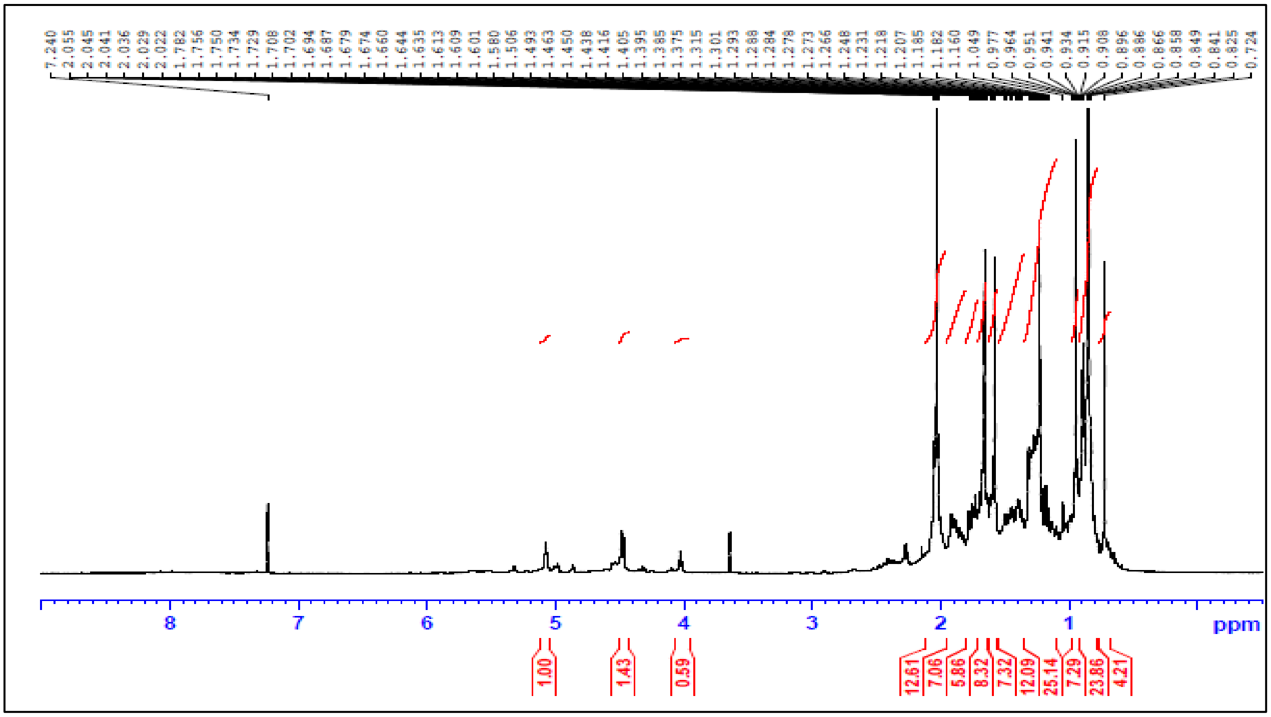

Figure S1. ^{1}H-NMR (600 MHz, $CDCl_3$) of compound **1**

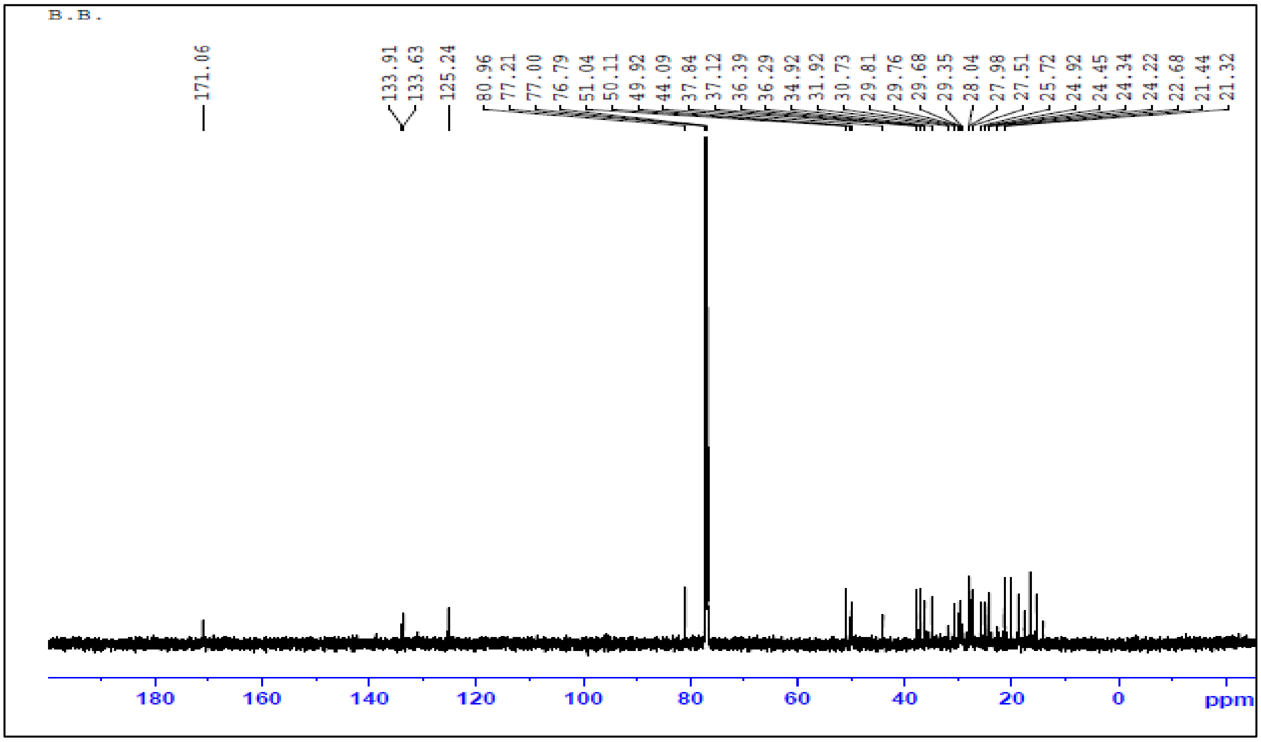

Figure S2. ^{13}C-NMR (150 MHz, $CDCl_3$) of compound **1**

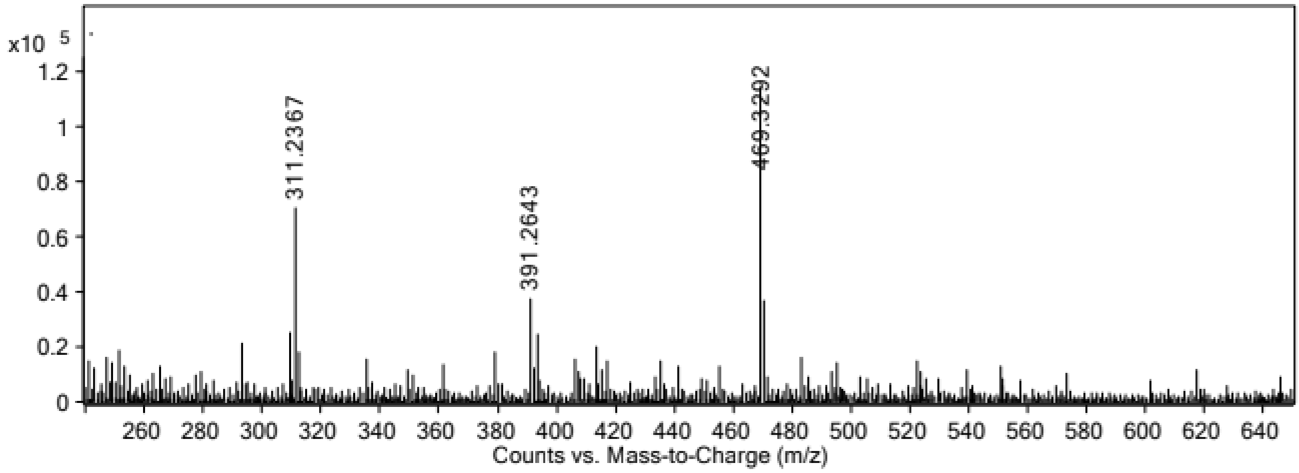

Figure S3. HRMS (ESI^+) of compound **1**

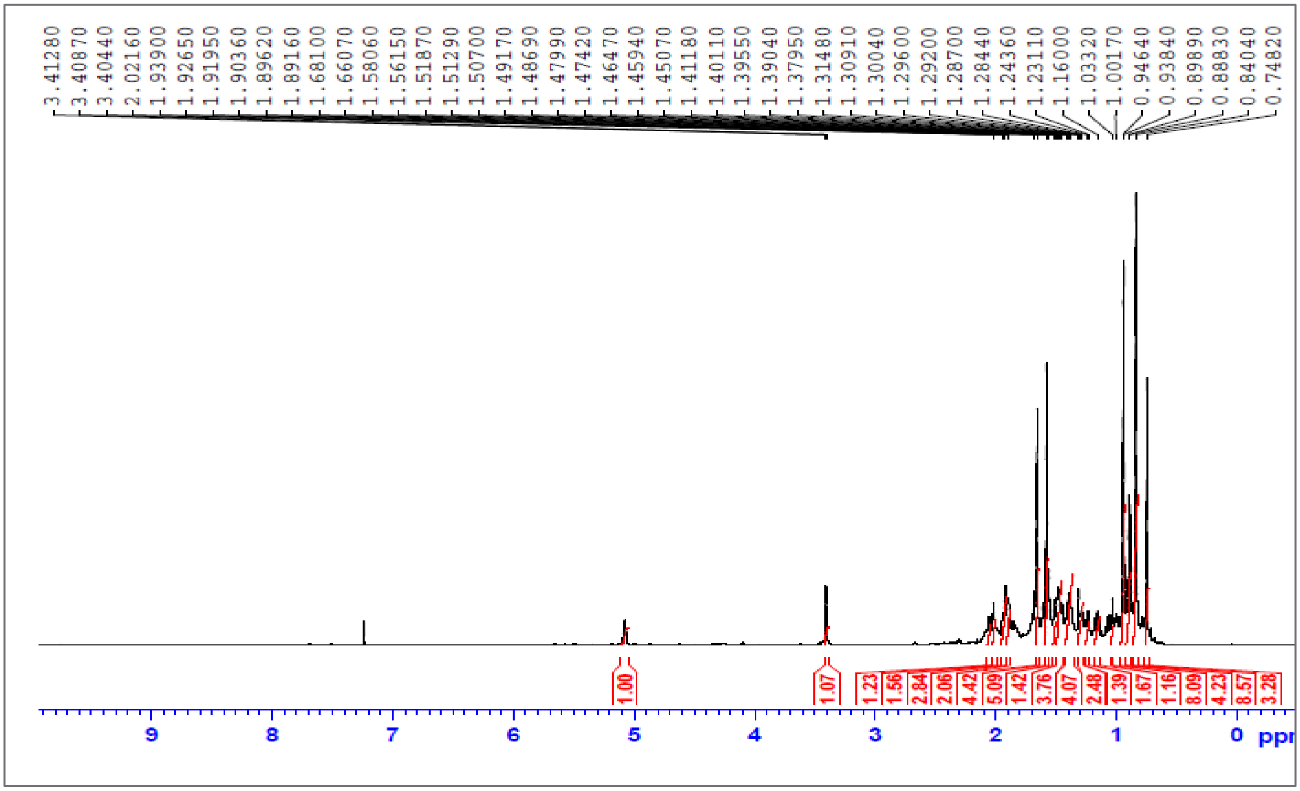

Figure S4. ^{1}H-NMR (600 MHz, $CDCl_3$) of compound **2**

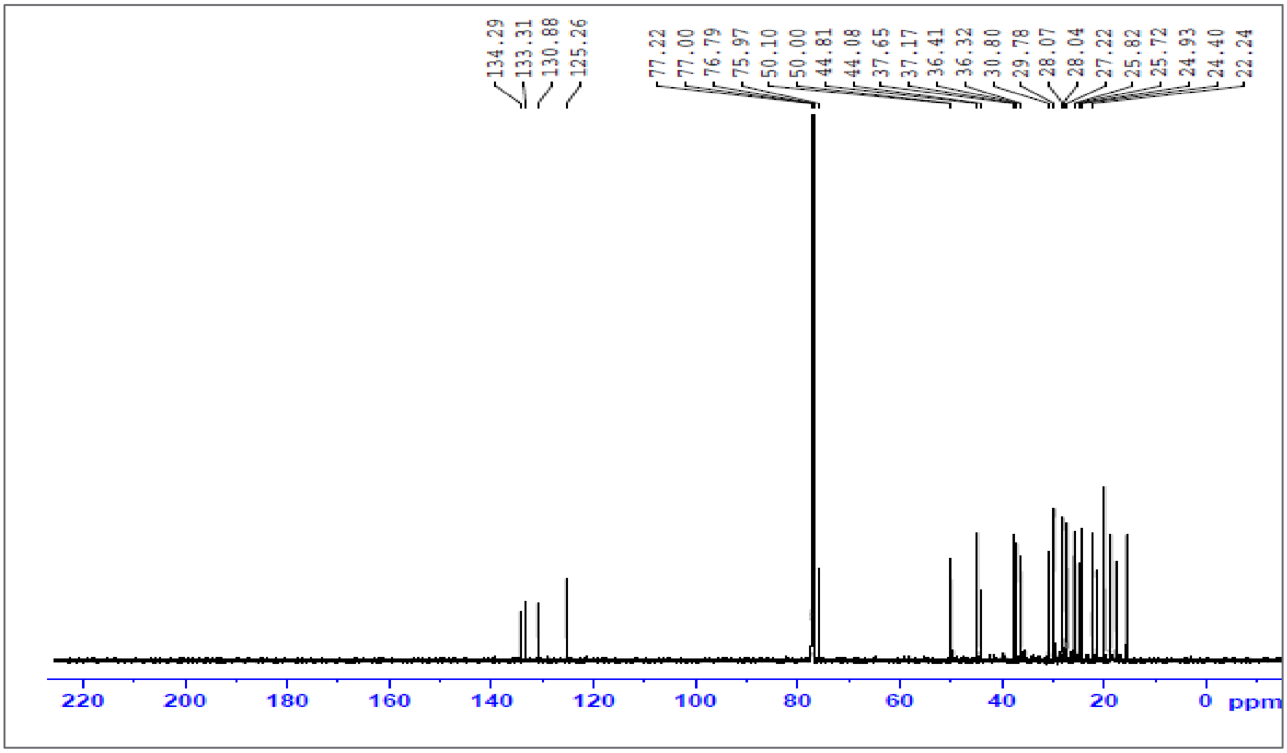

Figure S5. ^{13}C-NMR (150 MHz, $CDCl_3$) of compound **2**

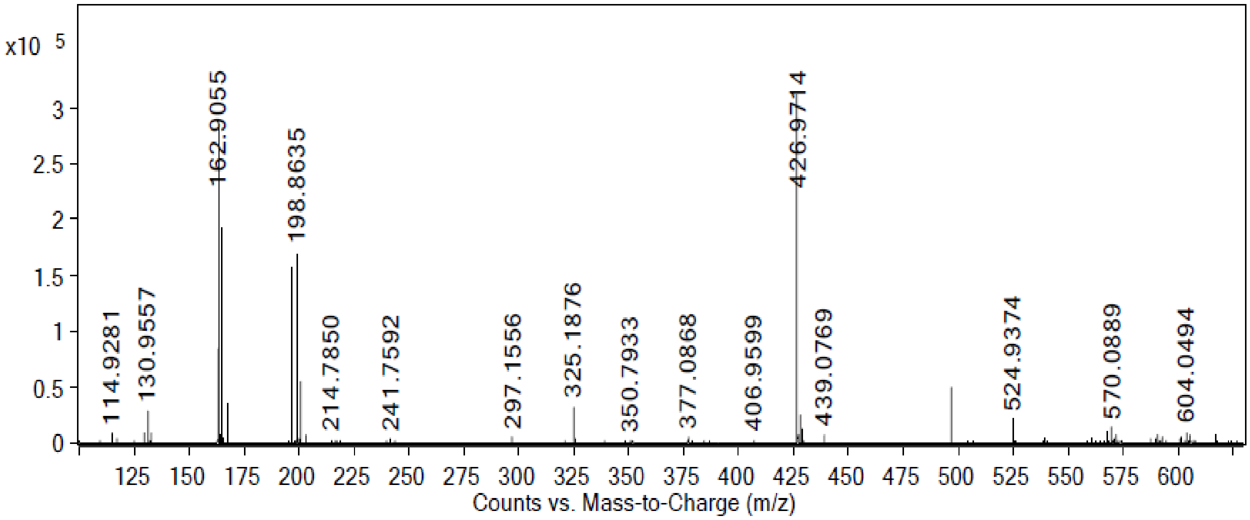

Figure S6. HRMS (ESI^+) of compound **2**

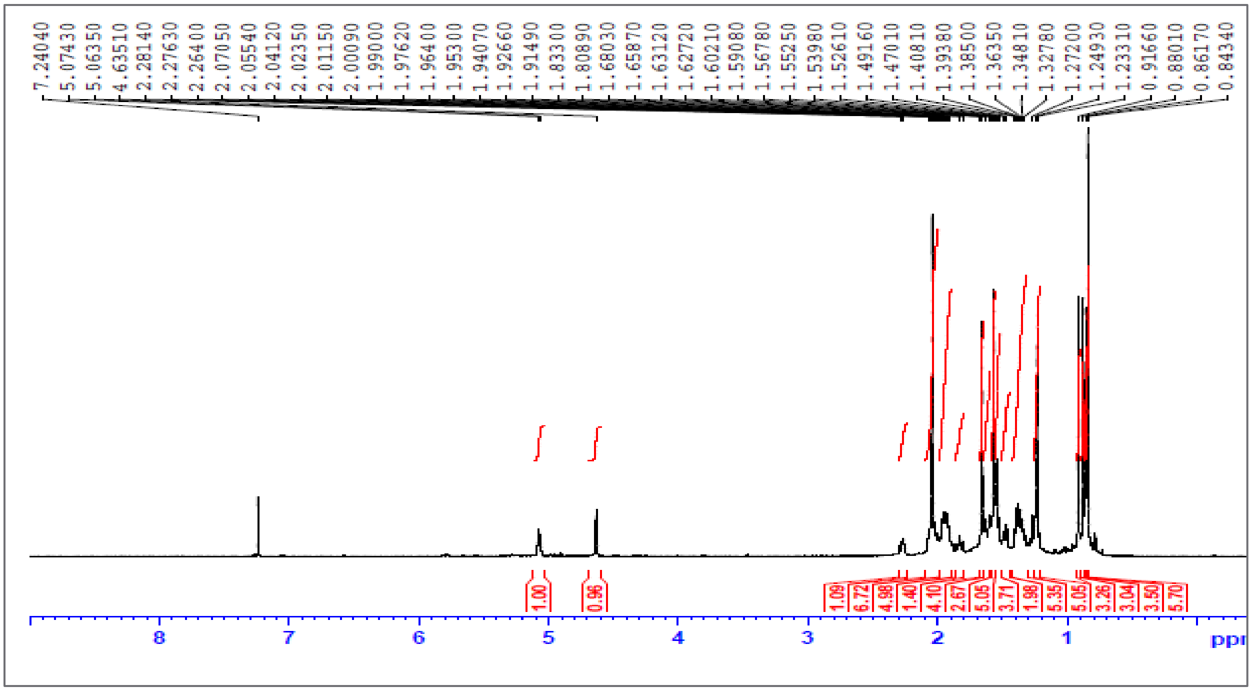

Figure S7. ^{1}H-NMR ((600 MHz, $CDCl_3$) of compound **3**

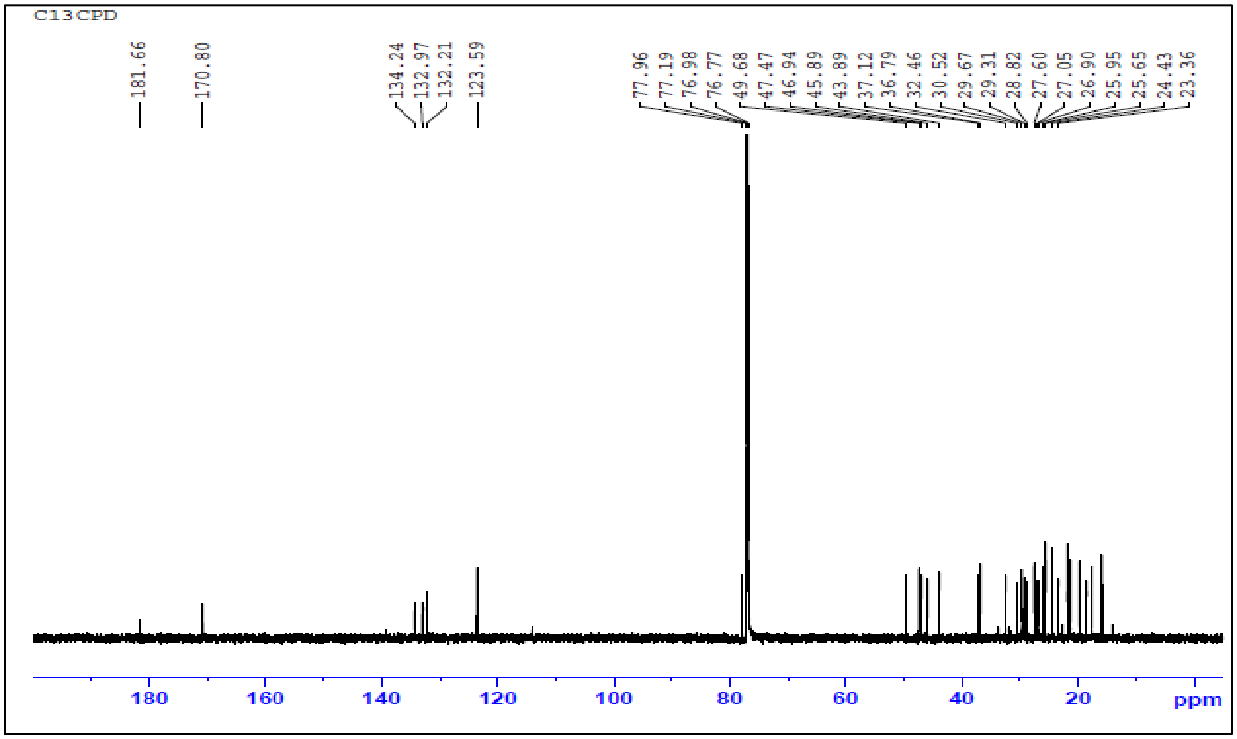

Figure S8. ^{13}C-NMR (150 MHz, $CDCl_3$) of compound **3**

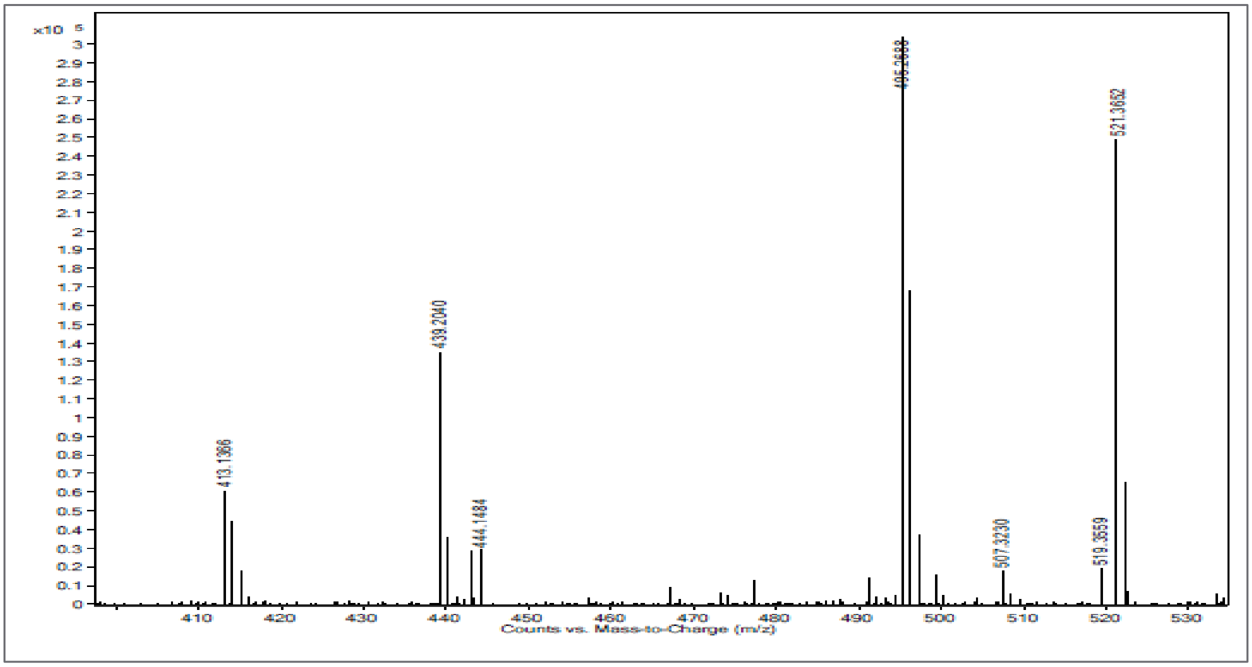

Figure S9. HRMS (ESI^+) of compound **3**

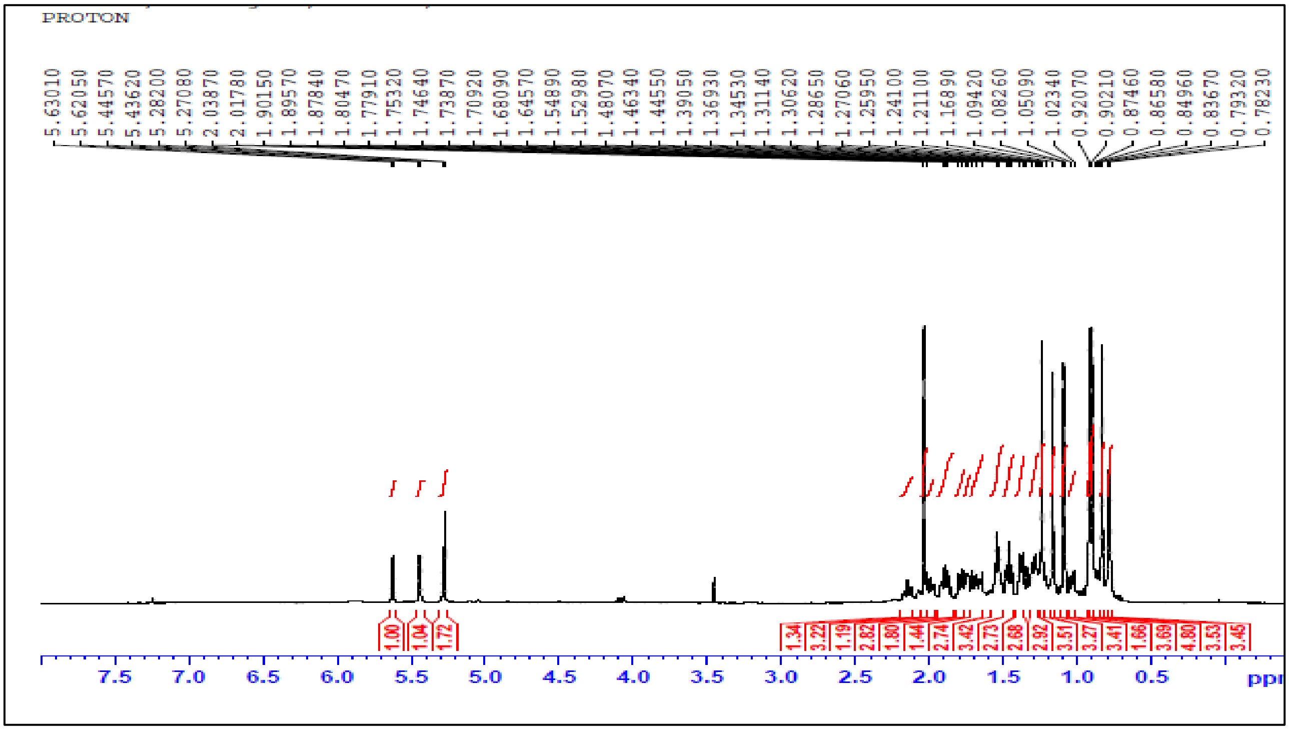

Figure S10. ^{1}H-NMR (600 MHz, $CDCl_3$) of compound **4**

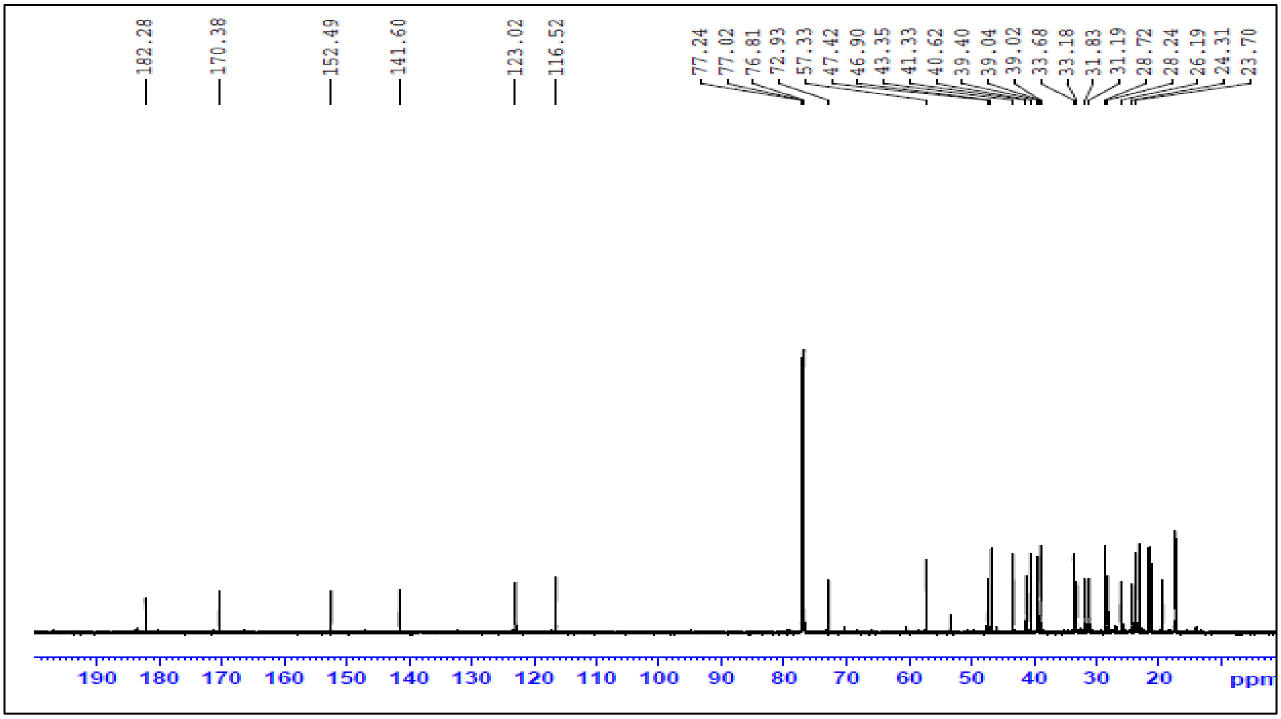

Figure S11. ^{13}C-NMR (150 MHz, $CDCl_3$) of compound **4**

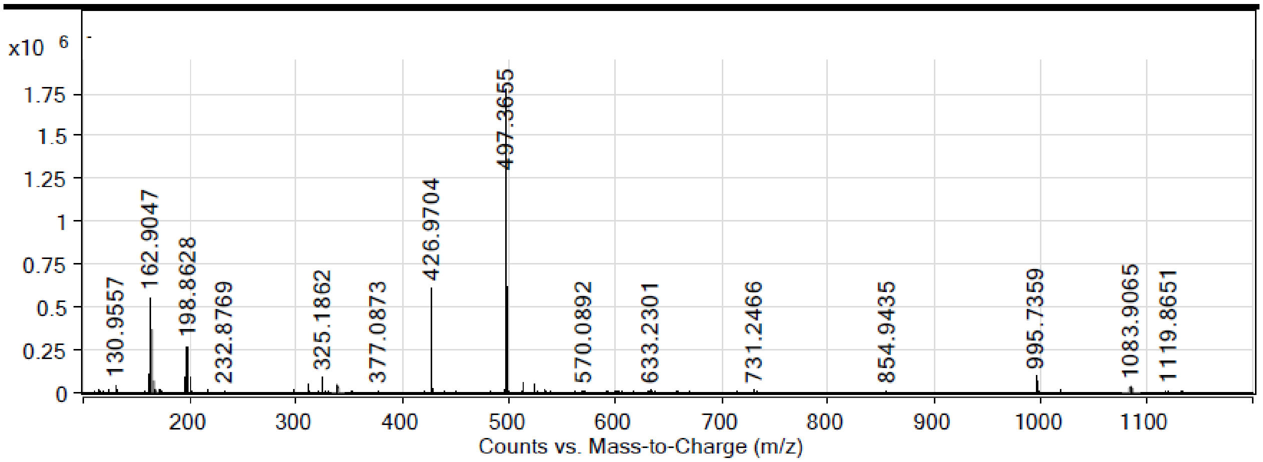

Figure S12. HRMS (ESI$^+$) of compound **4**

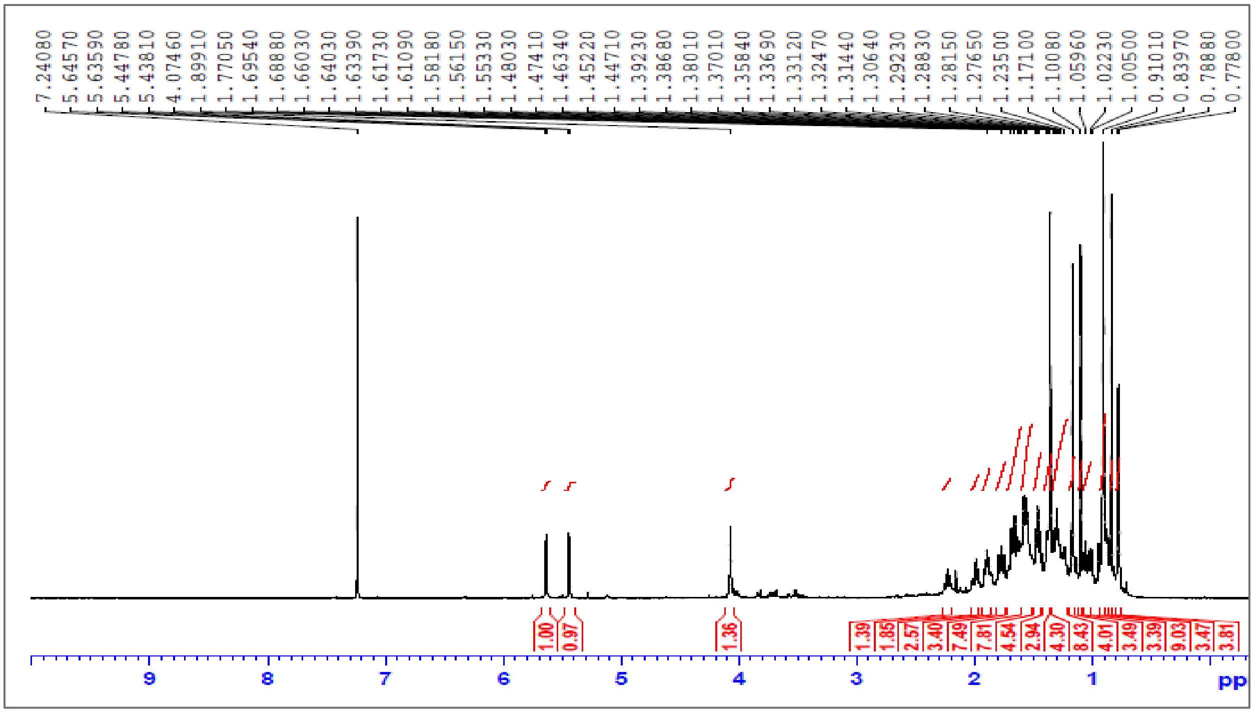

Figure S13. ^{1}H-NMR (600 MHz, $CDCl_3$) of compound **5**

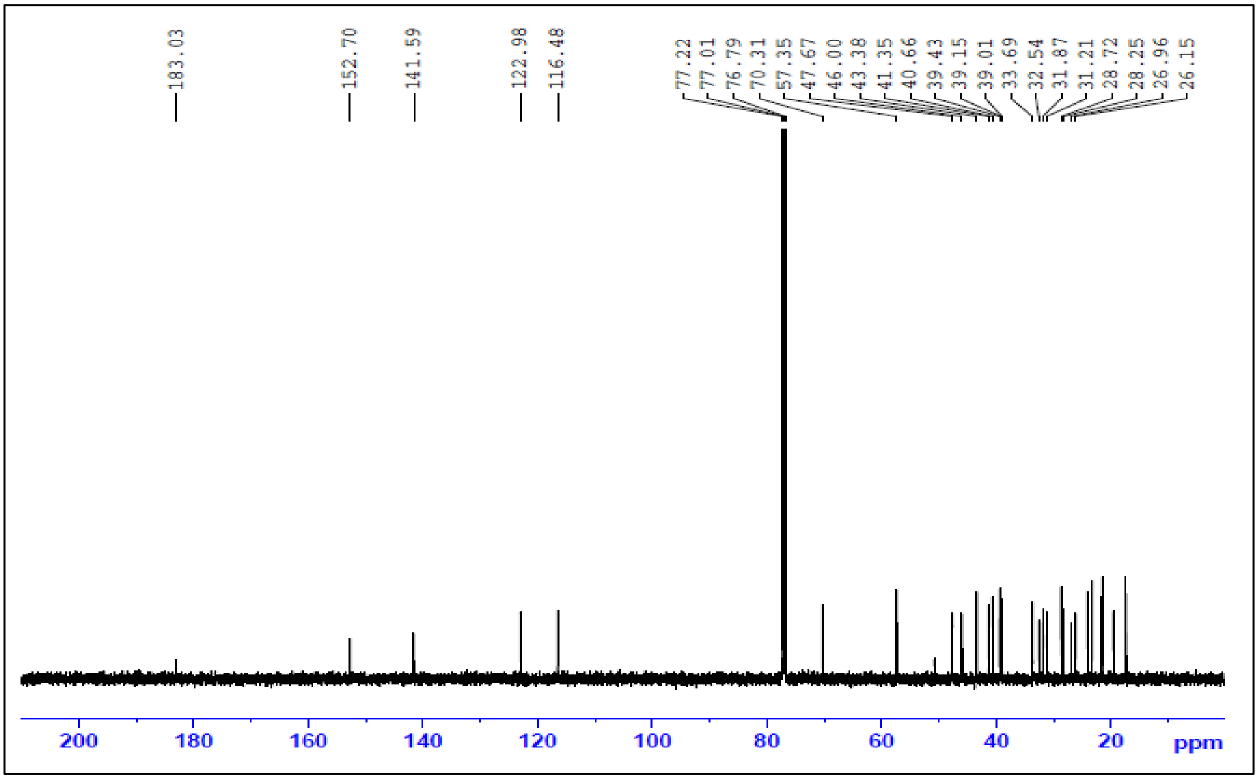

Figure S14. ^{13}C-NMR (150 MHz, $CDCl_3$) of compound **5**

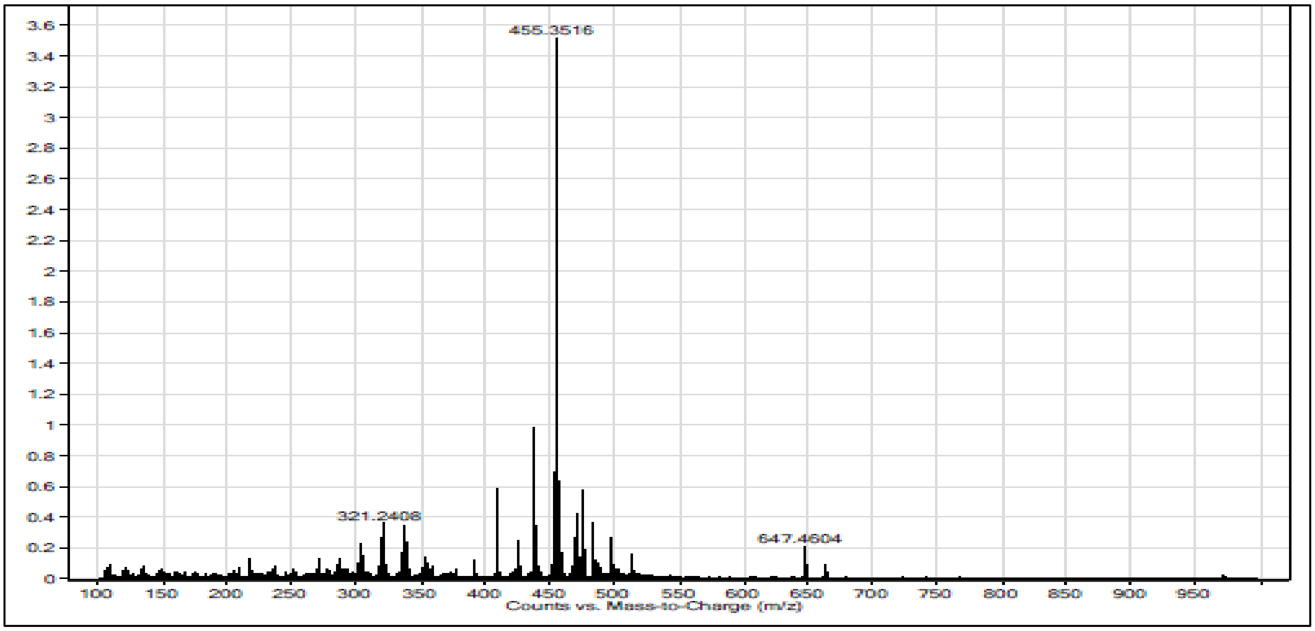

Figure S15. HRMS (ESI^+) of compound **5**

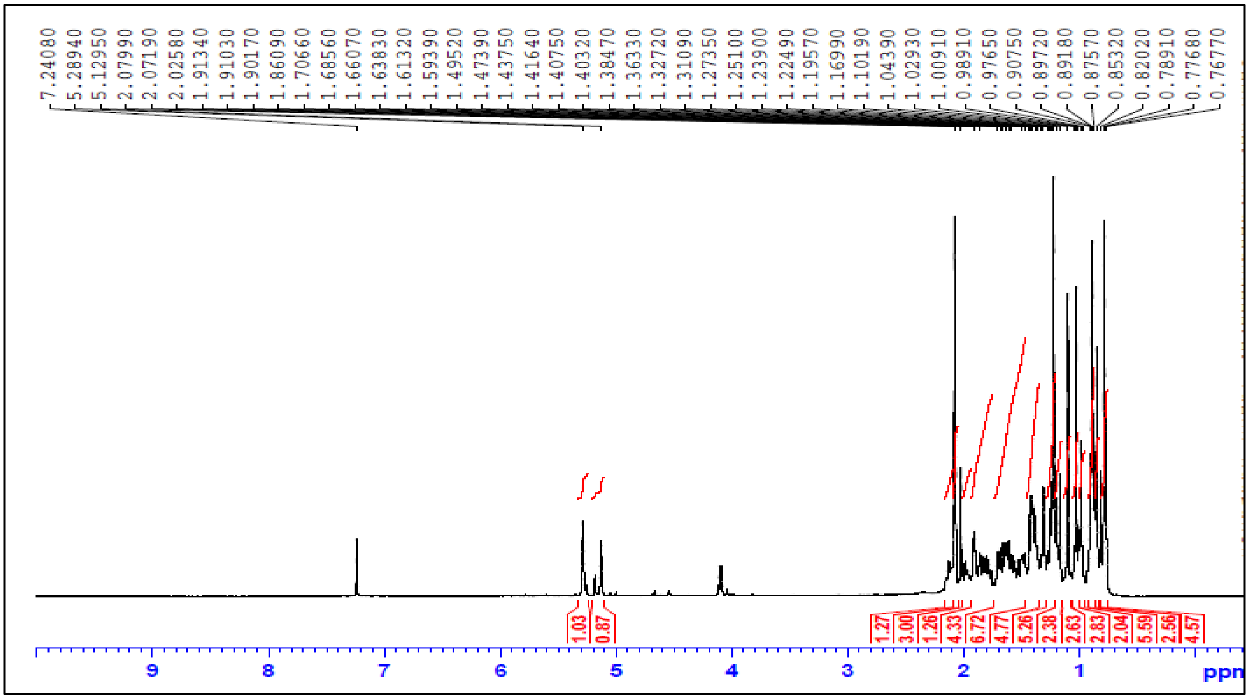

Figure S16. ^{1}H-NMR (600 MHz, $CDCl_3$) of compound **6**

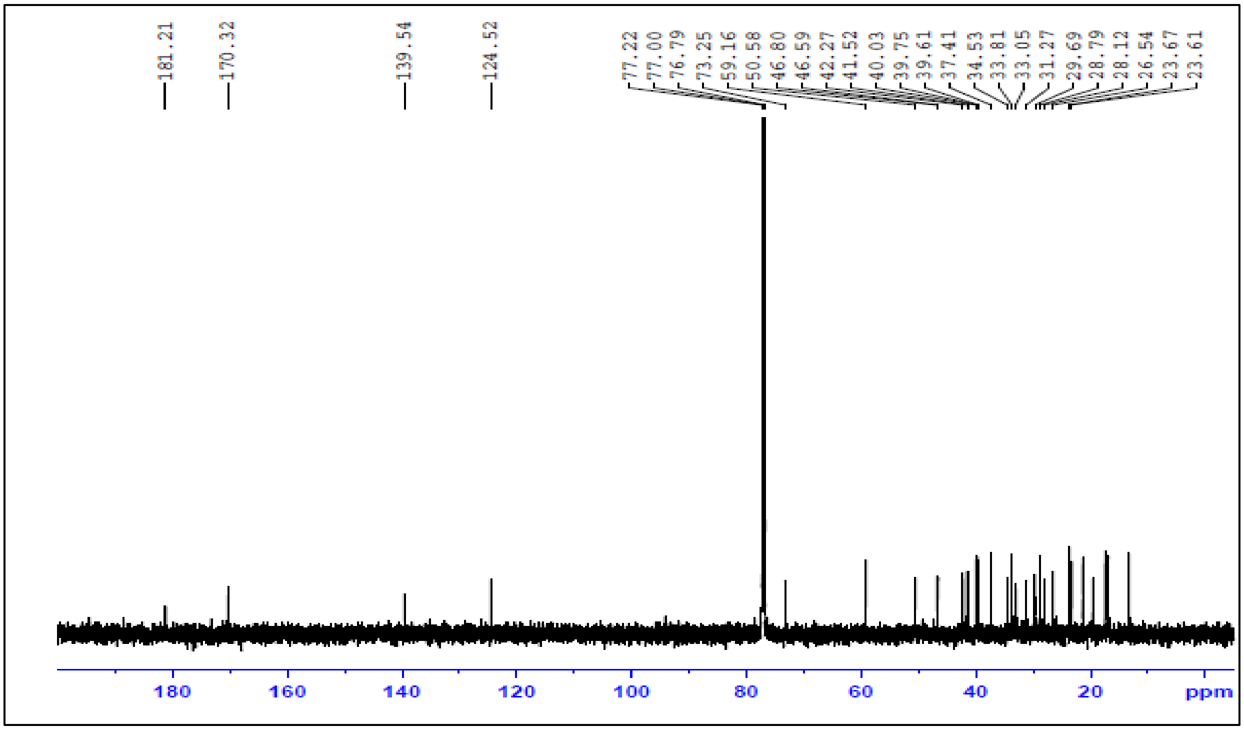

Figure S17. ^{13}C-NMR (150 MHz, $CDCl_3$) of compound **6**

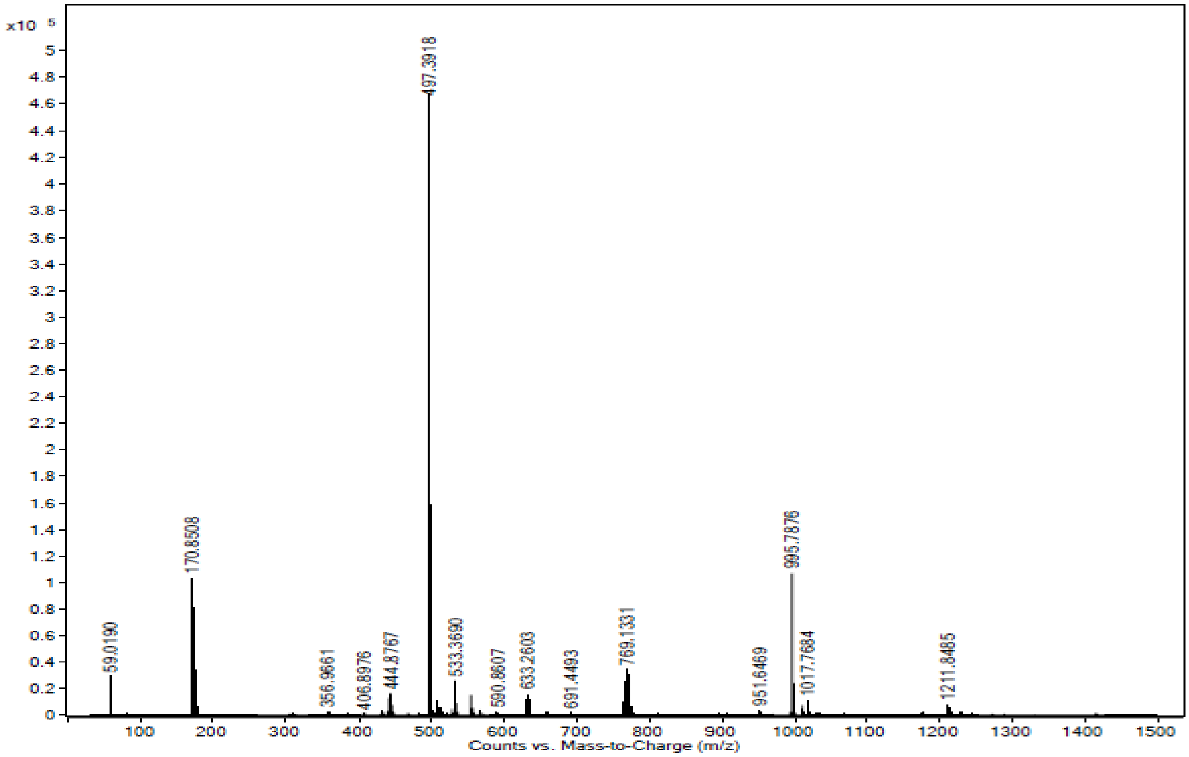

Figure S18. HRMS (ESI$^-$) of compound **6**

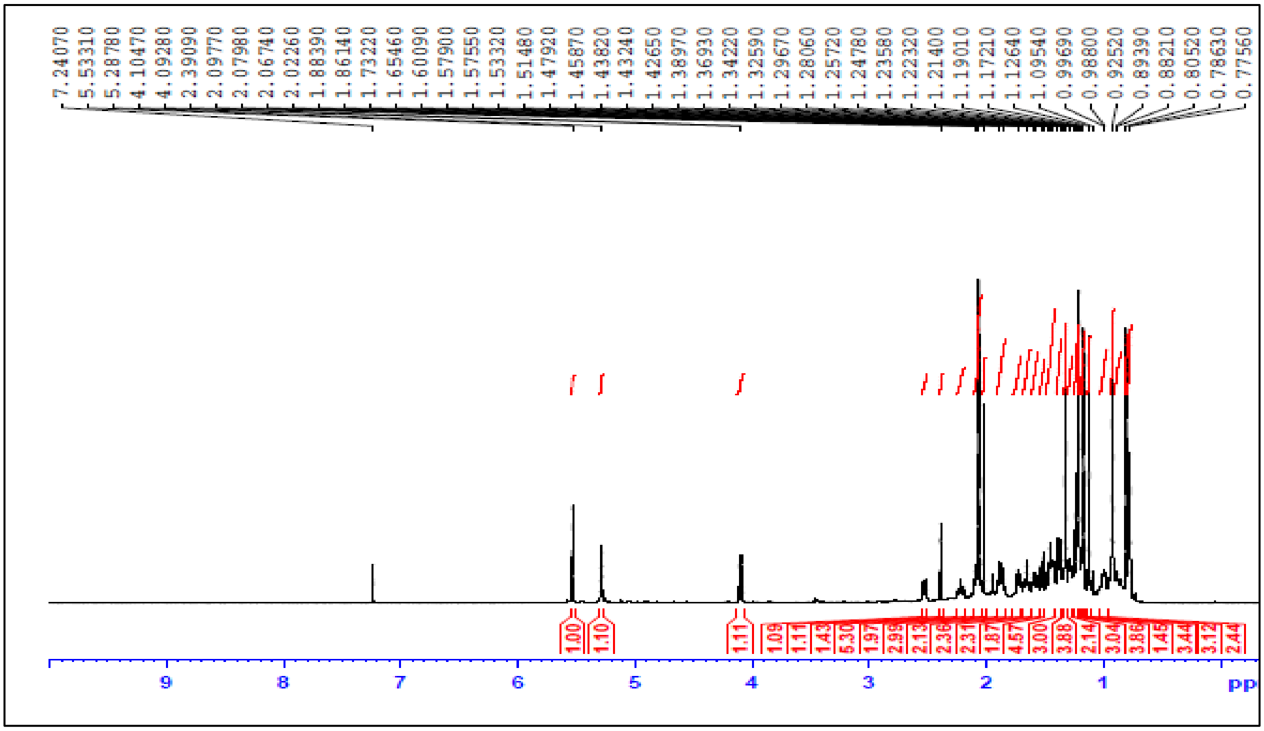

Figure S19. ^{1}H-NMR (600 MHz, $CDCl_3$) of compound **7**

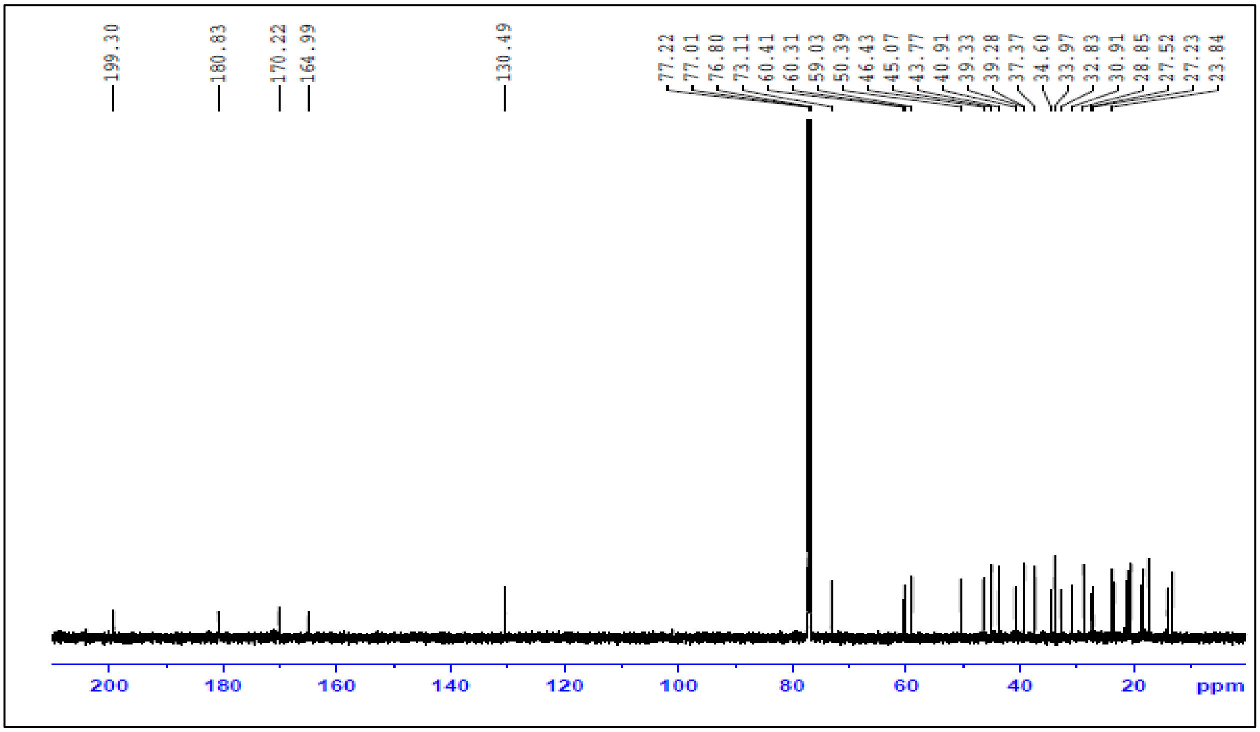

Figure S20. ^{13}C-NMR (150 MHz, $CDCl_3$) of compound **7**

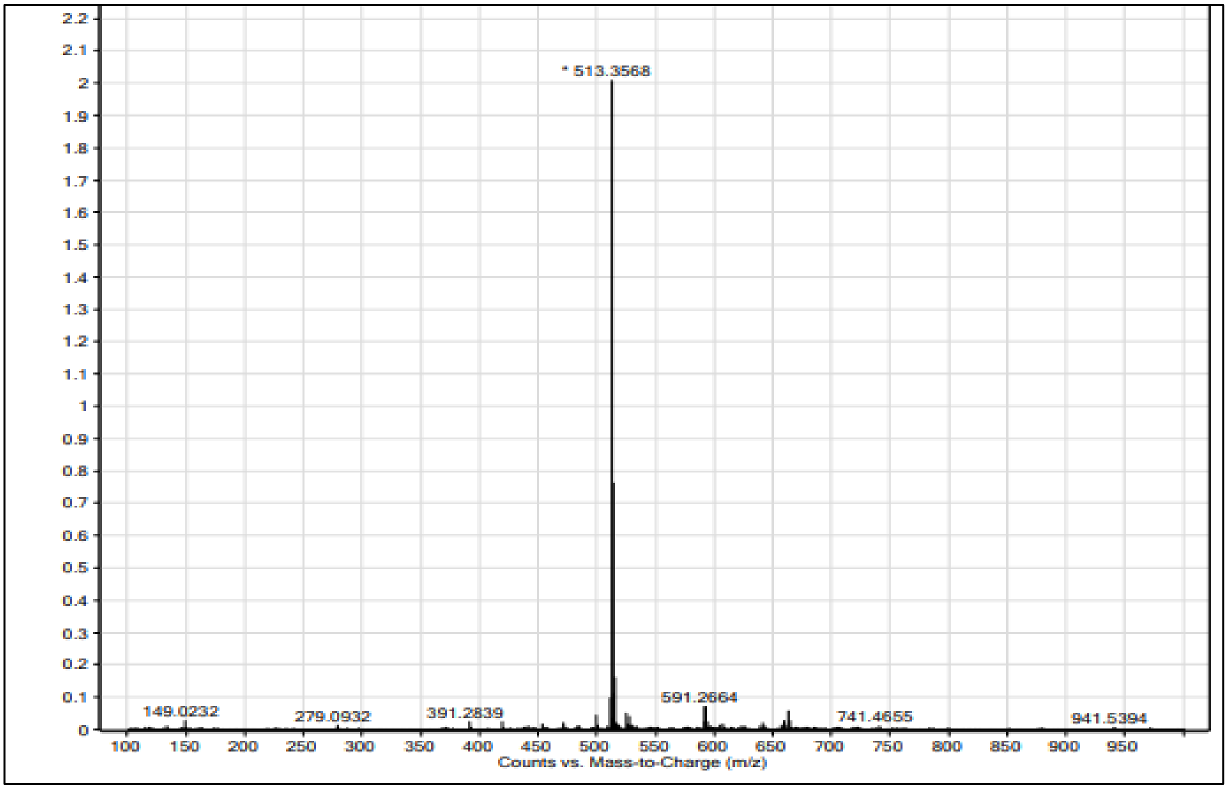

Figure S21. HRMS (ESI^+) of compound **7**

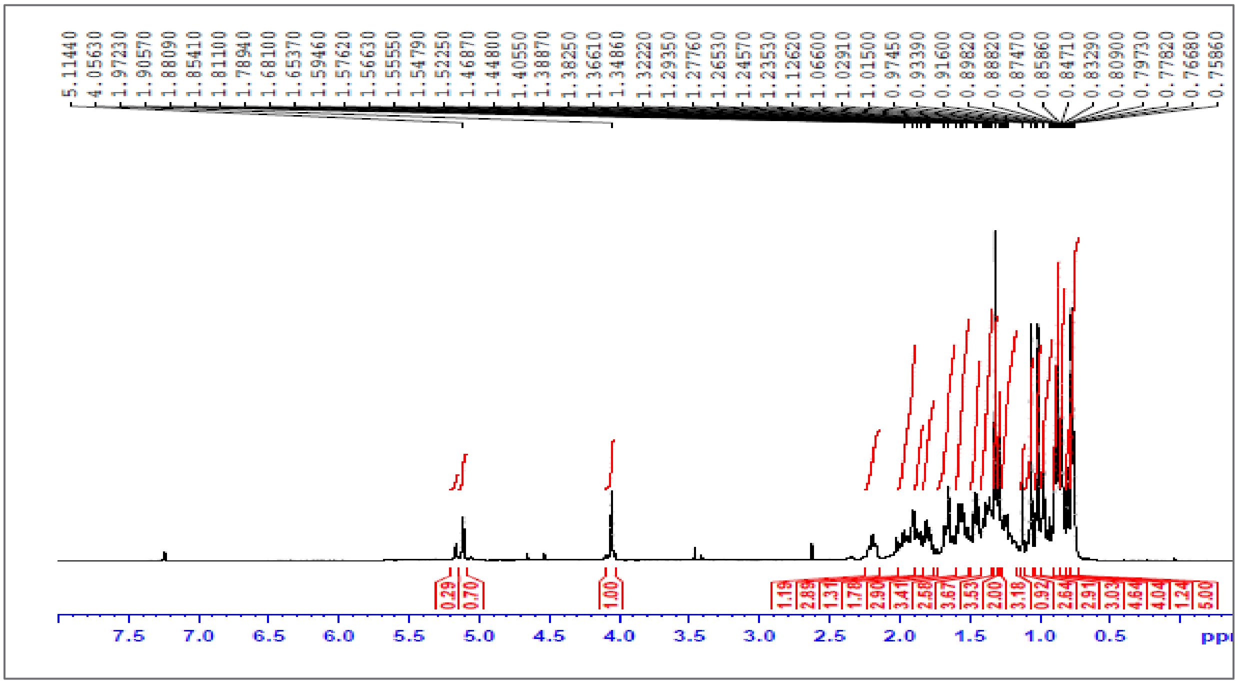

Figure S22. ^{1}H-NMR (600 MHz, $CDCl_3$) of compound **8**

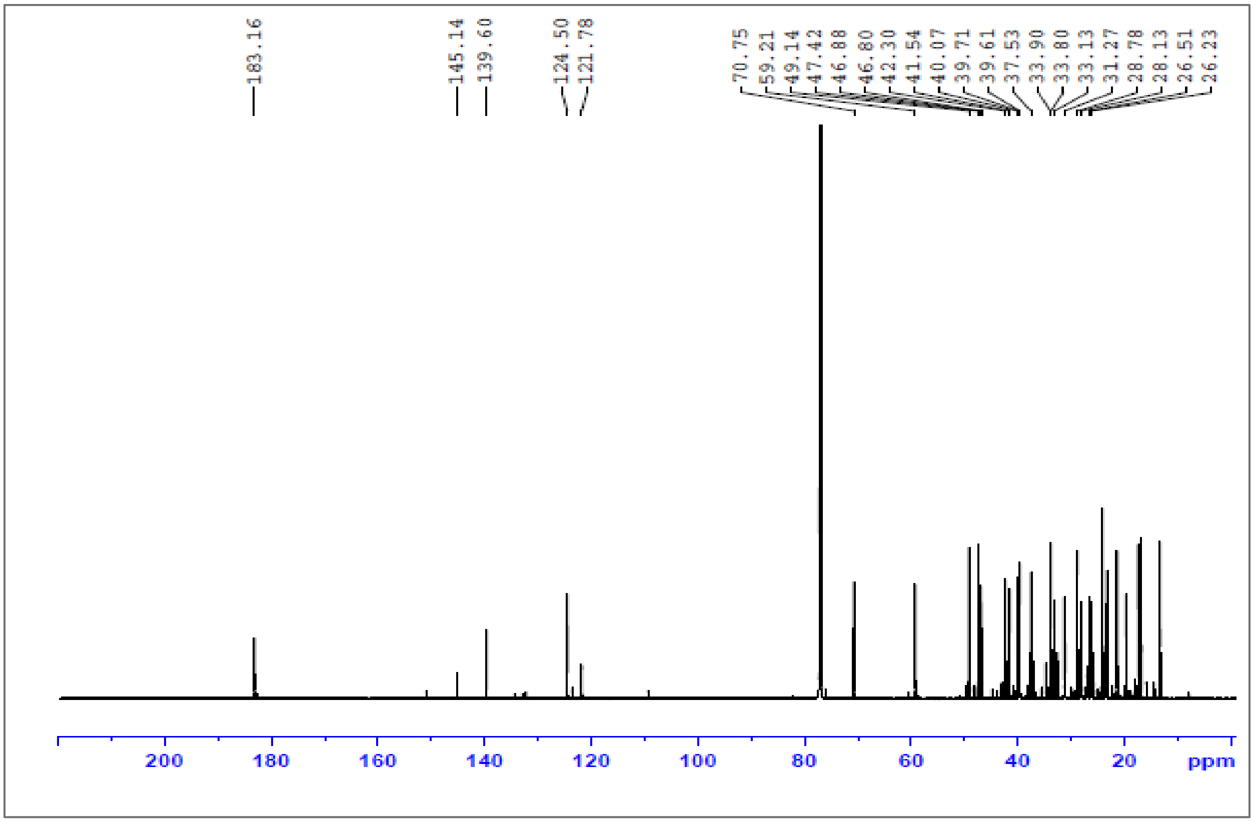

Figure S23. ^{13}C-NMR ((150 MHz, $CDCl_3$) of compound **8**

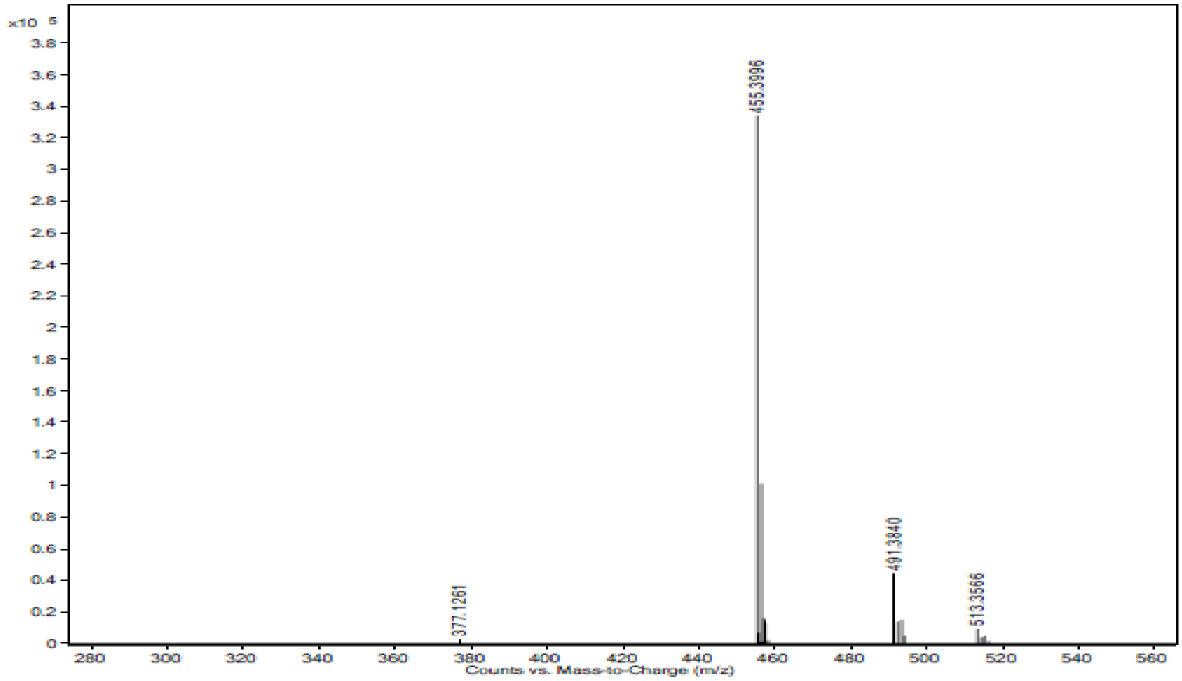

Figure S24. HRMS (ESI$^-$) of compound **8**

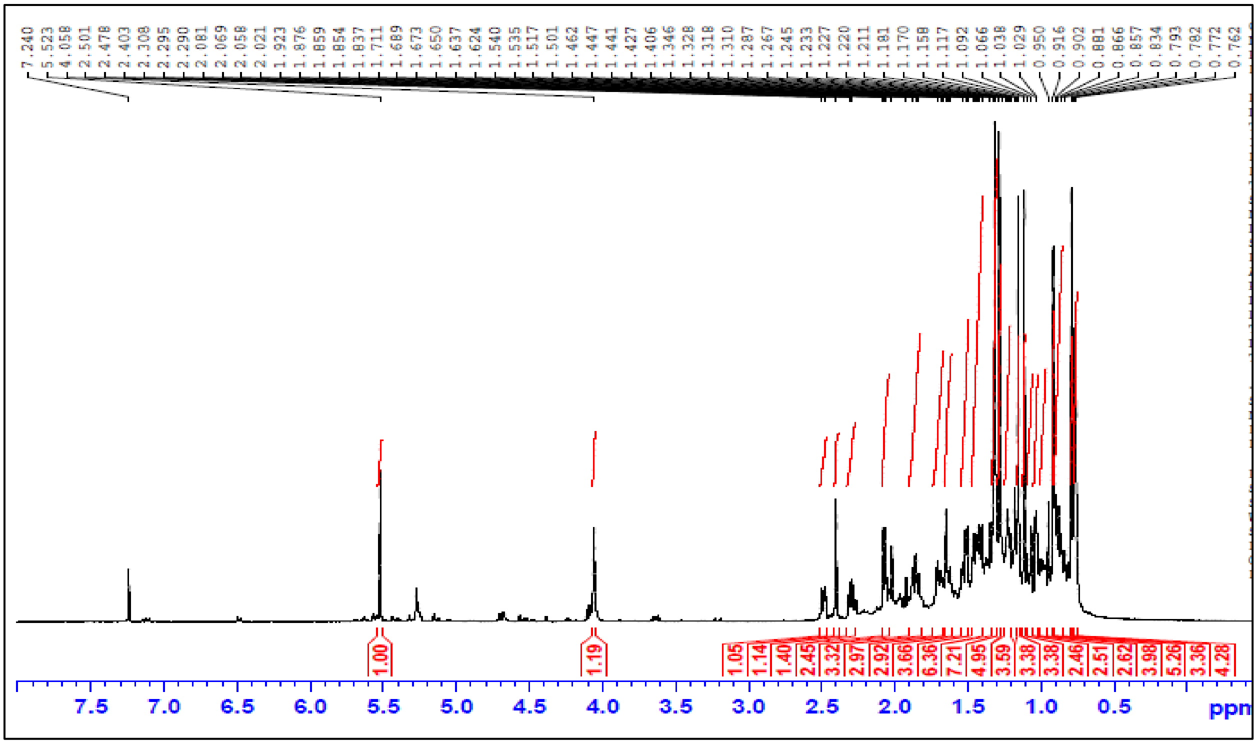

Figure S25. ^{1}H-NMR (600 MHz, $CDCl_3$) of compound **9**

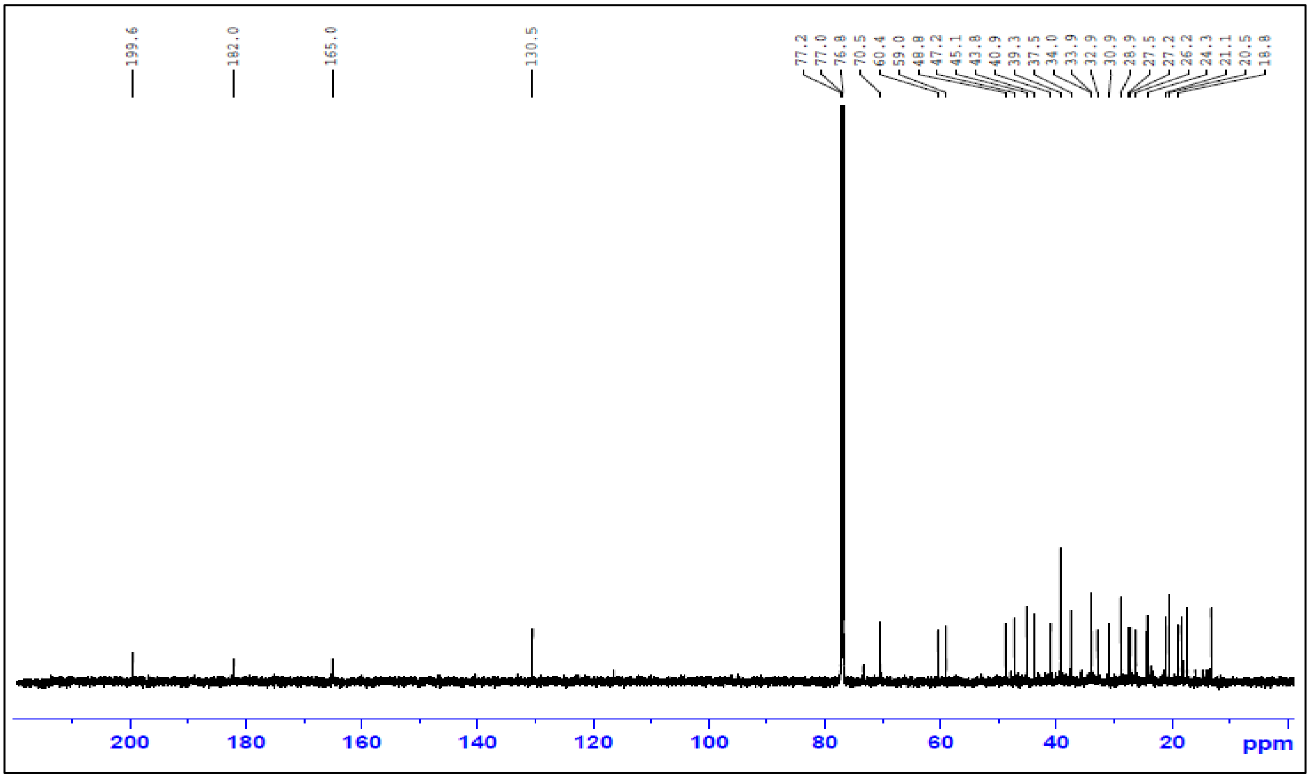

Figure S26. ^{13}C-NMR (150 MHz, $CDCl_3$) of compound **9**

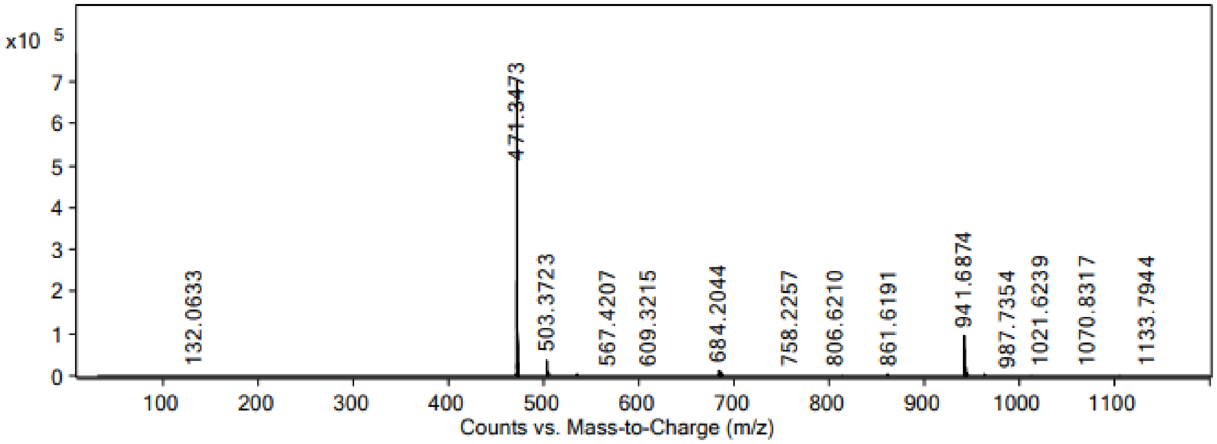

Figure S27. ESI-MS (positive) of compound **9**

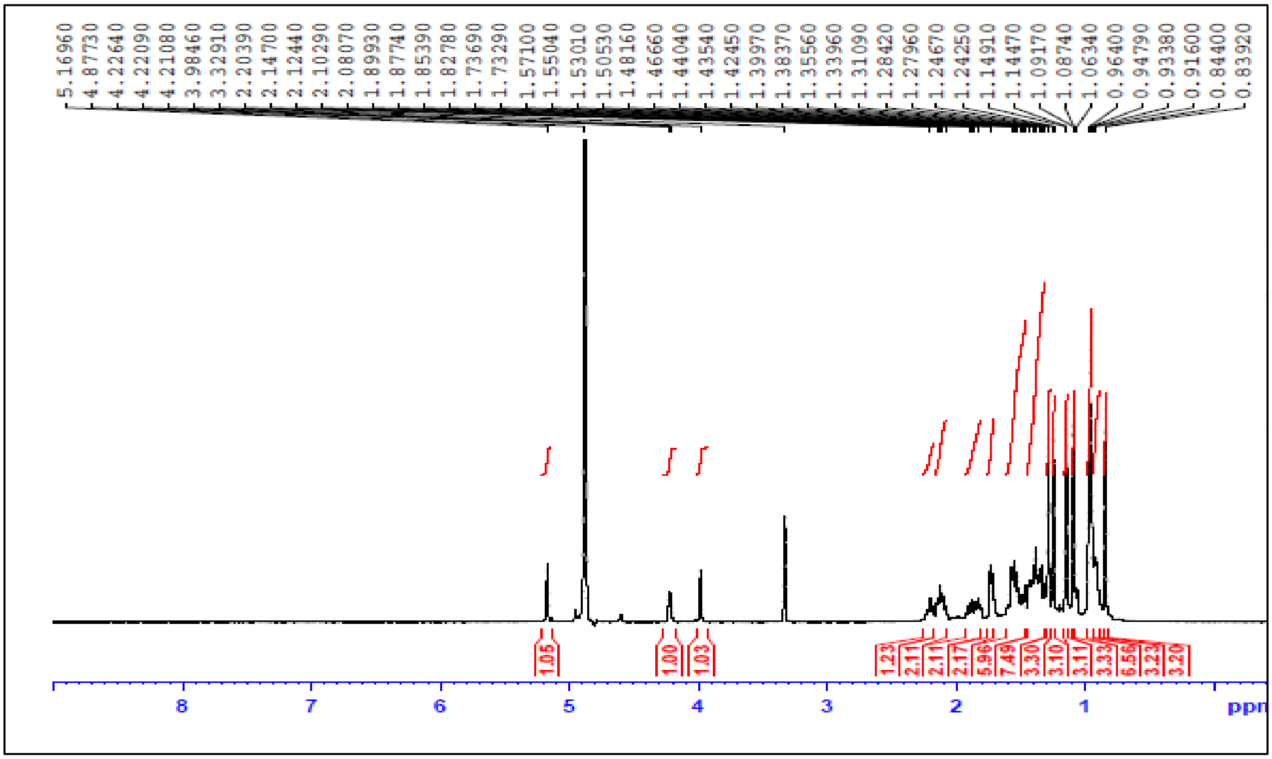

Figure S28. ^{1}H-NMR (600 MHz, $CDCl_3$) of compound **10**

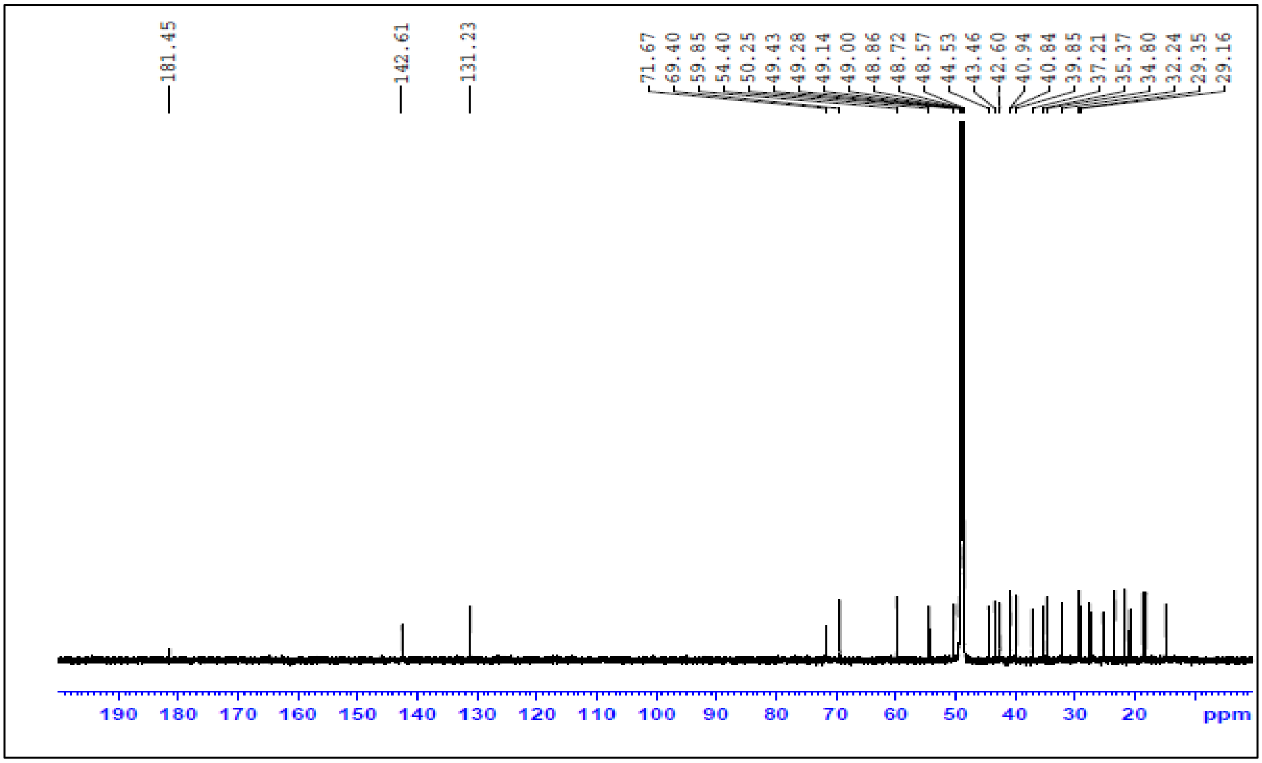

Figure S29. ^{13}C-NMR (150 MHz, $CDCl_3$) of compound **10**

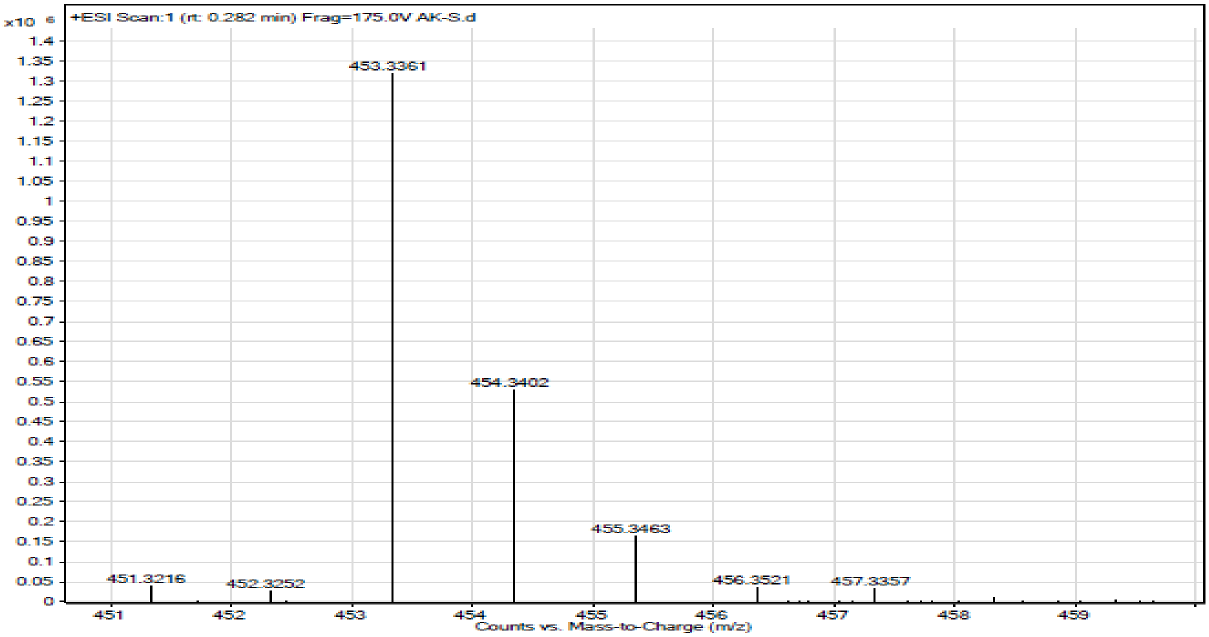

Figure S30. HRMS (ESI^+) of compound **10**

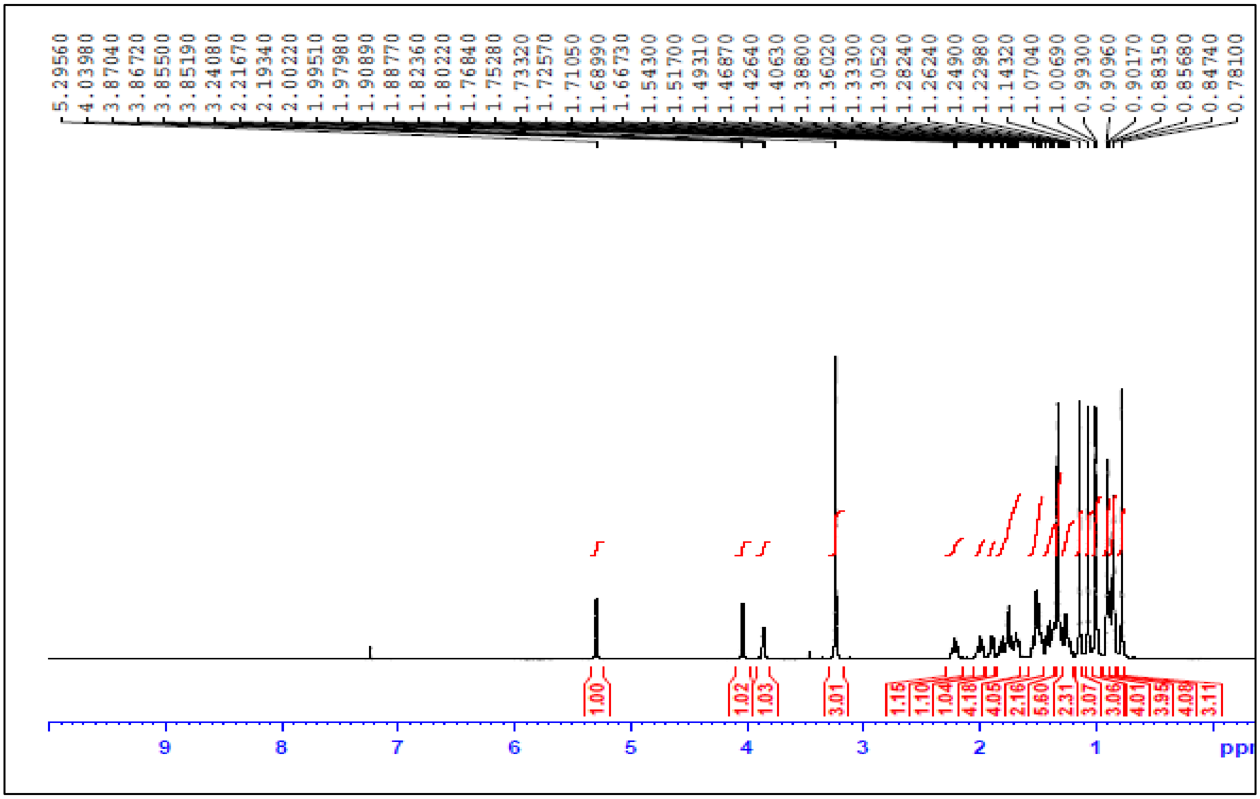

Figure S31. ^{1}H-NMR (600 MHz, $CDCl_3$) of compound **11**

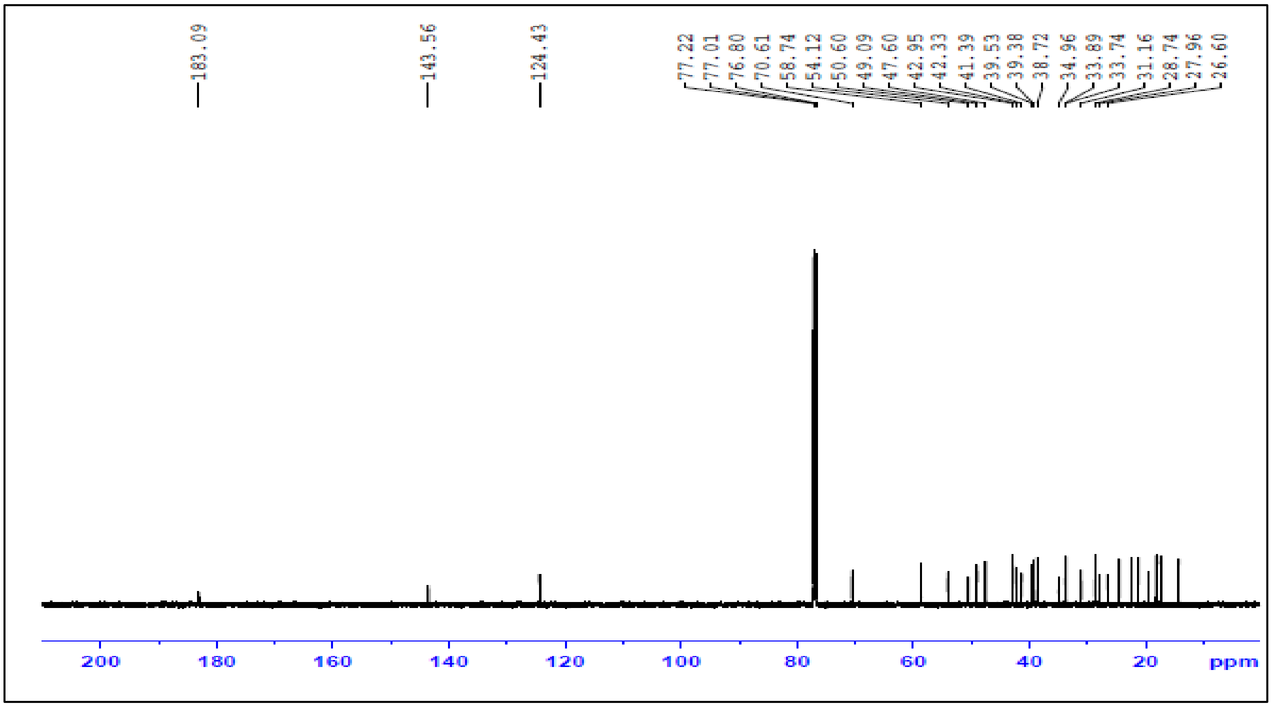

Figure S32. ^{13}C-NMR (150 MHz, $CDCl_3$) of compound **11**

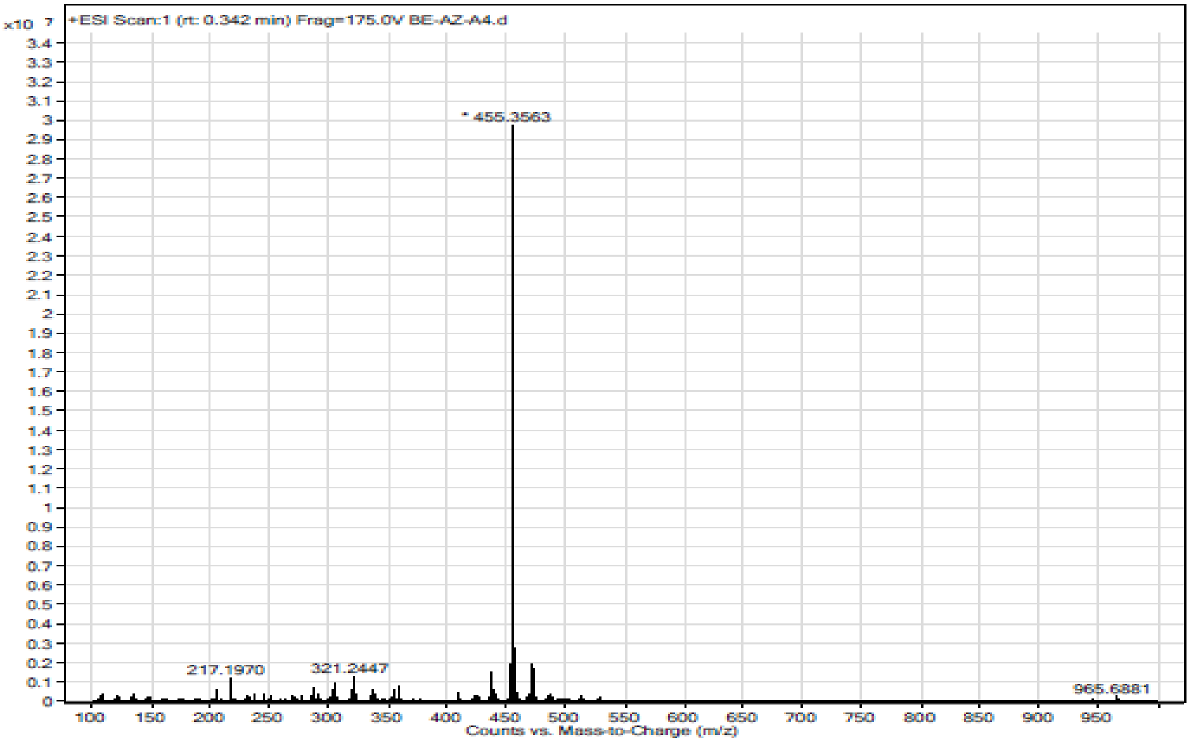

Figure S33. HRMS (ESI^+) of compound **11**

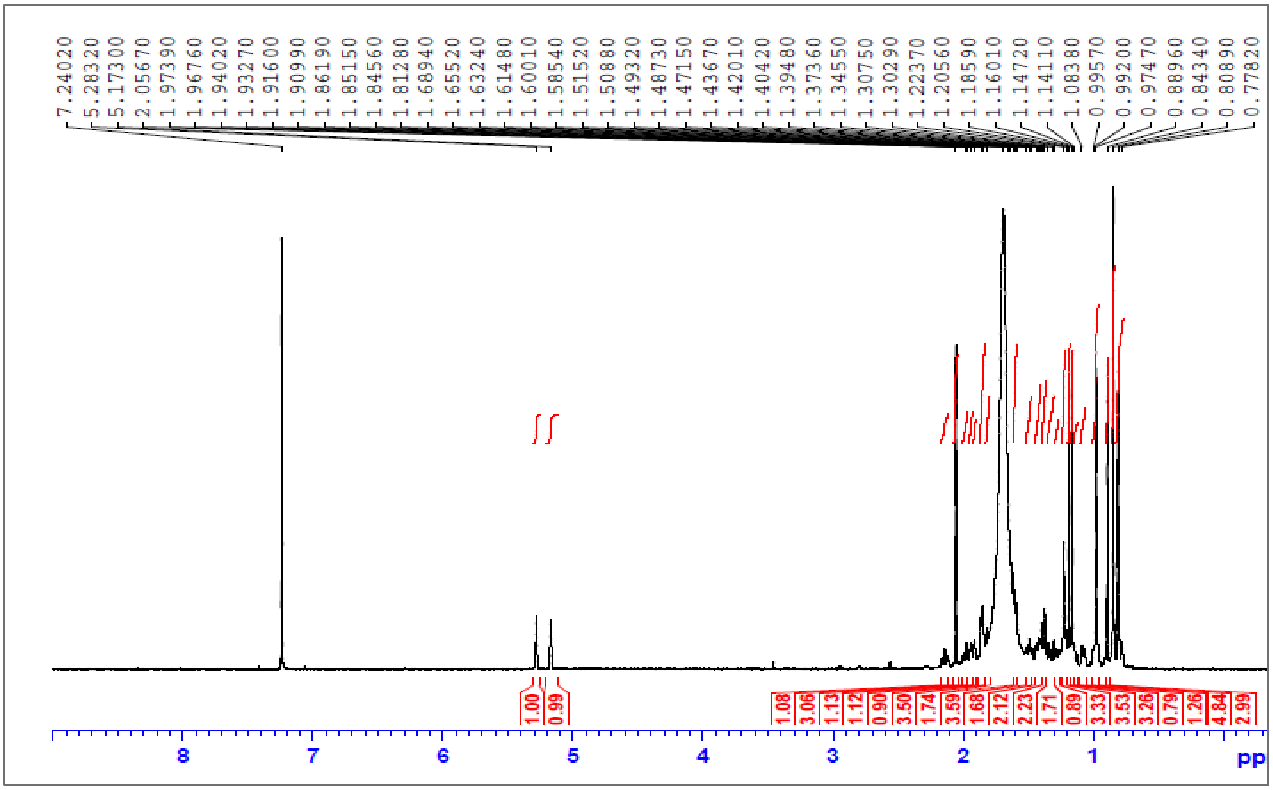

Figure S34. ^{1}H-NMR (600 MHz, $CDCl_3$) of compound **12**

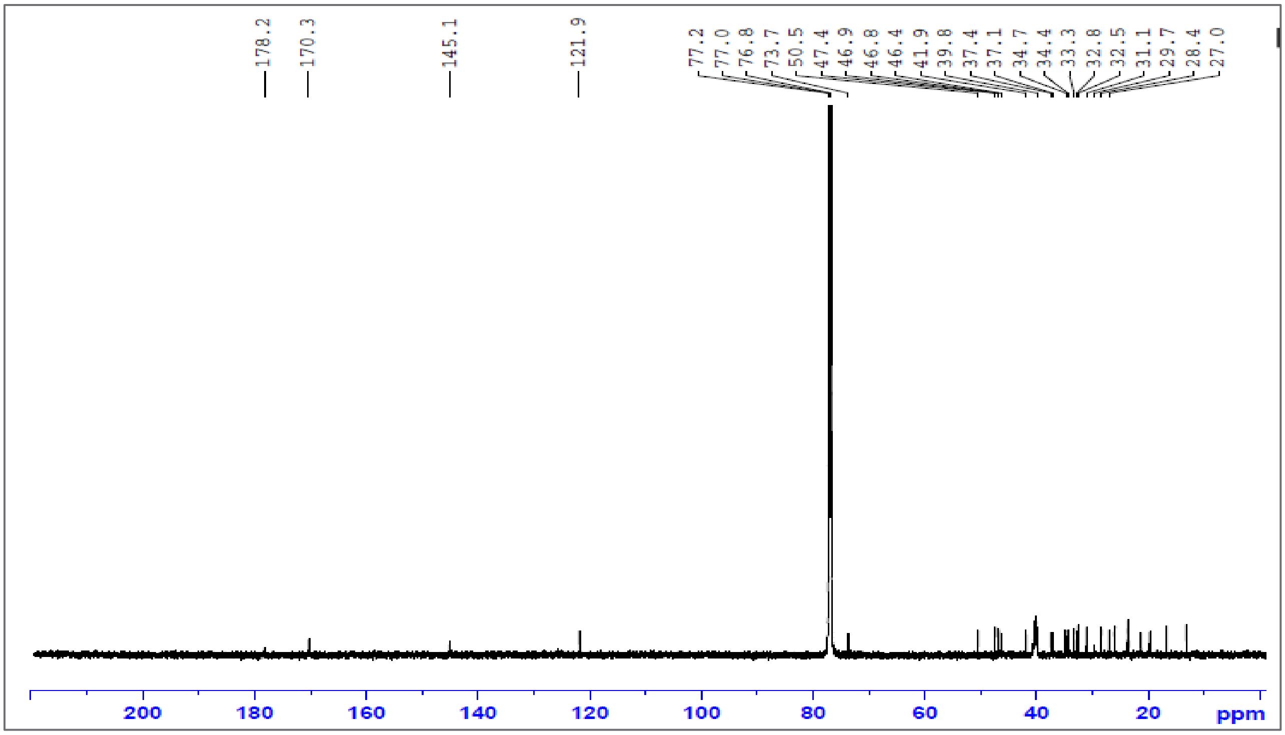

Figure S35. ^{13}C-NMR (150 MHz, $CDCl_3$) of compound **12**

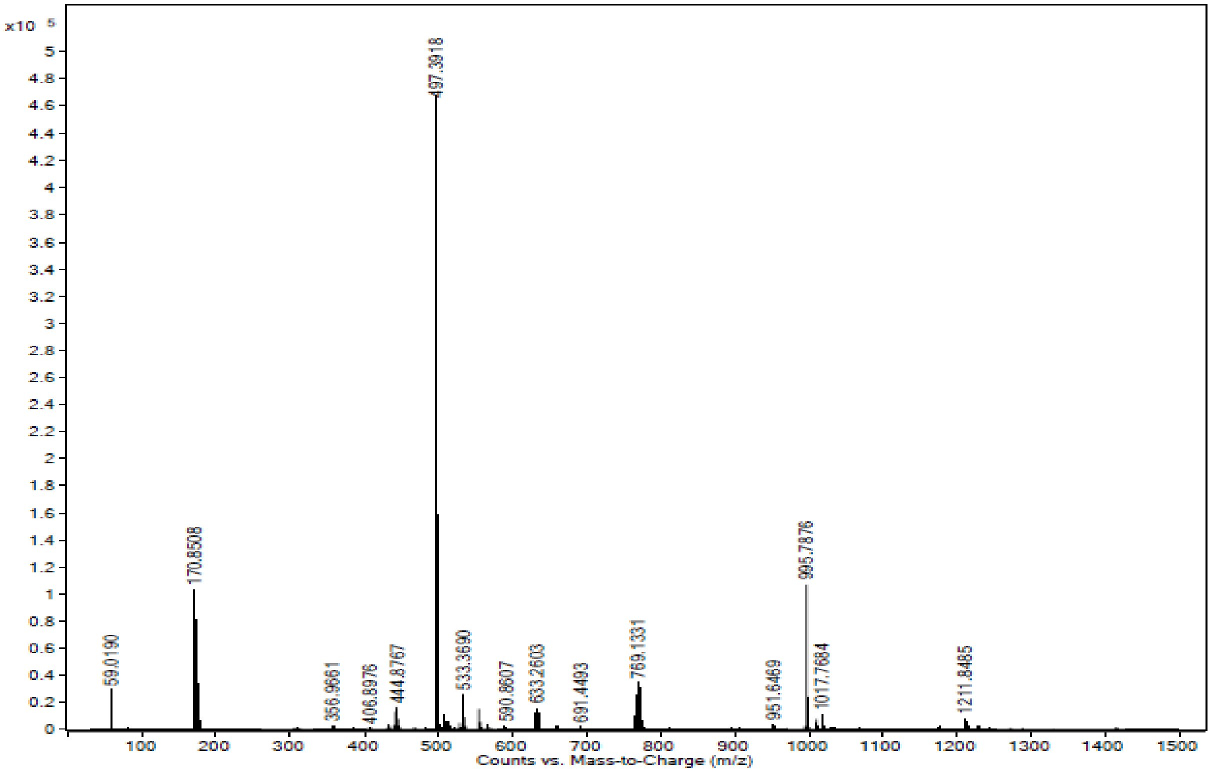

Figure S36. HRMS (ESI$^-$) of compound **12**

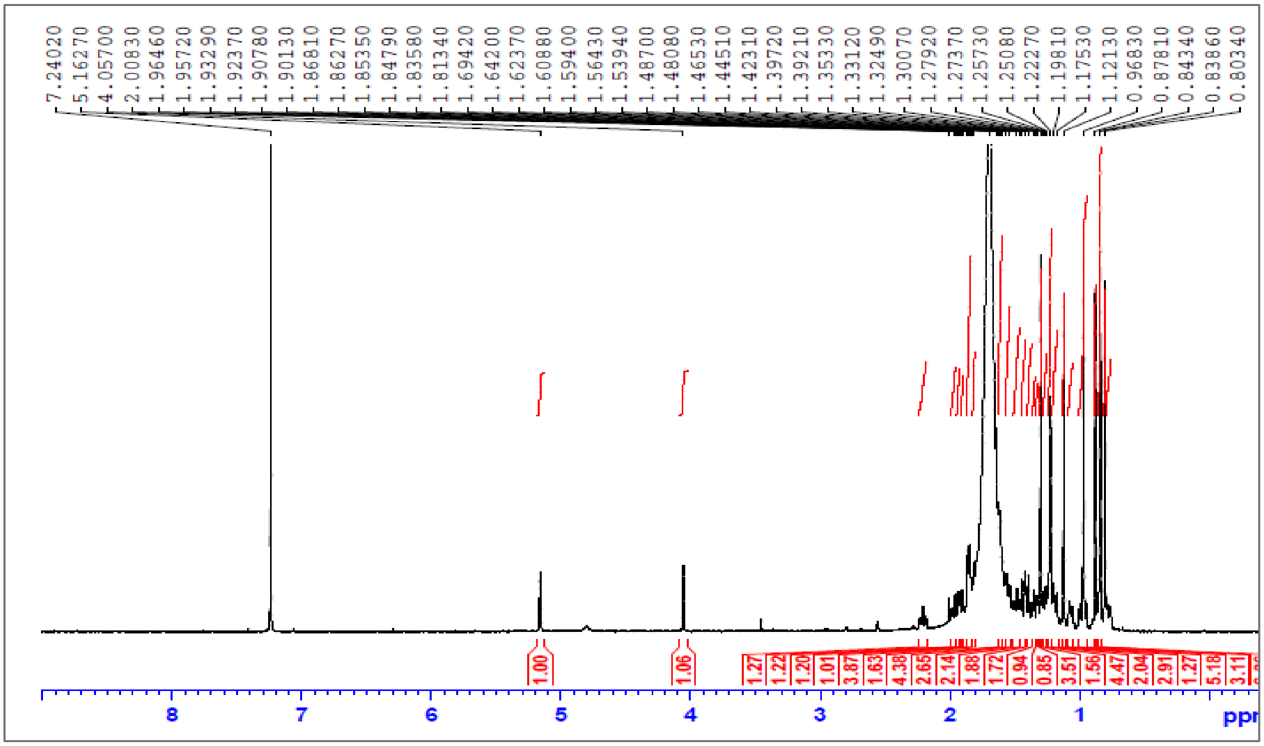

Figure S37. ^{1}H-NMR (600 MHz, $CDCl_3$) of compound **13**

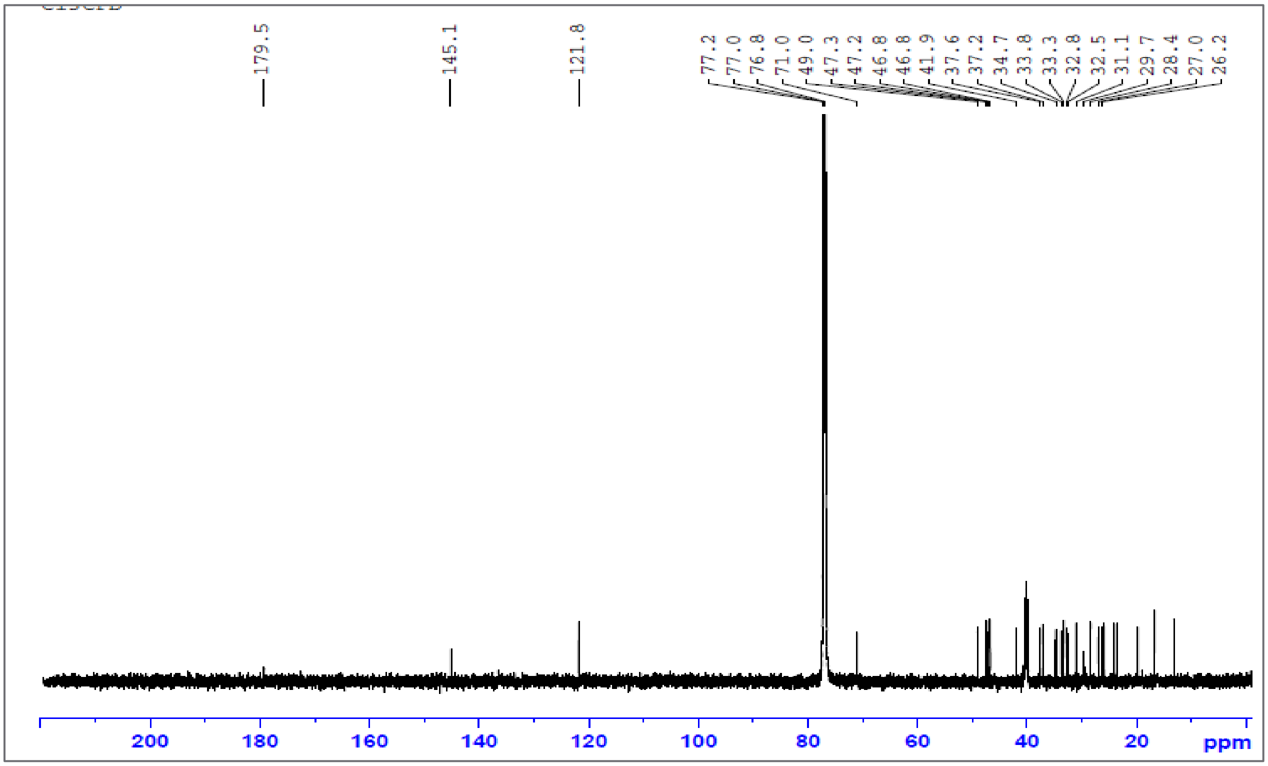

Figure S38. ^{13}C-NMR (150 MHz, $CDCl_3$) of compound **13**

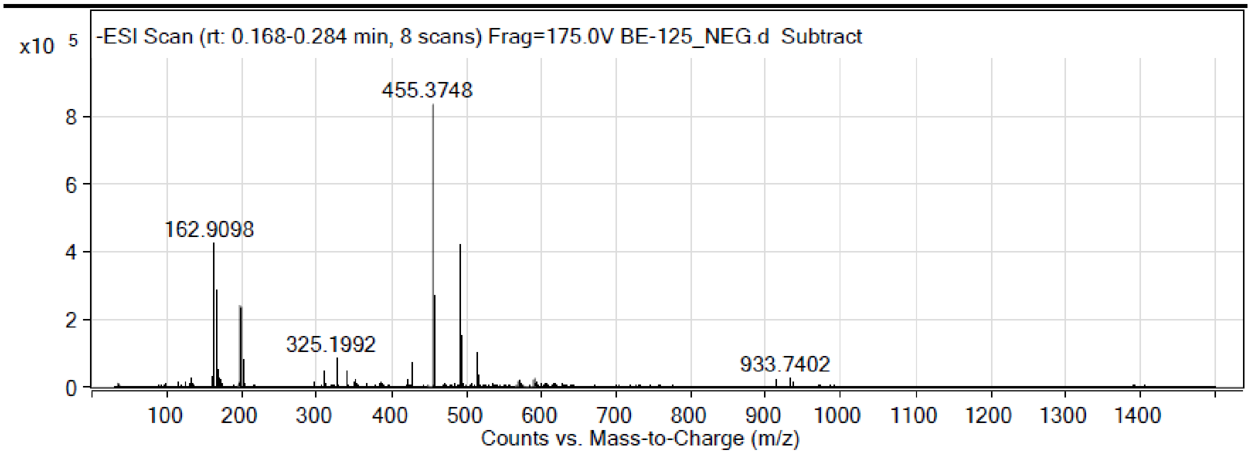

Figure S39. HRMS (ESI⁻) of compound **13**

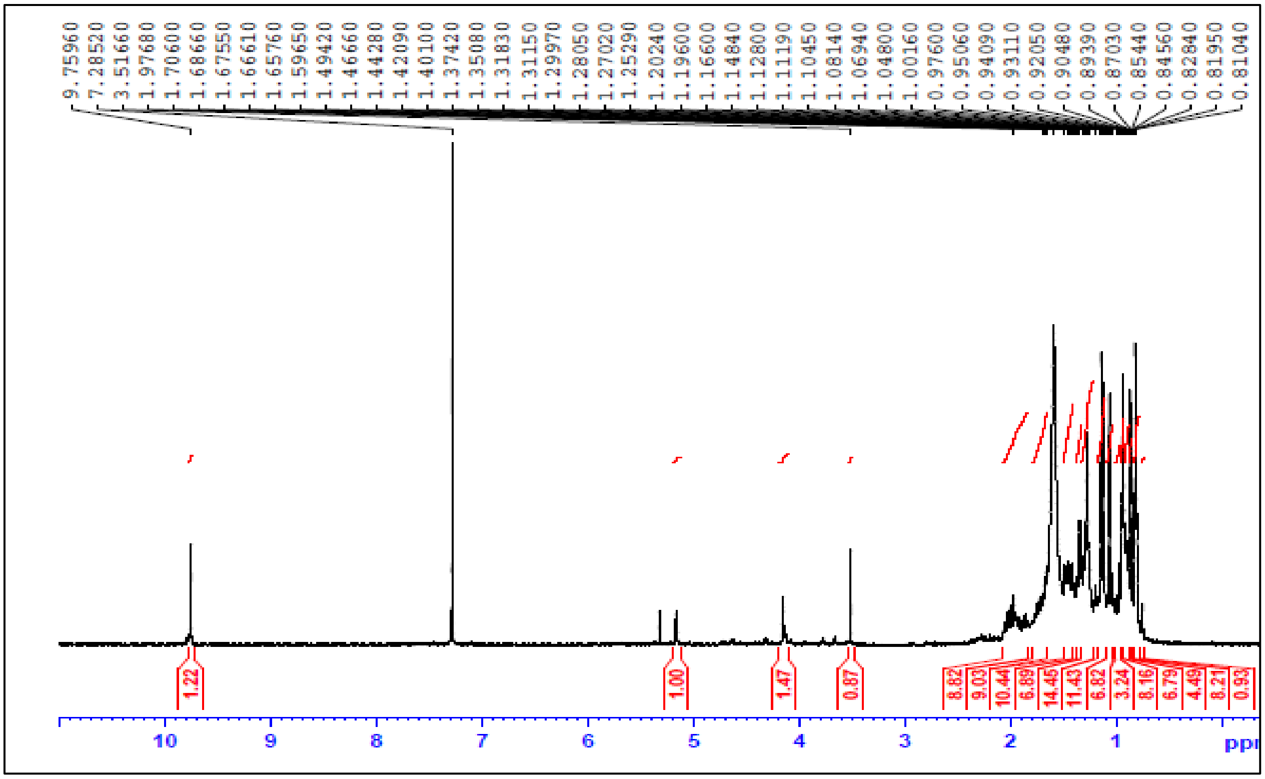

Figure S40. ^{1}H-NMR (600 MHz, $CDCl_3$) of compound **14**

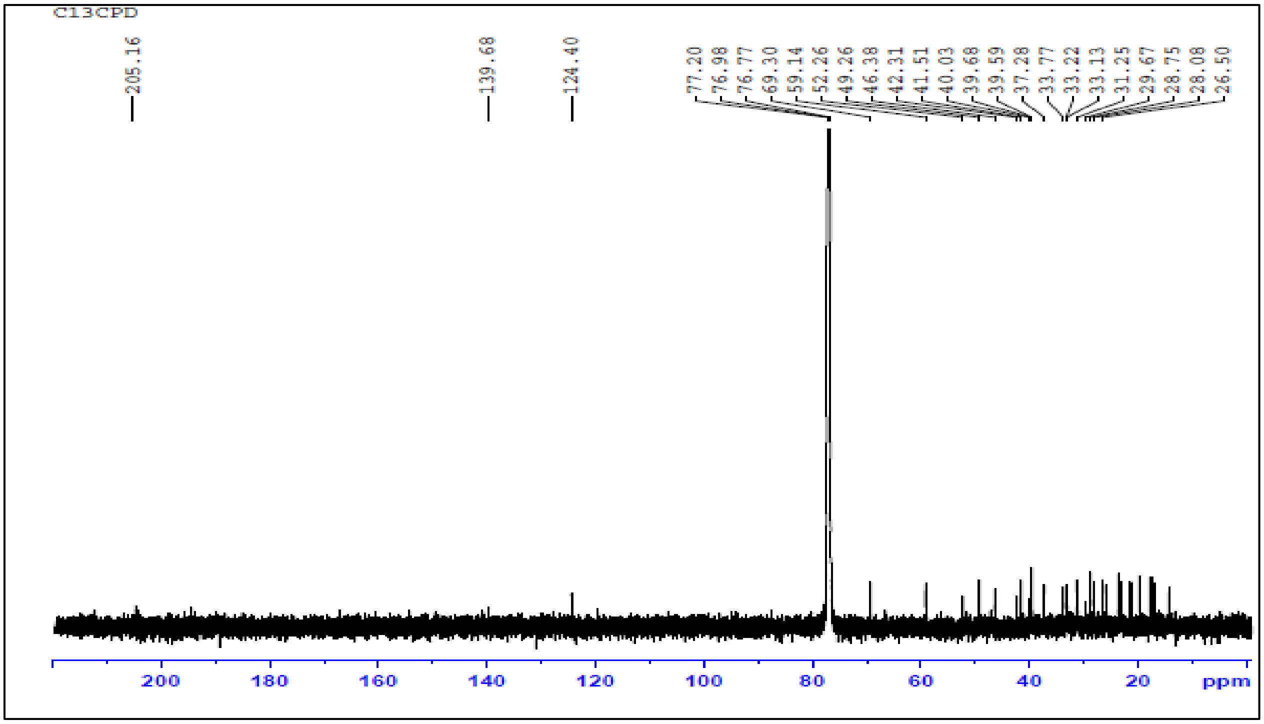

Figure S41. ^{13}C-NMR (150 MHz, $CDCl_3$) of compound **14**

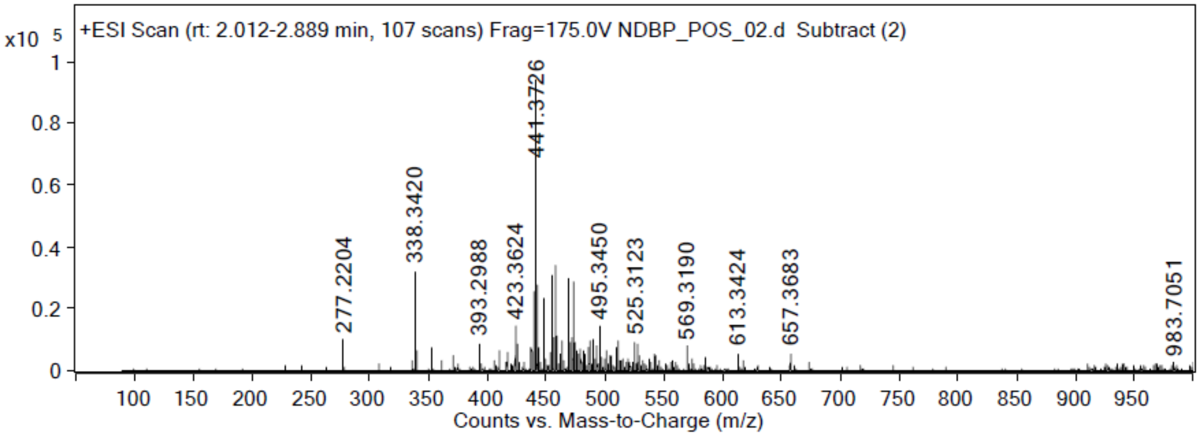

Figure S42. HRMS (ESI$^+$) of compound **14**

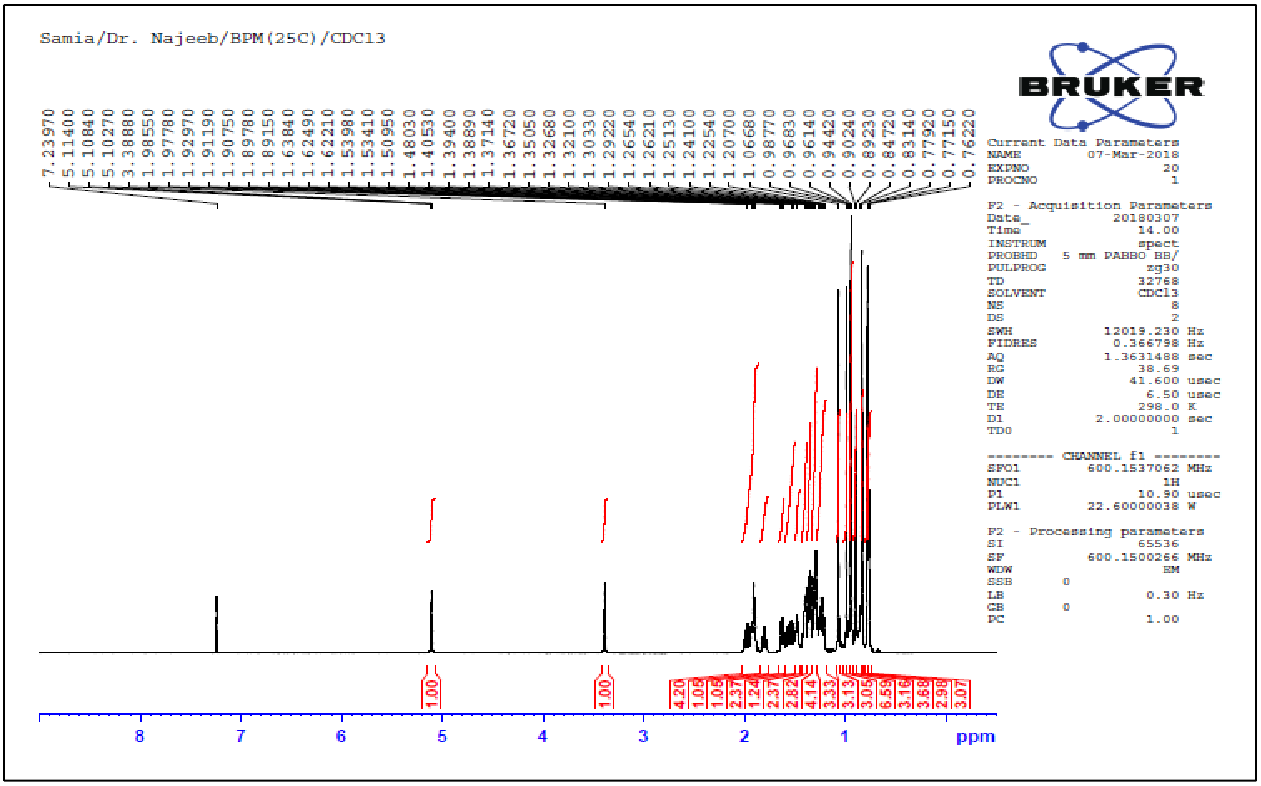

Figure S43. ^{1}H-NMR (600 MHz, $CDCl_3$) of compound **15**

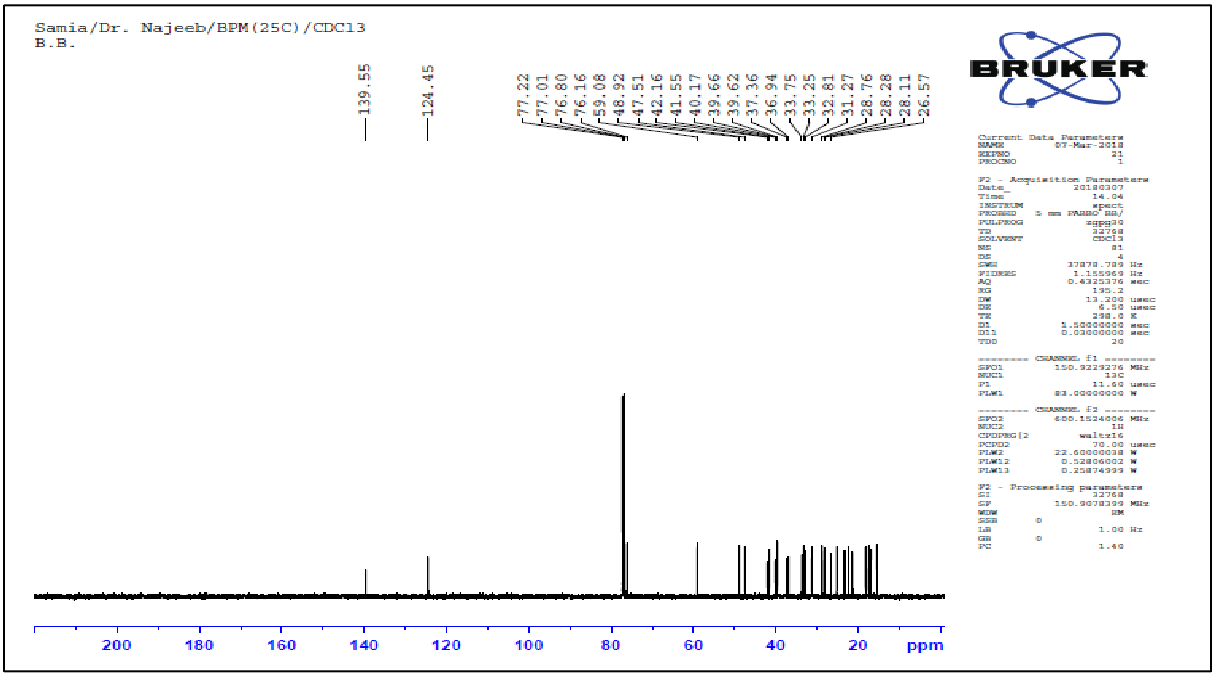

Figure S44. ^{13}C-NMR (150 MHz, $CDCl_3$) of compound **15**

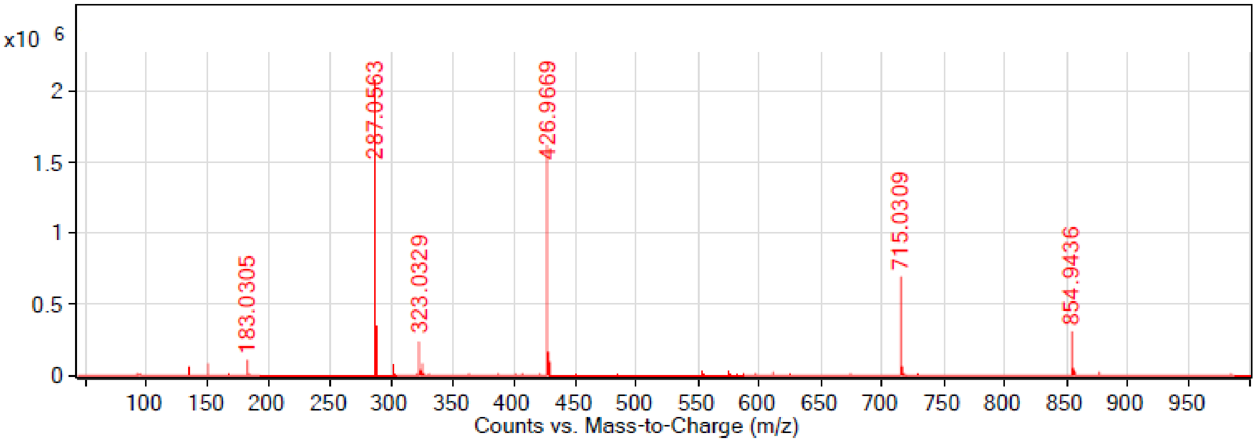

Figure S45. HRMS ((ESI$^+$)) of compound **15**

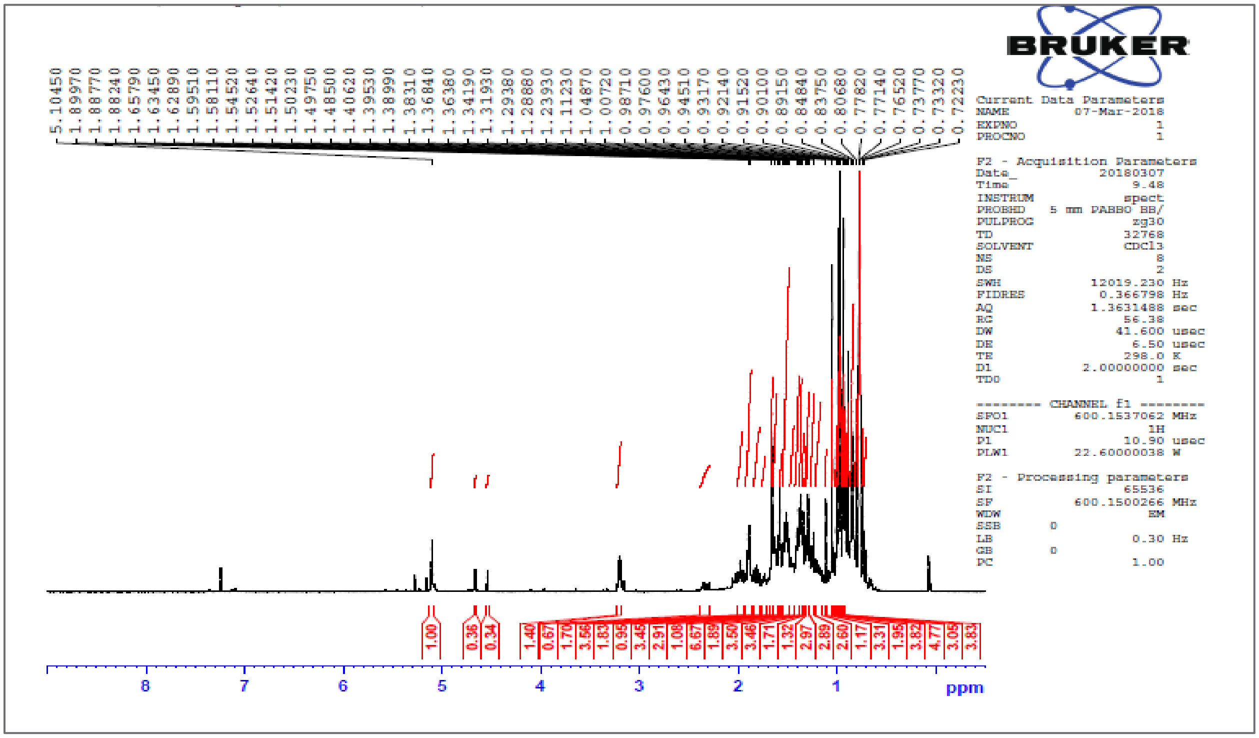

Figure S46. ^{1}H-NMR (600 MHz, $CDCl_3$) of compound **16**

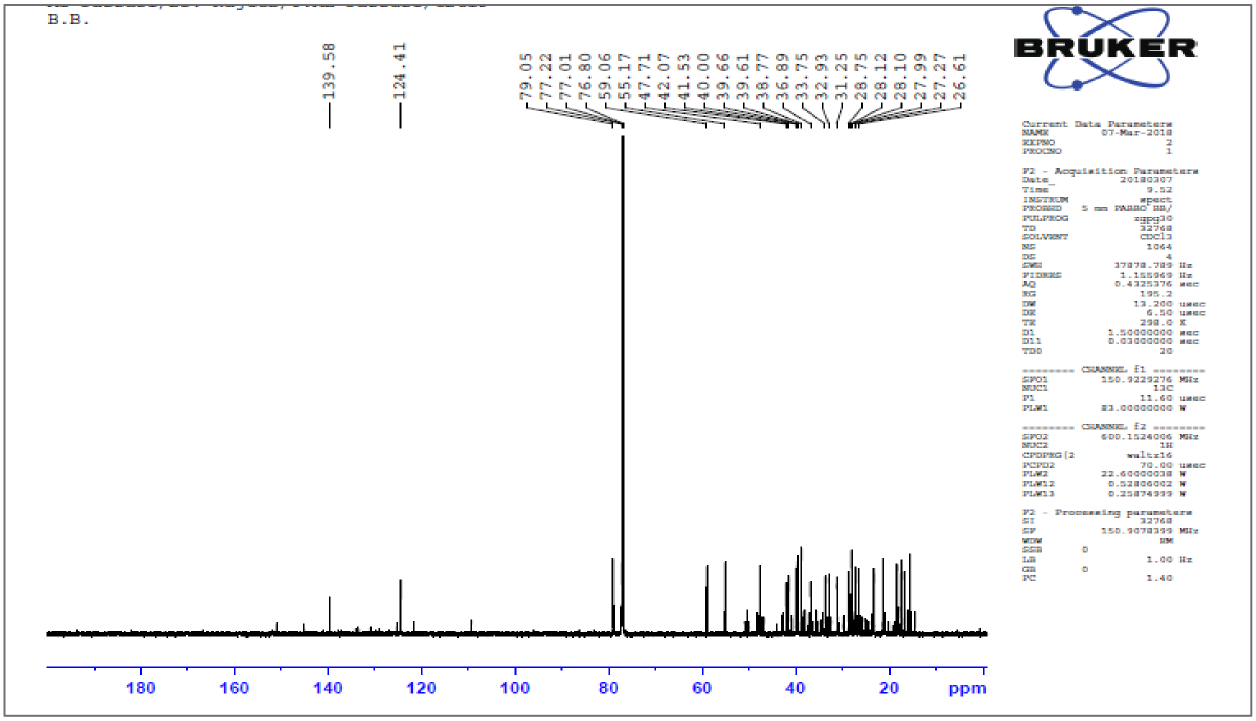

Figure S47. ^{13}C-NMR (150 MHz, $CDCl_3$) of compound **16**

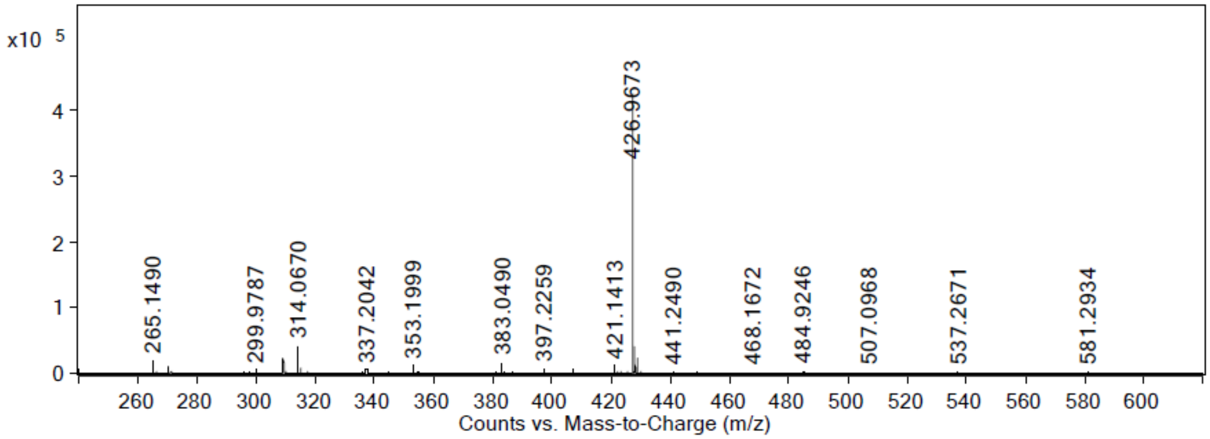

Figure S48. HRMS (ESI^+) of compound **16**

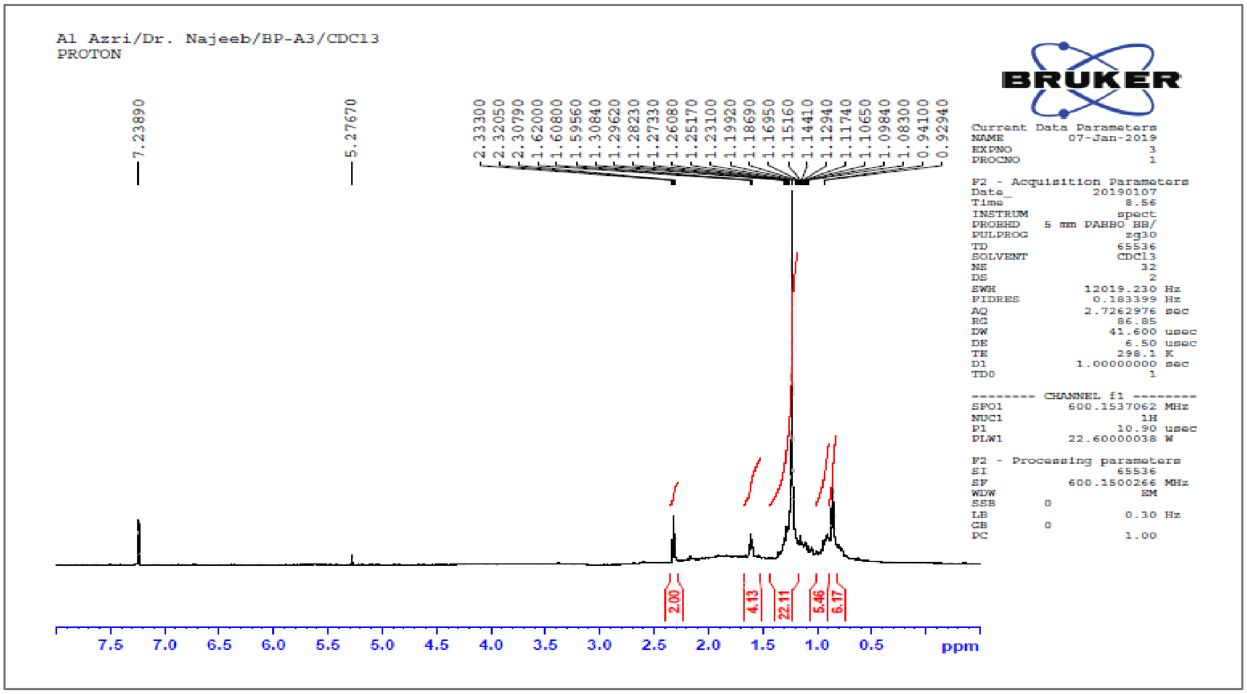

Figure S49. ^{1}H-NMR (600 MHz, $CDCl_3$) of compound **17**

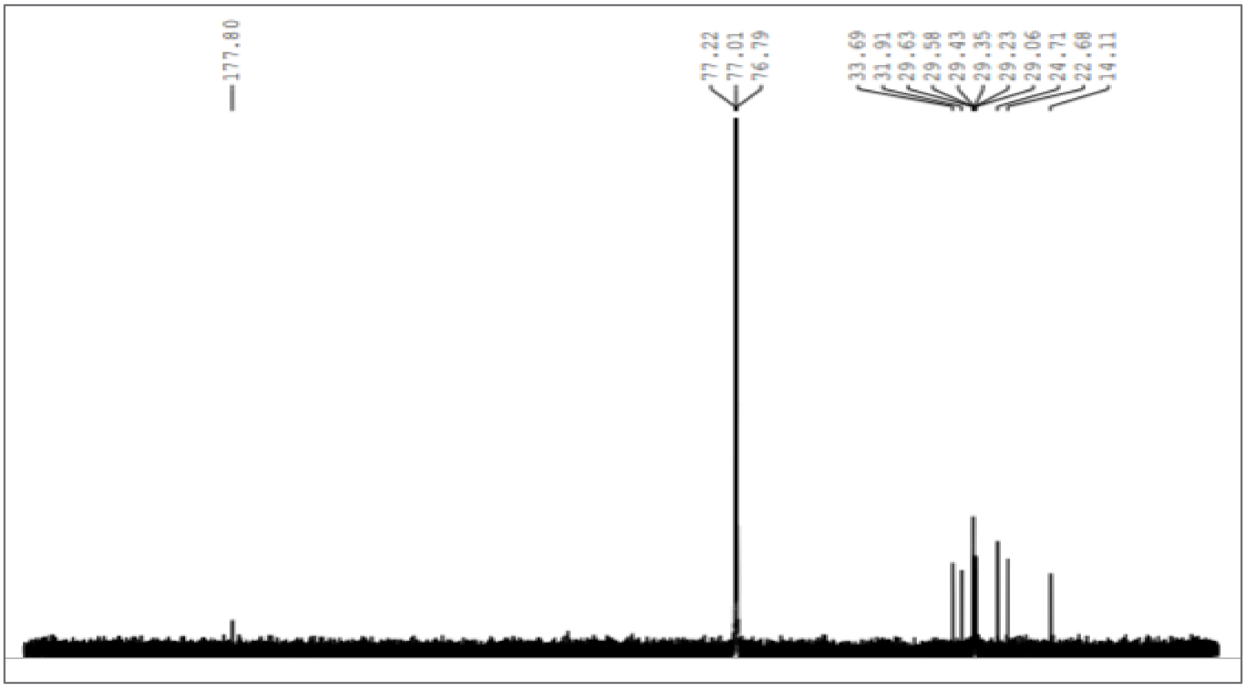

Figure S50. ^{13}C-NMR (150 MHz, $CDCl_3$) of compound **17**

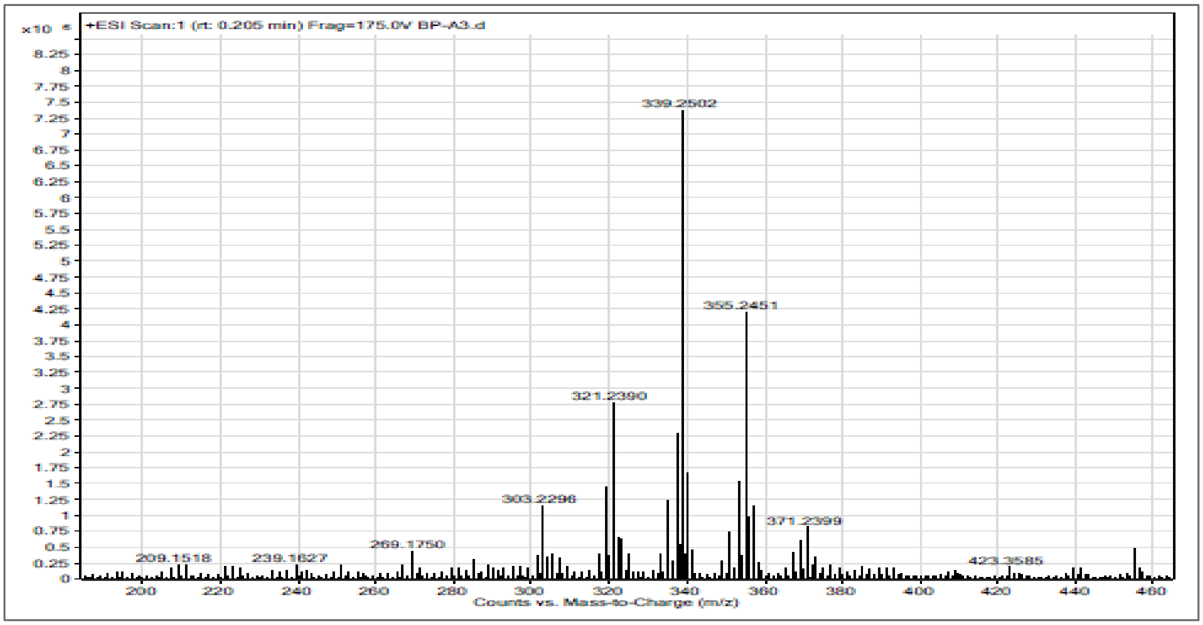

Figure S51. HRMS (ESI^+) of compound **17**

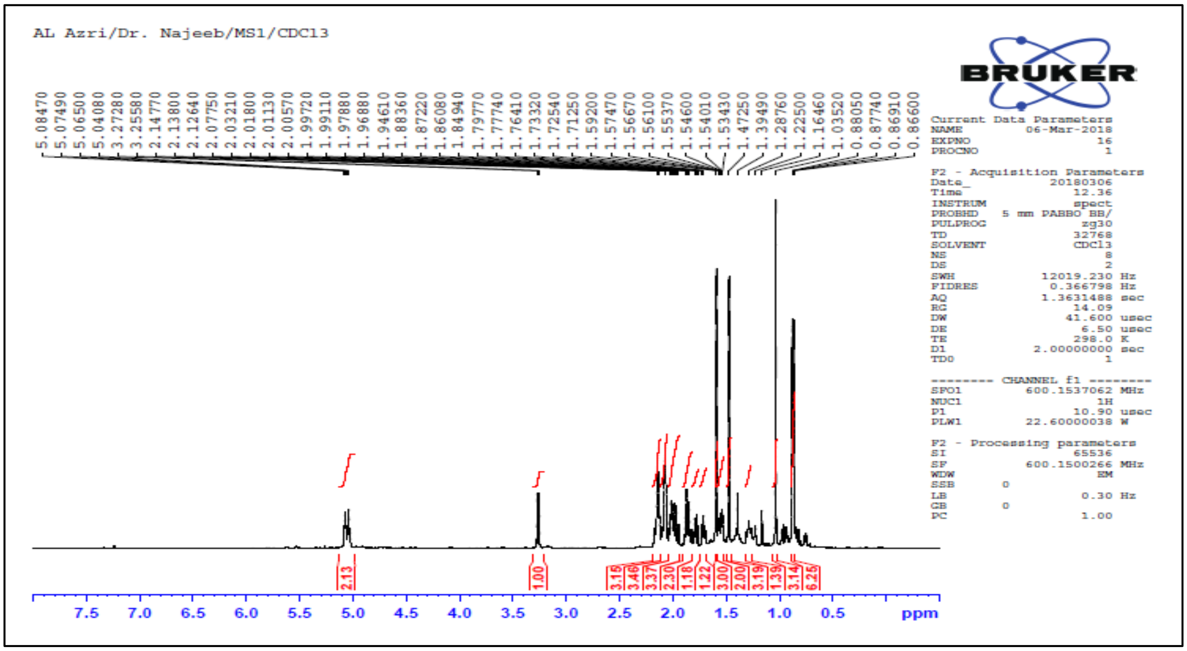

Figure S52. ^{1}H-NMR (600 MHz, $CDCl_3$) of compound **18**

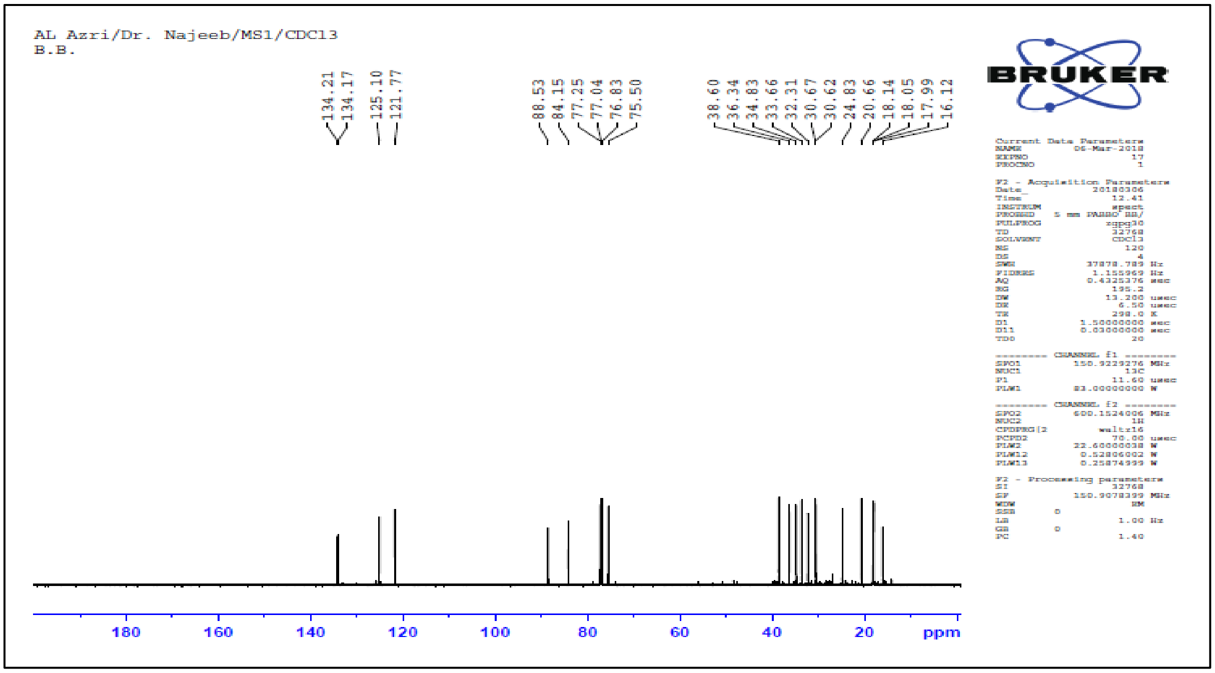

Figure S53. ^{13}C-NMR (150 MHz, $CDCl_3$) of compound **18**

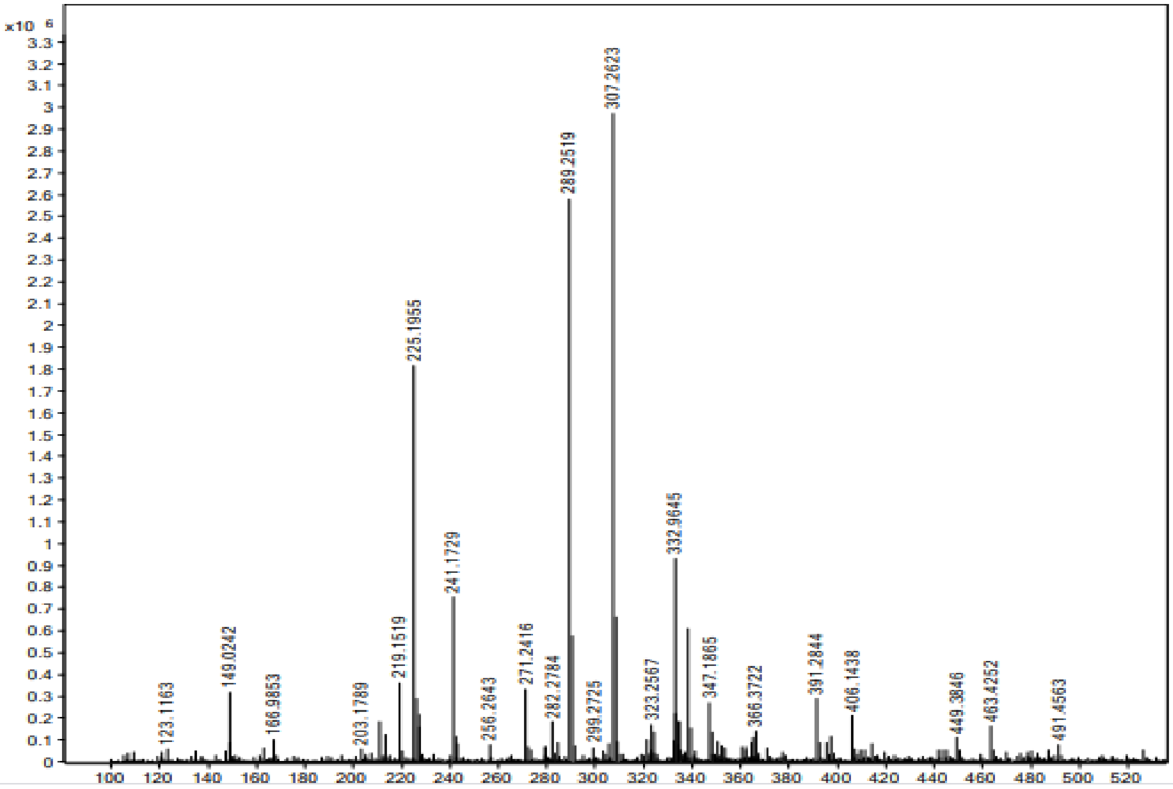

Figure S54. ESI-MS (positive) of compound **18**

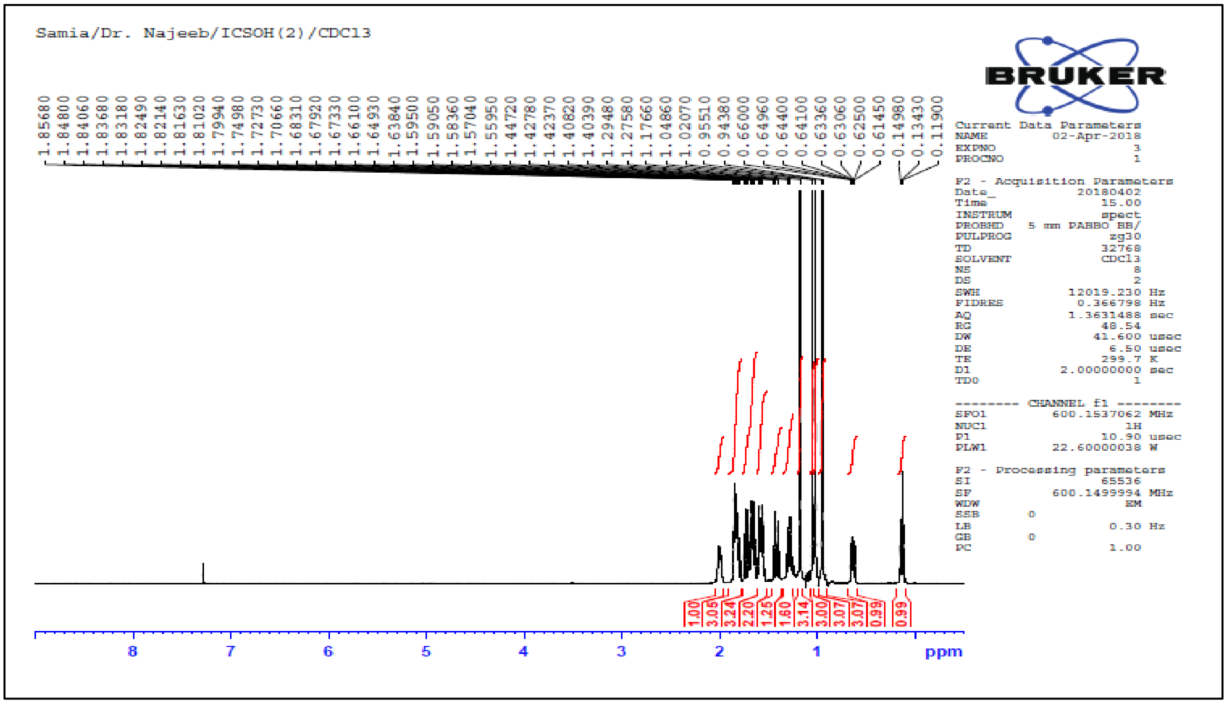

Figure S55. ^{1}H-NMR (600 MHz, $CDCl_3$) of compound **19**

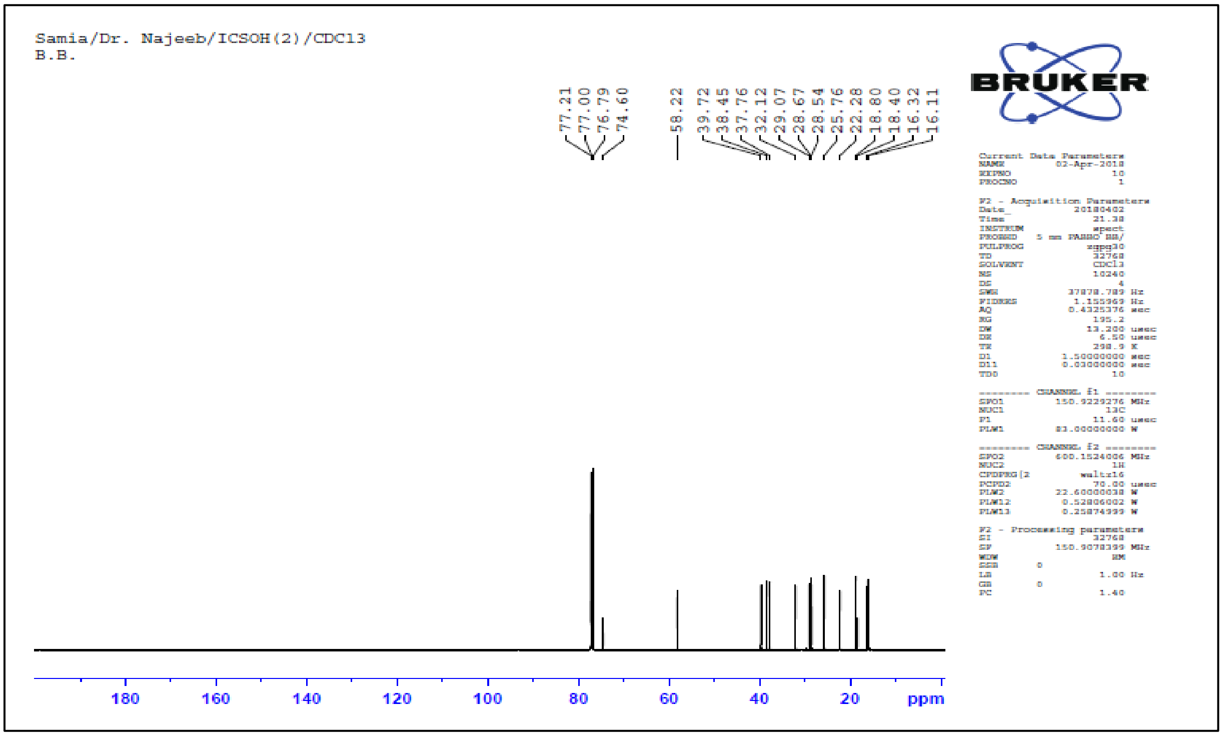

Figure S56. ^{13}C-NMR (150 MHz, $CDCl_3$) of compound **19**

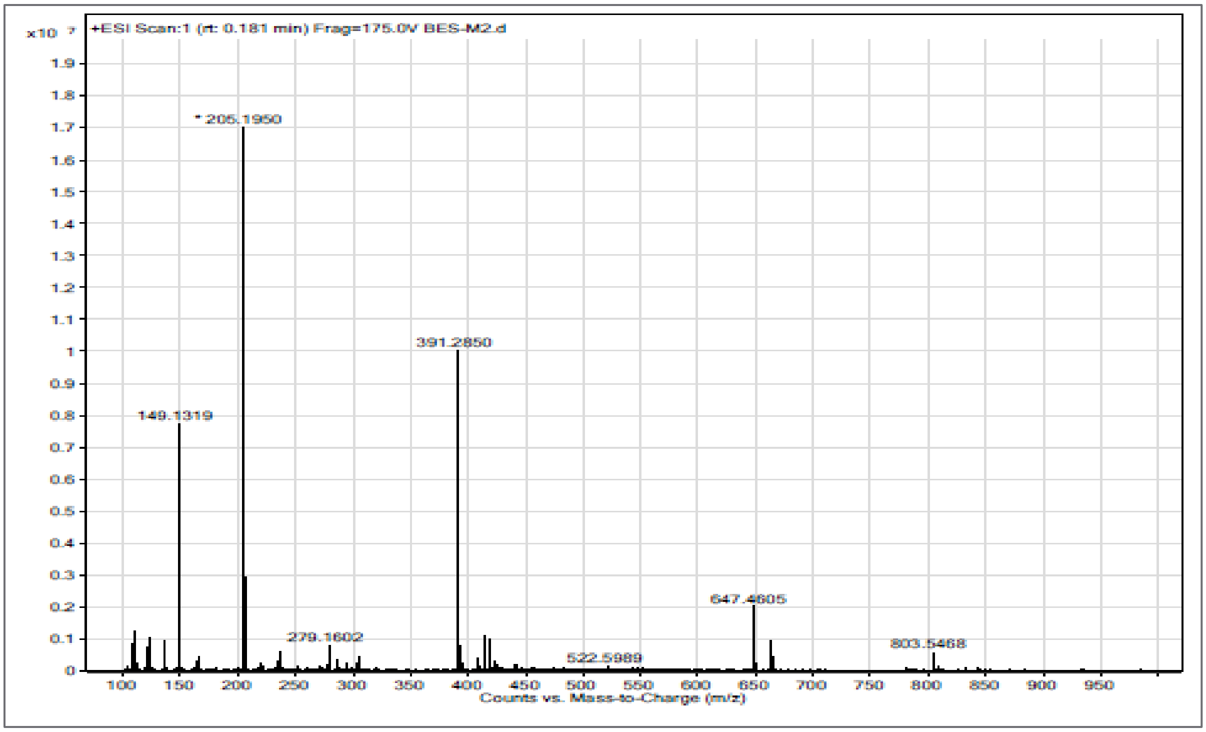

Figure S57. HRMS (ESI^+) of compound **19**

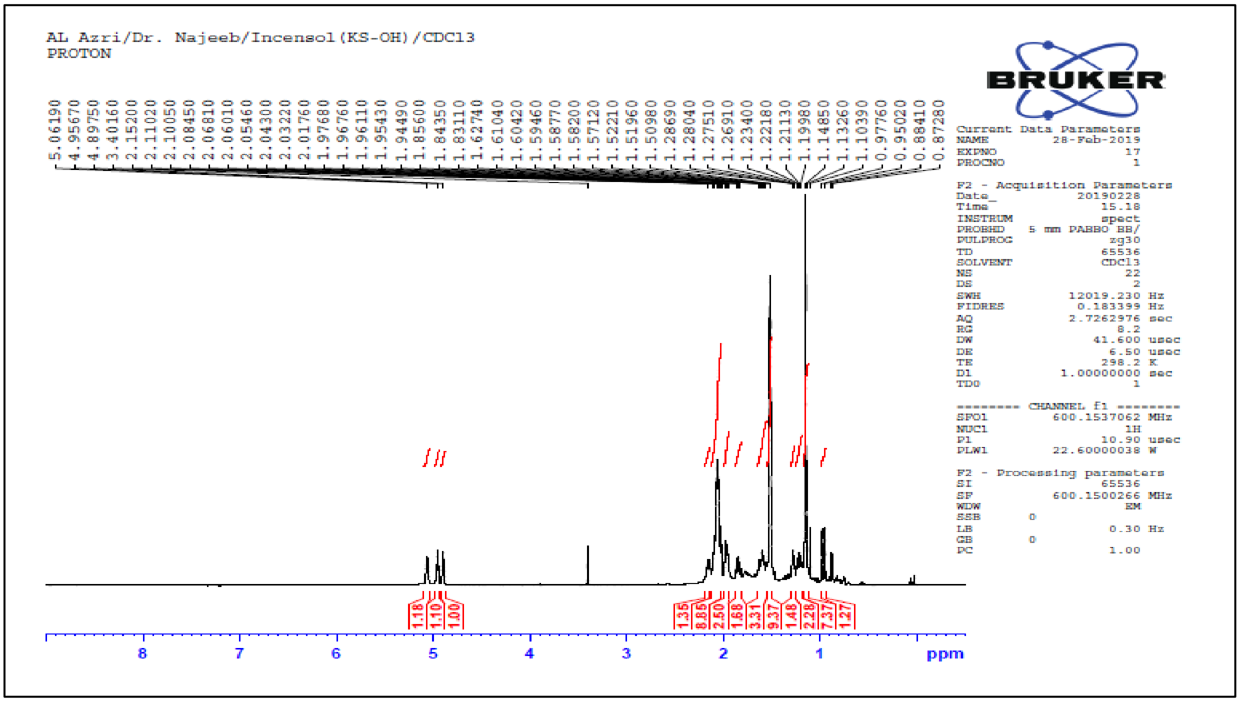

Figure S58. ^{1}H-NMR (600 MHz, $CDCl_3$) of compound **20**

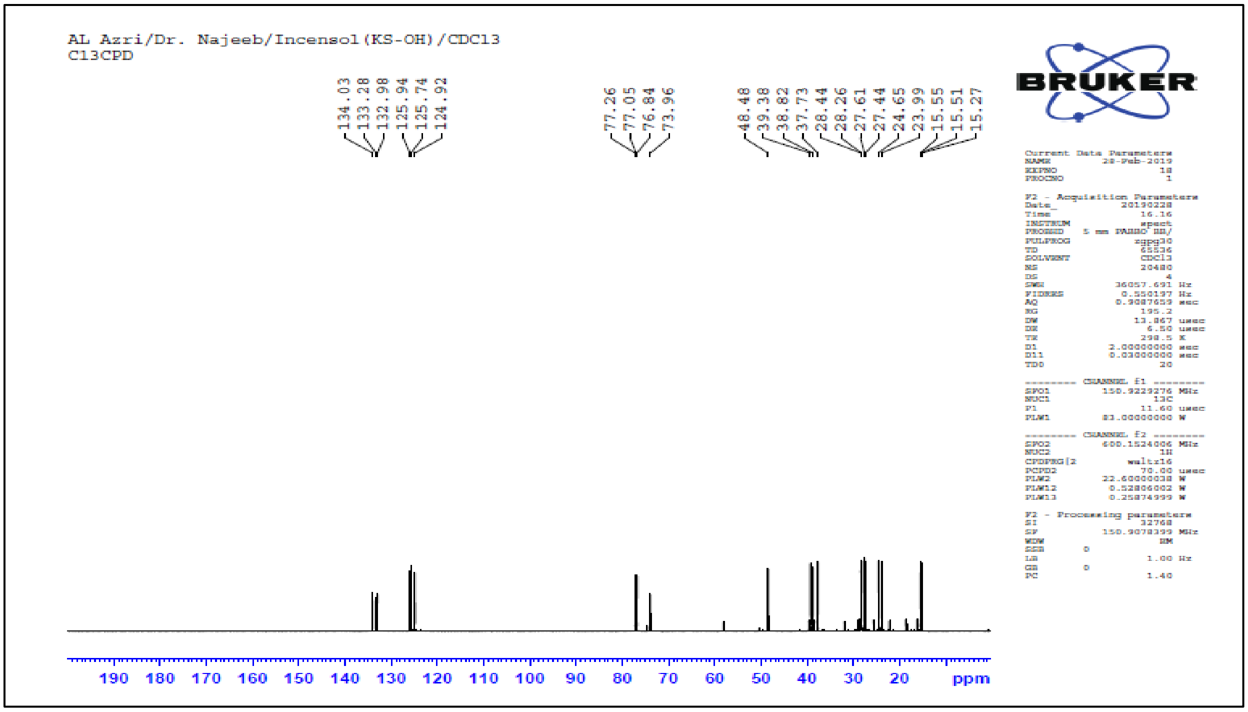

Figure S59. ^{13}C-NMR (150 MHz, $CDCl_3$) of compound **20**

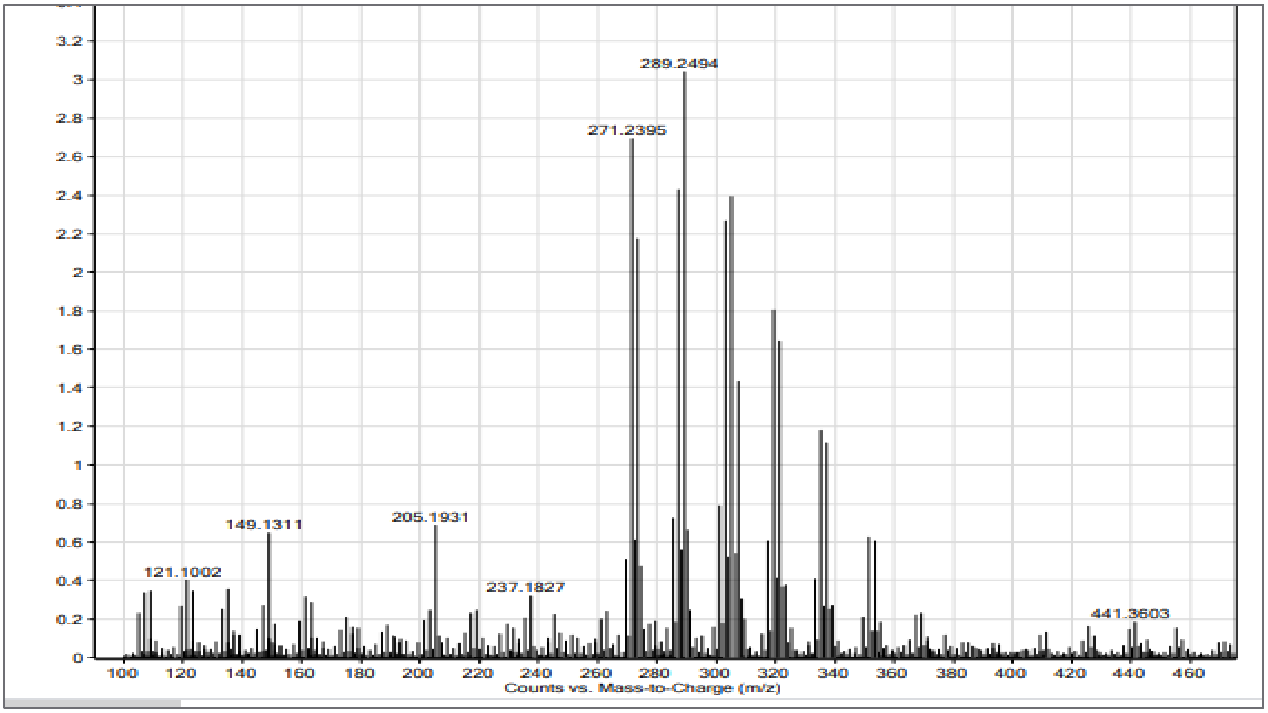

Figure S60. HRMS (ESI^{-}) of compound **20**

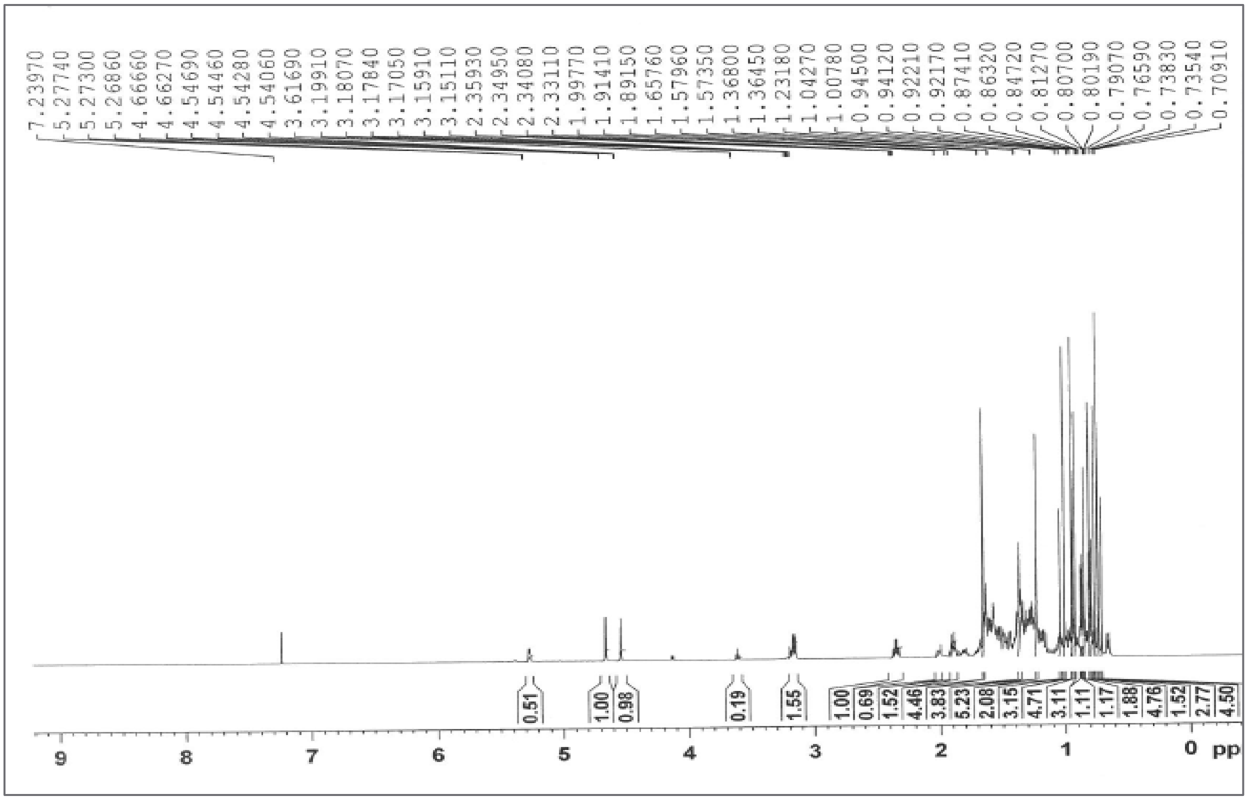

Figure S61. ^{1}H-NMR (600 MHz, $CDCl_3$) of compound **21**

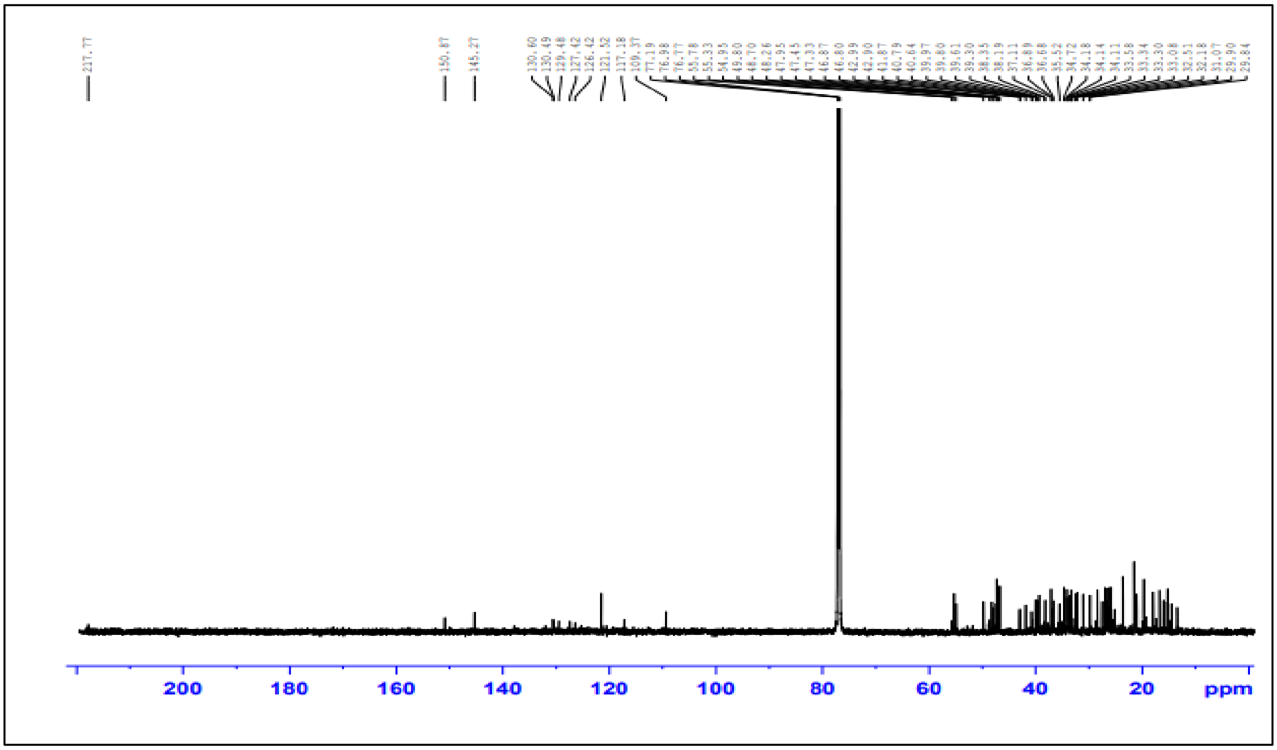

Figure S62. ^{13}C-NMR (150 MHz, $CDCl_3$) of compound **21**

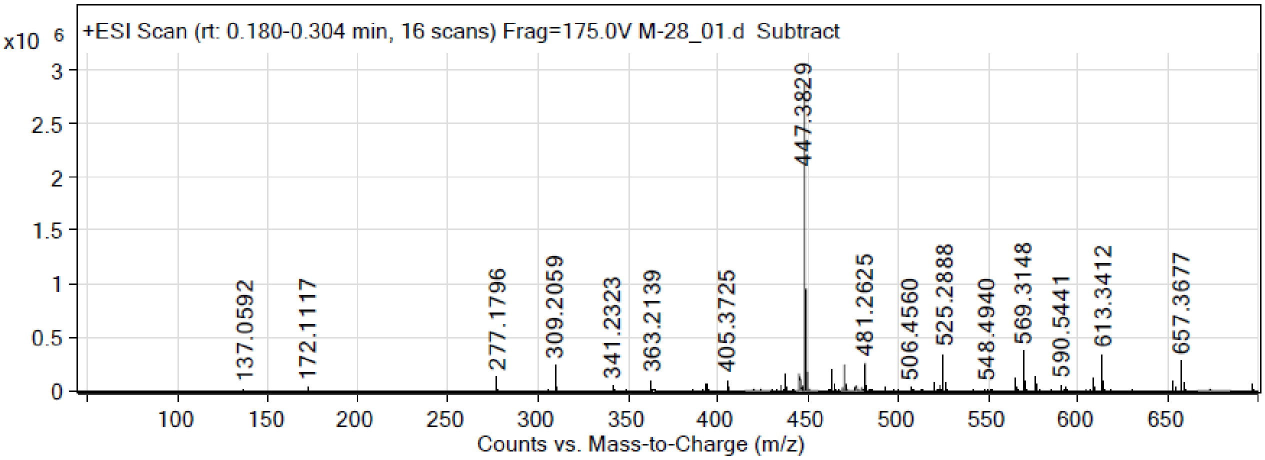

Figure S63. HRMS (ESI^+) of compound **21**
